# Blood Stage Malaria Vaccine Eliciting High Antigen-Specific Antibody Concentrations Confers No Protection to Young Children in Western Kenya

**DOI:** 10.1371/journal.pone.0004708

**Published:** 2009-03-05

**Authors:** Bernhards R. Ogutu, Odika J. Apollo, Denise McKinney, Willis Okoth, Joram Siangla, Filip Dubovsky, Kathryn Tucker, John N. Waitumbi, Carter Diggs, Janet Wittes, Elissa Malkin, Amanda Leach, Lorraine A. Soisson, Jessica B. Milman, Lucas Otieno, Carolyn A. Holland, Mark Polhemus, Shon A. Remich, Christian F. Ockenhouse, Joe Cohen, W. Ripley Ballou, Samuel K. Martin, Evelina Angov, V. Ann Stewart, Jeffrey A. Lyon, D. Gray Heppner, Mark R. Withers, for the MSP-1 Malaria Vaccine Working Group

**Affiliations:** 1 US Army Medical Research Unit-Kenya and the Centre for Clinical Research, Kenya Medical Research Institute, Nairobi, Kenya; 2 The PATH Malaria Vaccine Initiative, Bethesda, Maryland, United States of America; 3 Statistics Collaborative, Inc., Washington, D. C., United States of America; 4 Malaria Vaccine Development Program, US Agency for International Development, Washington, D. C., United States of America; 5 GlaxoSmithKline Biologicals s.a., Rixensart, Belgium; 6 Division of Malaria Vaccine Development, Walter Reed Army Institute of Research, Silver Spring, Maryland, United States of America; Walter and Eliza Hall Institute of Medical Research, Australia

## Abstract

**Objective:**

The antigen, falciparum malaria protein 1 (FMP1), represents the 42-kDa C-terminal fragment of merozoite surface protein-1 (MSP-1) of the 3D7 clone of *P. falciparum*. Formulated with AS02 (a proprietary Adjuvant System), it constitutes the FMP1/AS02 candidate malaria vaccine. We evaluated this vaccine's safety, immunogenicity, and efficacy in African children.

**Methods:**

A randomised, double-blind, Phase IIb, comparator-controlled trial.The trial was conducted in 13 field stations of one mile radii within Kombewa Division, Nyanza Province, Western Kenya, an area of holoendemic transmission of *P. falciparum*. We enrolled 400 children aged 12–47 months in general good health.Children were randomised in a 1∶1 fashion to receive either FMP1/AS02 (50 µg) or *Rabipur*® rabies vaccine. Vaccinations were administered on a 0, 1, and 2 month schedule. The primary study endpoint was time to first clinical episode of *P. falciparum* malaria (temperature ≥37.5°C with asexual parasitaemia of ≥50,000 parasites/µL of blood) occurring between 14 days and six months after a third dose. Case detection was both active and passive. Safety and immunogenicity were evaluated for eight months after first immunisations; vaccine efficacy (VE) was measured over a six-month period following third vaccinations.

**Results:**

374 of 400 children received all three doses and completed six months of follow-up. FMP1/AS02 had a good safety profile and was well-tolerated but more reactogenic than the comparator. Geometric mean anti-MSP-1_42_ antibody concentrations increased from1.3 µg/mL to 27.3 µg/mL in the FMP1/AS02 recipients, but were unchanged in controls. 97 children in the FMP1/AS02 group and 98 controls had a primary endpoint episode. Overall VE was 5.1% (95% CI: −26% to +28%; p-value = 0.7).

**Conclusions:**

FMP1/AS02 is not a promising candidate for further development as a monovalent malaria vaccine. Future MSP-1_42_ vaccine development should focus on other formulations and antigen constructs.

**Trial Registration:**

Clinicaltrials.gov NCT00223990

## Introduction

*Plasmodium falciparum* malaria kills over one million children annually in sub-Saharan Africa [1] where it is considered both a cause and a consequence of poverty [2]. In the last decade several malaria vaccine candidates have progressed to clinical evaluation [3]. RTS,S/AS02, a pre-erythrocytic stage *falciparum* malaria vaccine, showed reductions of 35% for clinical disease and 49% for severe disease over an 18 months follow up period, in one to four year old Mozambican children [4], [5], confirming the feasibility of malaria vaccines and their potential to impact the burden of disease in infants.

A second promising *P. falciparum* vaccine target is the blood stage antigen merozoite surface protein-1 (MSP-1) [6], [7]. This abundant merozoite membrane surface protein undergoes a series of processing steps, with the final cleavage of the C-terminal p42-kDa portion of the molecule into p33-kDa and p19-kDa molecules required for erythrocyte invasion [8]. Monoclonal antibodies that specifically interfere with this cleavage can inhibit parasite invasion [9]. Epidemiologic studies have shown that antibodies directed against this part of MSP-1 are associated with protection [10], [11], [12]. *Aotus* monkeys vaccinated with MSP-1_42_ have been protected from *P. falciparum* challenge [13], [14] and antibodies derived from such animals have exhibited *in vitro* growth inhibition activity [15]. A previous study of MSP1 and MSP2 recombinant proteins combination malaria vaccine candidate (Combination B) showed a significant reduction in parasite density in the vaccinated group but no significant effect on clinical episodes [16]. However, multiple inoculations with subinfective doses of whole cell infected cryopreserved erythrocuytes followed by immediate antimalarial treatment conferred T cell mediated protection against infection in a challenge study [17].

MSP-1_42_ from the 3D7 clone of *P. falciparum* was expressed in *Escherichia coli*. The final product, designated falciparum malaria protein 1 (FMP1) [18], has been formulated with GlaxoSmithKline Biologicals' (GSK) proprietary Adjuvant System AS02. Earlier studies showed the FMP1/AS02 formulation to be safe and immunogenic in rhesus monkeys [19] and in adult humans in the United States [20], Kenya [21], and Mali [22]. Subsequently, a Phase Ib dose-escalating trial demonstrated its safety and immunogenicity in 12 to 47 month old Kenyan children [23]. The 50 µg dose gave a 16-fold rise in the geometric mean titre (GMT) of anti-MSP-1_42_ antibodies from baseline to one month after a third vaccination. These encouraging results led to the present proof-of-concept efficacy trial in the same population, which is the first to evaluate monovalent MSP-1_42_ for efficacy in a field setting.

## Methods

The protocol for this trial and the supporting CONSORT checklist are available as supporting information; Checklist S1 and Protocol S1.

### Study site

The study was conducted at the KEMRI-“Walter Reed Project” (WRP) Kombewa Clinic (KC) in the Kombewa Division of Kisumu District of Nyanza Province in western Kenya, an area of hyperendemic malaria transmission with peaks during the long rains (March–June) and short rains (November–December). The study population, almost exclusively of the Luo tribe, predominantly Seme sub-tribe, was drawn from one mile radius catchment areas around each of 13 field stations. The field stations were staffed 24 hours/day throughout the study period to facilitate medical care. *P. falciparum* accounts for more than 95% of the malaria infections [24] with mosquitoes of the *Anopheles gambiae* complex being the major vector [25] with an EIR of 0.65–0.79 infectious bites per person per night [24], [25].

### Participants

Demographic surveillance was conducted at the Kombewa Division field stations in 2003–2004. During the census, the families with children aged 12–47 months were identified and homesteads located in relation to the field stations. At the time of the study initiation, parents/guardians were invited to come to the KC for briefing and those who gave consent were later requested to bring their children to the KC for eligibility screening. Children were excluded according to the same criteria used in the previous paediatric Phase Ib study [21], except for the slight modifications of serum alanine aminotransferase (ALT) of ≥45 IU/L and absolute lymphocyte counts (ALC) for one-year olds of <4.0×10^3^/mm^3^, for two-year olds of <3.0×10^3^/mm^3^, and for three-year olds of <2.0×10^3^/mm^3^.

Groups that approved the study protocol were: Kenya Medical Research Institute (KEMRI) Scientific Steering Committee and Ethical Review Committee, Walter Reed Army Institute of Research (WRAIR) Scientific Review Committee, US Army Medical Research and Materiel Command (USAMRMC), Human Subjects Research Review Board (HSRRB), and the PATH Human Subject Protection Committee (HSPC). The United States Food and Drug Administration (FDA) reviewed the protocol. The study was conducted in accordance with the International Conference on Harmonisation Guideline for Good Clinical Practice. Pharmaceutical Product Development, Inc. (PPD) and the US Army Medical Materiel Development Activity (USAMMDA) monitored the study. An independent Local Medical Monitor (LMM) and a data and safety monitoring board (DSMB) closely reviewed the trial's progress.

### Procedures

Before recruiting study participants, representatives of the study team met with local chiefs and community leaders to describe the study. Field workers then visited individual homesteads known to have potential study participants and invited the parents or guardians to visit the clinic for recruitment briefings. At each briefing session parents/guardians received a copy of the informed consent form (ICF). Groups watched a videotape detailing (in the Luo language) the study and information on the ICF, which was followed by a public question and answer session. Parents/guardians were then invited for private individual discussions with a study physician assistant or study nurse, after which they were invited to sign (or thumb print if illiterate) the ICF. For each illiterate parent/guardian, an impartial member of the community countersigned as a witness.

During the screening visits, within 45 days before the first immunisation, the children had a detailed clinical history, physical examination, and blood sampling for haematology and biochemistry evaluations. The children were randomised on day 0 and received the vaccine on days 0, 29 (±9) and 57 (±9). After each vaccination the children were evaluated for solicited adverse events (AEs) on days 0, 1, 2, 3, and 6. The unsolicited AE follow-up periods ran for 30 days (vaccination days and the subsequent 29 days). Serious adverse events (SAEs) were reported throughout the duration of the study. During the efficacy follow-up period, all SAEs were reported within 24 hours of occurrence. In the event that a child was treated outside the WRP KC or the New Nyanza Provincial General Hospital copies of all available clinical records of the encounter were obtained, reviewed and maintained in the subject's record. When a study subject was admitted to NNPGH, a member of the clinical team visited the subject at least once daily and reported to the principal investigator (PI) or his designee. The PI remained in contact with the paediatrician or any other medical specialist who provided care to the subject to ensure that the locally appropriate medical care was provided.

During the follow-up period, clinical malaria cases were tracked and parasite densities and immunogenicity measured. Surveillance was performed on a biweekly basis through monthly field worker home visits and monthly clinic visits (window period: ±7 days) (i.e., the clinic and home visits were staggered 2 weeks apart hence the child was seen twice per month). During the home visits to the children field workers documented axillary temperatures, solicited history of fever in the previous 24 hours and of any concomitant medications taken, and reminded parents/guardians of future appointments and the necessity of bringing sick children to the KC. Children with a fever or history of fever in the past 24 hours were taken to the KC for evaluation, including malaria blood films (MBFs) and complete blood counts (CBCs). Out-migrations, absences from Kombewa Division, and antimalarial drugs usage were documented.

**Figure 1:**
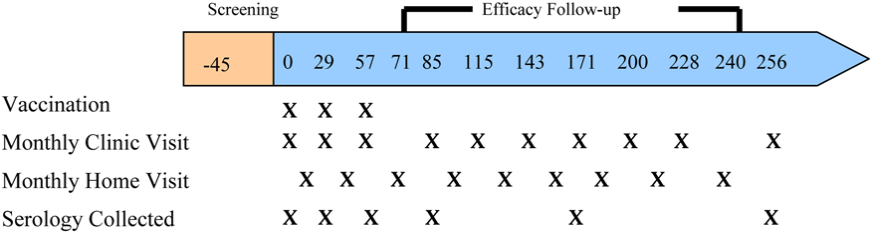
Schematic of trial profile.

Clinic and home visits were staggered so that each child was evaluated (active detection) about every fortnight. During the clinic visits, an interim history and an axillary temperature were obtained, a history-directed clinical examination was performed, and blood was drawn (Figure 1).

Clinical malaria was also identified by passive detection (when ill children presented to the KC or to field stations). Blood samples for CBC and MBF were obtained whenever an ill child presented to the clinic. At study start, parents were advised to seek medical attention for the children exclusively at the KC whenever possible Children requiring inpatient care were admitted to the NNPGH. Uncomplicated malaria was treated with a 6 dose course of artemether/lumefantrine (Coartem®) and quinine in case of severe malaria.

#### Laboratory procedures

Two microscopists independently read Giemsa-stained thin and thick malaria blood films (MBFs) and quantified them according to a standard operating procedure. Results were double data entered into a laboratory database; a third, more experienced reader resolved any discordant microscopy findings. Parasite densities were calculated against same-day CBCs.

Induction of humoral immunity was measured with an enzyme-linked immunosorbent assay (ELISA) using purified bulk FMP1 (MSP-1_42_ 3D7) as the capture antigen [20]. Results are reported in µg/mL, derived by comparison to a defined standard [26]. The KEMRI/“WRP” Kondele unit Laboratory in Kisumu performed the ELISA analysis and associated quality control assays; additional quality control analyses were performed at the Malaria Serology Laboratory, WRAIR, USA.

### Interventions

#### Vaccines

The FMP1 antigen, described in detail elsewhere [20], was manufactured according to current Good Manufacturing Practices (cGMP) at the WRAIR pilot Bioproduction facility in Silver Spring, MD, USA. The AS02 Adjuvant System, manufactured by GSK Biologicals, contains an oil-in-water emulsion and components also described elsewhere [20]. A vial containing the lyophilised 60 µg FMP1 pellet was reconstituted in 0.6 mL of AS02 and, after mixing, a syringe containing 0.5 mL was prepared for injection. The vaccine was administered immediately after reconstitution. The comparator group received the licensed rabies vaccine *Rabipur*® (manufactured in India by Chiron Behring Vaccines Pvt, Ltd) supplied in single dose vials containing lyophilised antigen with 1.0 mL of diluent (sterile water) for injection.

#### Vaccine administration

After confirming eligibility, staff drew blood for laboratory tests. The pharmacy team confirmed individual assignments to vaccine groups and then prepared the appropriate vaccine. Because the vaccines differed in volume and appearance, the pharmacy team masked the barrel of the syringe, labeled the syringe with each individual's unique randomisation code and study ID number, and passed the syringe through a small service hatch into the vaccination room.

A vaccine administration team confirmed each child's identity, study number, and randomisation code before administering vaccine. A nurse administered the vaccine by slow intramuscular injection into the left anterolateral thigh. Vaccinations were performed on a 0, 1, and 2 month schedule (Figure 1). The nurses administering the vaccine were not involved in the post-vaccination assessments.

#### Post-vaccination procedures

Children were observed for 60 minutes after each vaccination. Parents/guardians were instructed to return with children to the KC immediately for any new symptoms. The study team evaluated the children on days 1, 2, 3, and 6 after each vaccination to collect solicited (pain, swelling, fever, irritability/fussiness, loss of appetite, and drowsiness) and unsolicited AEs. If a parent and child did not appear for a scheduled clinic visit, a clinician or nurse visited them on the same day to measure vital signs and collect information on any AEs that occurred during the previous 24 hours. Transportation was provided for the participants to KC, where AEs were evaluated, monitored, and clinically managed until resolution.

### Objectives

The primary objective was to evaluate the vaccine efficacy (VE) against first clinical episodes of *P. falciparum* malaria over a six month period commencing 14 days after a third vaccination. Clinical malaria for the primary endpoint was defined as parasite density ≥50,000 parasites/µL n the presence of fever (axillary temperature ≥37.5°C). This was based on results of an unpublished epidemiology study in children of the same group prior to the study.

Secondary objectives were to evaluate: (1) clinical episodes of *P. falciparum* malaria using alternate case definitions; (2) parasite density; (3) safety as evaluated by solicited adverse events (AEs) occurring in the week following each vaccination, unsolicited AEs occurring within 30 days of each vaccination, serious adverse events (SAEs) occurring throughout the study, and time to anaemia (Hb<8.0 g/dL); (4) immunogenicity as assessed by geometric means of anti-MSP-1_42_ antibody concentration throughout the study; (5) the immunogenicity from first vaccination through the end of the follow-up period; and (6) association between anti-MSP-1_42_ antibody concentration and the subsequent risk of clinical malaria during the follow-up period.

### Outcomes

#### Assessment of primary outcome

The primary outcome was designated as time to first clinical episode of *P. falciparum* malaria meeting the “Primary Case Definition” (Table 1) during the six month efficacy follow-up period. Time was measured from 14 days after administration of a third dose of study vaccine. The logrank statistic and corresponding *p*-value was calculated to test the null hypothesis of no difference in the distribution of time to clinical malaria between the FMP1/AS02 and rabies vaccine groups. A Cox proportional hazards model with an indicator variable for vaccine group was fit to the data and an estimate of the hazard ratio along with its corresponding 95% confidence interval was computed. VE was defined as one minus the hazard ratio. Additional Cox models explored the effects of age, sex, bed net use as reported at baseline, nearest field station, and haemoglobin genotype on vaccine efficacy. Kaplan-Meier curves for time to clinical malaria were generated by vaccine group. An alternative estimate of VE based on cumulative incidence rates was calculated as well. The primary data analysis was conducted with the according-to-protocol cohort; however, a similar analysis based on the intention-to-treat cohort was also performed to assess the robustness of the analysis based on the according-to-protocol cohort.

**Table 1:** Malaria case definitions and vaccine efficacy in the according-to-protocol group.

Clinical Malaria								
*Malaria Definitions*				*Vaccine Efficacy*				
*Case Definition*	*Fever* a		*Parasite density* b	*FMP1/AS02* *(N = 195)*	*Rabipur*® *(N = 190)*	*Vaccine efficacy*	*95% confidence interval*	*p-value*
Secondary (200)	≥37.5°C	AND^c^	≥200 K	44	40	−6.8	(−64, 30)	0.8
Secondary (100)	≥37.5°C	AND	≥100 K	75	75	1.6	(−36, 29)	0.9
**Primary**	**≥37.5°C**	**AND**	**≥50 K**	**97**	**98**	**5.1**	**(−26, 28)**	**0.7**
Secondary (10)	≥37.5°C	AND	≥10 K	106	108	6.5	(−22, 29)	0.6
Secondary (0)	≥37.5°C	AND	>0	132	138	12.3	(−11, 31)	0.3
Secondary (0*)	≥37.5°C OR history of fever	AND	>0	173	168	5.3	(−17, 23)	0.6

Malaria Infection (Symptomatic or Asymptomatic)**						
*Malaria Definitions*		*Vaccine Efficacy*				
*Case Definition*	*Parasite density*	*FMP1/AS02*	*Rabipur®*	*Vaccine efficacy*	*95% confidence interval*	*p-value*
Infection definition	Parasite density >0	173 [192]	170 [184]	7.7	(−14, 25)	0.5
Infection definition (50)	Parasite density ≥50 K	128 [190]	128 [183]	12.5	(−12, 32)	0.3

* History of fever within the last 24 hours.

** Children who met these infection endpoints on the first day of the efficacy follow-up period were excluded from these analyses. Numbers of children at risk are shown in brackets.

a Fever is documented by axillary temperature.

b Asexual stage *P. falciparum* parasites/μl blood; K denotes thousands.

c AND means “simultaneous presence of.”

#### Assessment of secondary outcomes

The purpose of the secondary outcomes was to gain further insight into the efficacy of the vaccine and to help define a primary endpoint for a future, confirmatory Phase III trial. These were not intended for use to make formal claims about the vaccine. Other secondary endpoints assessed safety and immunogenicity of the vaccine. Similar analyses as those outlined for the primary endpoint were carried out for the following endpoints.

Measurements of efficacy included (1) time to clinical episode of *P. falciparum* malaria meeting the various secondary case definitions (Table 1); (2) time to presence of asexual stage *P. falciparum* parasites; (3) time to parasite density ≥50,000 asexual stage *P. falciparum* parasites/µL of blood; (4) number of clinical episodes of malaria, per subject, meeting the primary case definition; statistical methods for recurrent event data, such as Poisson regression, were used to assess the effect of FMP1/AS02 vaccination on the average number of clinical episodes per subject; (5) time to anaemia (defined as Hb<8.0 g/dl, whether by active or passive case detection); (6) proportion of children with anaemia.

Assessment of safety included (1) occurrences of solicited AEs during a seven-day follow-up period after each vaccination; (2) occurrences of unsolicited AEs during a 30-day follow-up period after each vaccination; and (3) occurrences of SAEs between the first vaccination and the end of the follow-up period. Assessment of immunogenicity included geometric means of anti-MSP-1 titers at first, second, and third vaccinations and at 30, 115, and 200 days after the third vaccination.

### Sample size

A year-long (2003–4) longitudinal cohort study in the same area showed monthly clinical malaria attack rates of between 20% and 55% in children aged one to three years (unpublished data). The sample size calculation for this trial assumed a 50% attack rate over the six-month follow-up period with time to clinical malaria following an exponential distribution. A trial with 200 children per study arm would have 80% power to detect a 30% reduction in the six-month cumulative incidence rate.

### Randomisation—sequence generation, allocation concealment, implementation

A randomisation list containing sequential codes linked to a study vaccine assignment was computer generated in the USA by Statistics Collaborative, Inc. These codes were assigned to participants in the order in which they presented to the clinic on the day of first vaccination. The code was then supplied to the study drug manager and the site pharmacist, who were responsible for randomising the study participants. Randomisation was performed within blocks of eight. Each randomisation assignment was sealed in a unique, tamper-evident envelope, which was opened at the time a subject presented for the first vaccination. At the end of every vaccination day, the randomisation list was secured in a locked safe accessible only to the study drug manager, the pharmacist, and the pharmacist's assistants. The only other person in Kenya who had access to the randomisation code at the study site was the LMM for use in case emergency unblinding became necessary for safety purposes. Four hundred children were randomised in the order of presentation to the clinic on the days of first vaccination; 200 received FMP1/AS02 and 200 *Rabipur*® rabies vaccine.

### Blinding

At the study site, only the pharmacist, pharmacy assistant and the drug manager were aware of the randomisation codes of the children. Children, parents, investigators, lab personnel and all staff performing follow-up evaluations were blinded as to vaccine assignment. Because the color and volumes of the reconstituted FMP1/AS02 and comparator vaccines differed, the barrel of the syringe was covered with opaque tape to mask its contents and labeled with the subject identification number and randomisation code. Children, parents, and the staff performing follow-up evaluations were all blinded. Vaccinations were carried out simultaneously in four separate consultation rooms, which were connected to a central pharmacy (the vaccine preparation room) by small, closable service hatches. On vaccination days, the prepared syringe was handed through a service hatch to a vaccinator for vaccine administration. For each subject, an identification number, a randomisation code from a chart, and a randomisation code on the syringe were recorded on a vaccination form. Following vaccine administration, children were assessed and follow-up visits conducted by a group of clinicians who had not been involved in the vaccinations.

### Statistical analysis

Data were recorded on case report forms that underwent 100% verification by a quality assurance team before double data entry into a *SQL Server 2000*® database. The database also underwent 100% verification by a quality assurance team before the archiving and transmission to the statisticians. Data analysis was performed on the locked database using *SAS* (version 9.1) and *S-Plus* (version 6.2) according to a prewritten statistical analysis plan.

The according-to-protocol cohort included all children who received the three vaccinations and had no significant protocol violation. Analyses of efficacy were based on the according-to-protocol cohort, counting each subject's first malaria case that emerged at least 14 days after a third vaccination to the end of the follow-up period. The intention-to-treat cohort included all randomised children. Supportive analyses, based on the intention-to-treat cohort, counted all cases that emerged from the day of first vaccination.

The primary endpoint was analyzed with a logrank test. A Cox proportional hazards model (SAS Proc PHREG) was used to estimate the hazard ratio along with its corresponding 95% confidence interval: VE is one minus the hazard ratio. Similar analyses were performed for other case definitions. Additional Cox models explored the effects of age, sex, baseline bednet use, field station location, and haemoglobin genotype on VE. Kaplan-Meier curves displayed time to event. An alternative estimate of VE based on cumulative incidence rates, excluding time periods when the subject was not at risk for contracting a new case of malaria, was calculated using Poisson regression. Fisher's Exact Test compared binary variables. Parameters measuring immune response were calculated and compared between groups using longitudinal models (SAS Proc MIXED) with a spatial power covariance structure.

## Results

### Participant flow

**Figure 2:**
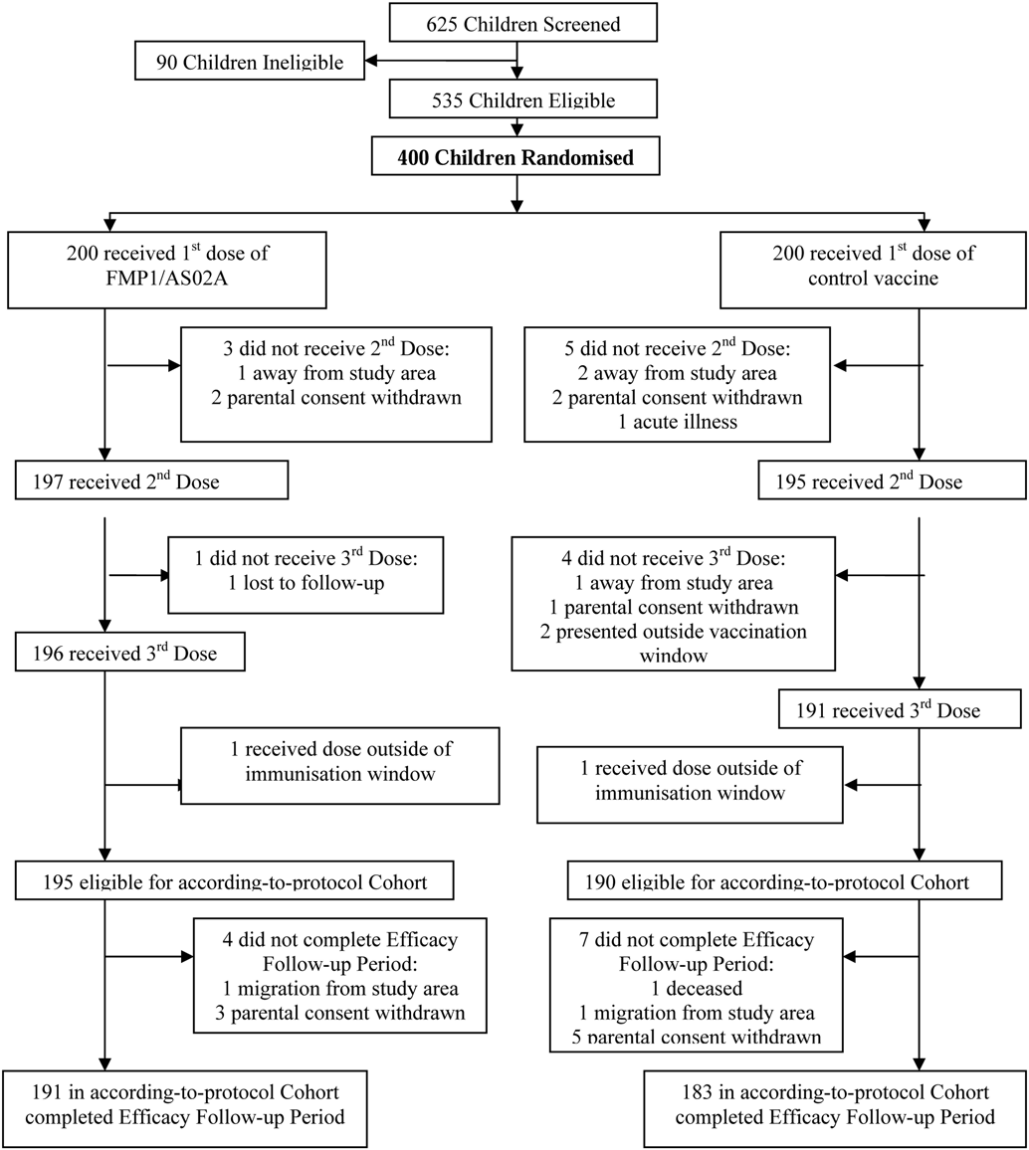
Trial profile.

A total of 625 children between 12 and 47 months of age were screened, 535 were deemed eligible, and 400 were randomised, 200 to each of the two vaccine groups (Figure 2). Compliance and retention rates were high: 385 of the 400 children (96%) completed the six months of follow-up and at least 95% of children attended each of the nine scheduled clinic visits.

### Recruitment

Study enrolment/randomisation, vaccination, and follow-up were conducted from March 2005 to December 2005.

### Baseline data

At baseline, the two groups were similar (Table 2) and within each field station (data not shown). The baseline clinical laboratory parameters were within normal range.

**Table 2:** Demographic, clinical, and laboratory baseline characteristics of 400 randomised children prior to first vaccination.

Characteristics	FMP1/AS02	Rabipur®
	N = 200	N = 200
Mean age in months±SD	29.6±10.1	30.0±9.9
No. male (%)	101 (51%)	118 (59%)
Mean height in cms±SD	84.8±8.0	85.1±8.3
Mean weight in kgs±SD	11.9±2.5	12.0±2.4
No. always using bednets (%)	142 (71%)	146 (73%)
No. with sickle cell trait (%)	36 (18%)	42 (21%)
No. G6PD deficient (%)	24 (12%)	25 (13%)
No. alpha-thalassemia homozygous (%)	22 (11%)	15 (8%)
No. alpha-thalassemia heterozygous (%)	80 (40%)	98 (49%)
No. parasitemic (%)	60 (30%)	56 (28%)
Median parasite density (75^th^ percentile)	0 (440)	0 (400)
Mean WBC ×10^3^/µL±SD	9.6±3.0	9.6±2.9
Mean Hb g/dL±SD	10.4±1.2	10.3±1.2
Mean platelets ×10^3^/µL±SD	326±121	324±112
Mean lymphocyte count ×10^3^/µL±SD	5.5±2.0	5.5±2.0
Mean creatinine Mm/L±SD	39.5±14.6	38.7±15.4
Mean ALT U/L±SD	12.4±8.6	13.3±11.0
GM anti-MSP-1_42_ antibody concentration (µg/mL)	1.34	1.53
Median anti-MSP-1_42_ antibody concentration (25^th^, 75^th^ percentiles)	1.6 (0.4, 7.7)	1.5 (0.4, 6.4)

ALT, alanine aminotransferase; G6PD, glucose-6-phosphate dehydrogenase deficiency; GM, Geometric mean; Hb, haemoglobin; SD, standard deviation; WBC, white blood cell.

### Numbers analyzed

A total of 374 (191 FMP1/AS02 and 183 in the comparator) children who completed the study as per the protocol were included in the according-to-protocol analysis.

### Outcomes estimation

#### Vaccine efficacy

**Figure 3:**
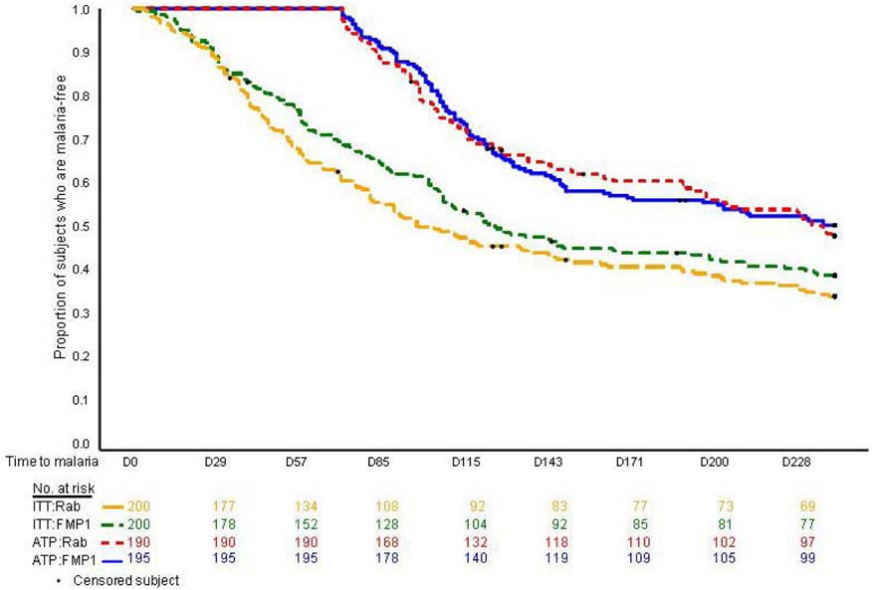
Kaplan-Meier curves for cumulative proportion with at least one episode of clinical malaria; both intention-to-treat and according-to-protocol cohorts are presented.

VE was determined for the 385 children who met all criteria for the primary efficacy analysis (Table 1); 195 children—98 in the *Rabipur®* group and 97 in the FMP1/AS02 group—experienced at least one case of *P. falciparum* malaria meeting the primary case definition. Estimated VE was 5.1% (95% CI: −26% to +28%; *p*-value = 0.7). The two groups had similar distributions of time to malaria following a third vaccination and were essentially identical throughout the period of observation (Figure 3). The according-to-protocol curves for the first 71 days, by definition, remain at 100% because cases occurring in this period do not contribute to the primary efficacy analysis.

The estimated VE for each secondary endpoint was similar to that of the primary (Table 1). Moreover, according-to-protocol analysis of monthly cross-sectional surveys during the efficacy follow-up period showed similar proportions of children with parasitaemia and similar distributions of parasite density in the two vaccine groups.

The rate of disease meeting the primary case definition per person year was 1.70 in the FMP1/AS02 group and 1.76 in the *Rabipur*® group (p-value for Poisson regression = 0.8). Thus, no significant difference between arms was observed with respect to the number of clinical episodes per subject meeting the primary case definition. The intention-to-treat analysis showed 122 and 131 cases in the FMP1/AS02 and comparator groups, respectively, for a VE of 15% (95% CI: −9% to 34%; *p*-value = 0.2). Active case detection identified 16% and 12% of the FMP1/AS02 and comparator groups, respectively. The comparable proportions in the according-to-protocol group were 23% and 28%.

### Ancillary analyses

We performed some exploratory, subgroup analyses that were not pre-specified. The following nominal p-values, not corrected for multiplicity, reflect the results of tests measuring whether the effect of vaccine differed by subgroup. At screening, parents were asked, “Does your child always sleep under a mosquito net at home?” VE in the two-thirds of the children (n = 288) reportedly always using bednets at baseline was −20% (*p* = 0.3) versus 48% (*p* = 0.017) in non-bednet users (interaction *p*-value = 0.009). VE did not differ as a function of age (*p* = 0.7); sex (*p* = 0.1); erythrocyte abnormalities (sickle cell trait, glucose 6-phosphate dehydrogenase deficiency (G6PD), or alpha-thalassemia; *p* = 0.7); presence of parasitaemia at baseline (*p* = 0.4); or anti-MSP-1_42_ antibody level either at baseline (*p* = 0.1) or at day 85 (*p* = 0.3).

We evaluated the possible impact of treatment with artemether/lumefantrine on the according-to-protocol time to clinical episodes before and after day 71 using several types of analyses. However, every analysis resulted in the same outcome, confirming to us the robustness of the results. Some of the exploratory analyses included: (i) time to first event starting at Day 0 (intention-to-treat) using various density cutoffs,(ii) time to first event starting at 14 days after Immnusation. 3 (according-to-protocol) using various density cutoffs, (iii) intention-to-treat and according-to-protocol analyses adjusted for time not at risk due to malaria treatment, and (iv) Poisson regression (intention-to-treat and according-to-protocol), where children were considered not at risk for 7 or 28 days following each new clinical case of malaria if they were treated with quinine or artemether/lumefantrine respectively.

In the summer of 2007, after all statistical analyses had been completed, we reviewed the distribution of parasite densities and found an unexpected bimodality. The distribution dipped between modes straddling the density range where microscopists switched from thick to thin film reading according to their standard operating procedures; the modes also straddled the primary efficacy endpoint cutoff, 50,000 parasites/µL.

We subsequently reread 643 pairs of films from all febrile episodes of malaria with parasitemia greater than 10,000 parasites/µL (618 pairs) and a random sample of episodes with densities less than 10,000 parasites/µL (25 pairs). For this rereading, each of two microscopists read both thin and thick films from the same malaria episode. If either of the pairs of thick or thin film readings yielded discordant results, a third, expert reader performed a tie-breaking read on both types of films.

The goal of the reread was to reevaluate our conclusions concerning the efficacy of the vaccine. Although the original reads performed during the course of the trial and the rereads performed in 2007 showed some discordance, and the point estimates of the various measures of efficacy changed modestly, the qualitative conclusions remained unchanged.

### Immunogenicity

**Figure 4:**
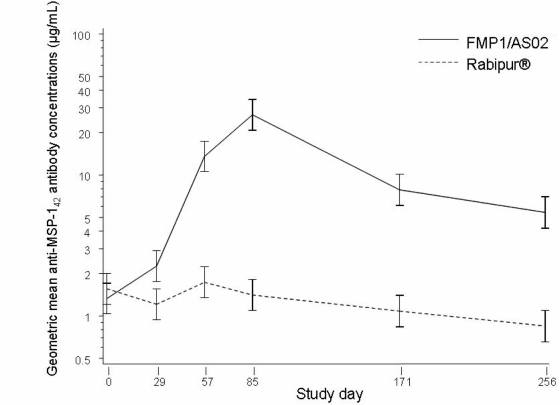
Geometric mean anti-MSP-1_42_ antibody concentrations estimated from the model, log_10_-transformed.

The concentration of MSP-1_42_-specific antibodies, as measured by ELISA (Figure 4), rose rapidly after each immunisation with FMP1/AS02, but declined slightly over time in the comparator group. Post-baseline geometric mean antibody concentrations were significantly different (*p*<0.0001). The highest concentration was observed on study day 85, approximately four weeks after third immunisations. At this time, the geometric mean antibody concentration in vaccine recipients was 26.3 µg/mL, compared with 1.3 µg/mL in the same group before vaccination, and 1.4 µg/mL in the comparator group at day 85 (*p*<0.0001). The antibody levels in vaccinees declined subsequently over time, but did not reach their prior baseline values during this follow-up period. Antibody response did not differ according to age when one-, two- and three-year-olds were compared by a longitudinal repeated measures analysis (*p* = 0.3). However, there was no association between peak (day 85) antibody response and time to first case of malaria (p = 0.33).

### Adverse events

Both FMP1/AS02 and the comparator vaccine had a good safety profile and were well tolerated. The FMP1/ AS02 group had more local (88% versus 8%) and vaccine-related, systemic (61% versus 16%) solicited reactions. More grade 3 AEs occurred in the FMP1/AS02 group; the majority were injection site pain or fever (Table 3) mostly resolving within 48 hours. In both groups, the frequency and severity of solicited AEs decreased with subsequent vaccinations (not shown).

**Table 3:** Number of children with at least one solicited adverse event up to seven days following any vaccination.

	Grade 3 AE			Any AE		
	FMP1/AS02	Rabipur®	p-value	FMP1/AS02	Rabipur®	p-value
	(n = 200)	(n = 200)		(n = 200)	(n = 200)	
All adverse events	33 (17)	7 (4)	<0.001	195 (98)	85 (43)	<0.001
Local (injection site)						
Pain	18 (9)	0 (0)	<0.001	175 (88)	14 (7)	<0.001
Swelling	3 (2)	0 (0)	0.25	80 (40)	2 (1)	<0.001
General (systemic)						
Fever	13 (7)	4 (2)	0.044	135 (68)	61 (31)	<0.001
Drowsiness	0 (0)	0 (0)	1.00	2 (1)	0 (0)	0.50
Loss of appetite	5 (3)	2 (1)	0.45	67 (34)	30 (15)	<0.001
Irritability/fussiness	0 (0)	1 (1)	1.00	26 (13)	2 (1)	<0.001

* Grade 3 AE = An adverse event that prevents normal, everyday activities: for pain = cries when limb is moved or spontaneously painful or limping or reluctance to bear weight; for swelling = >20 mm; for fever = >39.0°C. (No event was of higher severity than Grade 3.)

P-values are based on Fishers Exact Test.

In each group, 197 individuals experienced at least one unsolicited AE during the 30 days following vaccination. The most common, malaria, upper respiratory tract infection, skin disorders, and diarrhoea, were evenly distributed between the two vaccine groups.

Mean screening and monthly safety laboratory values (WBC, RBC, PLT, ALC, ALT, and Cr) were similar between the two groups and remained unchanged from baseline (not shown).

Mean haemoglobin values rose in both groups throughout the study (10.5 g/dL to 11.1 g/dL for FMP1/AS02; 10.3 g/dL to 10.9 g/dL for *Rabipur*®). Both groups included individuals who had episodic, transient low haemoglobin values (standard range: 6.5–13.0 g/dL) (nine individuals in FMP1/AS02; two individual in *Rabipur*® group) that returned to normal without transfusion. Additionally, one case of severe anaemia (Hb<5.0 g/dL) associated with severe malaria occurred in each vaccine group. In the intention-to-treat cohort, time to first episode of Hb<8.0 g/dL did not differ statistically between the groups (logrank p-value = 0.11; Hazard ratio = 1.53, 95% CI: 0.90–2.59).

### Serious adverse events

Serious and unexpected AEs were collected throughout the study. No reported SAE was judged related to vaccination. Twenty-six SAEs occurred in 23 children: 15 in the FMP1/AS02 group and 8 in the *Rabipur*® group. Fourteen of these SAEs were severe malaria cases, consistent with the 2000 World Health Organization criteria [27]. Nine of these severe malaria cases occurred in the FMP1/ AS02 group (cerebral malaria with anaemia<5.0 g/dL = 1, respiratory distress and prostration = 1, multiple convulsions = 1, parasitaemia≥20% = 3, respiratory distress = 3); five occurred in the *Rabipur*® group (anaemia<5.0 g/dL = 1, multiple convulsions = 1, parasitaemia≥20% = 2 instances in 1 subject, respiratory distress = 1).

A 15 month old male in the comparator group presented with malaria 24 days after his third vaccination, developed severe anaemia four days into antimalarial treatment, and died 38 days later. The child was hospitalised and transfused several times for a recurrent Coombs negative, haemolytic anaemia that was refractory to transfusion. No haemoglobinopathy was identified; however, three of his brothers had died of anaemia in early childhood. Autopsy found no specific aetiology.

## Discussion

### Interpretation

The FMP1/AS02 vaccine did not protect children living in Kombewa against first episodes of *P. falciparum* malaria; it did not reduce the overall incidences of clinical malaria episodes or of malaria infections, and did not reduce parasite densities (Table 1). The agreement between the according-to-protocol and the intention-to-treat efficacy analyses strengthens these findings. This corroborates the observation of no significant effect on clinical episodes in the combination B study [16].

The reasons for the lack of efficacy are unclear. The study had adequate power to detect VE of clinical importance. In malaria-naïve adults in the United States, FMP1/AS02 induced high titre, functional antibodies that recognised *P. falciparum* merozoites by indirect fluorescent antibody (IFA) and exhibited *in vitro* growth inhibitory activity [20]. Concurrent potency and stability testing of the final product showed no change over time. VE was not associated with age.

Active and passive case detection was intense and sustained, so that undetected cases of clinical malaria would be unlikely. It is also unlikely that the frequent active follow-up and aggressive treatment of malaria pursued as part of the study design prevented the detection of VE, because majority of the proportions of endpoint cases in both groups resulted from passive case detection alone (77% and 72% in FMP1/AS02 and *Rabipur*® recipients, respectively). The parasite reduction effect reported with MSP combination B vaccine candidate [16] as a possible surrogate for protection was not seen in our study.

Although FMP1/AS02 elicited a greater MSP-1_42_–specific immune response than did the comparator vaccine, this response was not a surrogate marker of protection. This is underscored by the results of the (albeit unscheduled and statistically insignificant) analysis for VE as a function of age; the youngest children, although mounting the highest antibody responses, showed no difference in protection from their older counterparts. Similarly, there was no association between peak (day 85) antibody response and time to first case of malaria (*p* = 0.33). The absence of relationship between MSP-1_42_–specific antibody levels and VE observed here contrasts with the positive correlation of anti-MSP-1_42_ antibodies with protection described in epidemiologic studies based on the Camp/FUP allele of MSP-1_42_ [28], [29].

The lack of observable VE has several hypothetical explanations. Owing to the high multiplicity of infection and the diversity of MSP-1_42_ phenotypes in Kenya (unpublished data), immunity conferred by the study vaccine may be directed to a minor parasite population. Additionally, the isotype [30], [31] or the sub-domain-specificity of the response may correlate best with protective immunity [12], [14]. Ongoing studies are investigating antibody fine-specificity. Of particular interest will be the investigation of the induction of antibodies that compete with specificities previously shown to inhibit parasite invasion [10], [32].

The possibility that efficacy is allele-specific is being investigated by genotyping of the parasites from breakthrough cases.

Protection against severe malaria is an important objective of malaria vaccine efforts; however, too few cases of severe malaria (according-to-protocol cohort: one case among FMP1/AS02 and two among comparator recipients; intention-to-treat cohort: nine and five cases, respectively) occurred in the present study to allow assessment of this.

The exploratory analysis suggesting that the vaccine might have an impact among children who do not use bednets is intriguing but requires further critical evaluation to determine its validity. In any case, it is clear that a malaria vaccine that targets only children who do not use bednets is an unsupportable strategy.

FMP1/AS02 had a good safety profile and, as expected, was more reactogenic than the comparator [20]–[23]. Despite this greater reactogenicity, dropout rates were low and similar in both groups; no dropout was attributed to an AE.

Haemoglobin levels were similar in both groups, rising throughout the study. Although more cases of low haemoglobin occurred in the FMP1/AS02 group, no case was judged causally related to vaccination and all resolved spontaneously. This observation led to an exploratory analysis to determine if there was a significant disproportionate drop in haemoglobin between groups. Fourteen children in the comparator group and 15 in the FMP1/AS02 group had an observed decrease from baseline of ≥3 grams of Hb at least once during the study. There was no significant difference in the risk of haemoglobin level decline to less than 8 g/dL, the cut-off value at enrolment. Thus, as in our previous studies [21]–[23], these data do not suggest a causal relationship between FMP1/AS02 and anaemia.

The MSP-1_42_ vaccine was clearly immunogenic, as evidenced by the rapid, high, and prolonged antibody response. There was a 20-fold increase from day 0 to day 85 in the FMP1/AS02 recipients, whereas the comparator group experienced a slight (11%) decrease during the same period. Although the antibody response waned after day 85, it remained well above both baseline and comparator for the duration of the follow-up period. Diminishment of malaria exposure during the dry season, or early case detection and treatment associated with the execution of the trial, might have contributed to the gradual decline in antibodies observed in the comparator group.

### Generalizability

The study population was chosen to represent the target population for a malaria vaccine: at risk, malaria-experienced children living in an area of intense malaria transmission. The primary objective of the trial being the efficacy of the vaccine, results should be broadly generalisable to children of the representative age bracket. As for results pertaining to the secondary objectives, safety and immunogenicity can be generalised to children of this age group exposed to *P. falciparum* infection in the study area as shown by a previous study [23], but may not be generalisable to children of other age groups or living under other transmission intensities.

### Overall evidence

In conclusion, this study provides no evidence that FMP1/AS02 protects against *P. falciparum* malaria in an area of holoendemic transmission, precisely where a malaria vaccine is most needed. Because of the clearly demonstrated overall lack of efficacy in this trial, FMP1/AS02 is no longer a promising candidate for further development as a monovalent malaria vaccine. No previous Phase IIb study of the FMP1 antigen has been conducted in the field and especially in an area of holoendemic malaria transmission. However, all the previous Phase I field studies evaluated the safety and immunogenicity and as with our data presented found FMP1/AS02 to be safe and highly immunogenic [21]–[23]. Despite the high immunogenicity and the holoendemic malaria transmission, there was no boosting effect noted in this study. We therefore propose that future MSP-1_42_ vaccine development efforts should focus on other antigen constructs and formulations.

## Supporting Information

Checklist S1 CONSORT Checklist
(0.04 MB DOC)

Protocol S1 Trial Protocol
(1.43 MB DOC)
