# Supplementary material for: Blood Stage Malaria Vaccine Eliciting High Antigen-Specific Antibody Concentrations Confers No Protection to Young Children in Western Kenya
Source: PLoS One. 2009 Mar 5;4(3):e4708. doi: 10.1371/journal.pone.0004708 (PMC2650803; doi:10.1371/journal.pone.0004708)
Supplement: Checklist S1 — CONSORT Checklist (0.04 MB DOC) [file pone.0004708.s001.doc]

625 Children Screened

535 Children Eligible

200 received 1^st^ dose of FMP1/AS02A

200 received 1^st^ dose of

control vaccine

197 received 2^nd^ Dose

195 eligible for ATP Cohort

191 in ATP Cohort completed Efficacy Follow-up Period

**400 Children Randomised**

90 Children Ineligible

195 received 2^nd^ Dose

196 received 3^rd^ Dose

191 received 3^rd^ Dose

3 did not receive 2^nd^ Dose:

1 away from study area

2 parental consent withdrawn

5 did not receive 2^nd^ Dose:

2 away from study area

2 parental consent withdrawn

1 acute illness

1 did not receive 3^rd^ Dose:

1 lost to follow-up

4 did not receive 3^rd^ Dose:

1 away from study area

1 parental consent withdrawn

2 presented outside vaccination

window

190 eligible for ATP Cohort

183 in ATP Cohort completed Efficacy Follow-up Period

1 received dose outside of immunisation window

4 did not complete Efficacy Follow-up Period:

1 migration from study area

3 parental consent withdrawn

7 did not complete Efficacy Follow-up Period:

1 deceased

1 migration from study area

5 parental consent withdrawn

1 received dose outside of immunisation window
