# Supplementary material for: Blood Stage Malaria Vaccine Eliciting High Antigen-Specific Antibody Concentrations Confers No Protection to Young Children in Western Kenya
Source: PLoS One. 2009 Mar 5;4(3):e4708. doi: 10.1371/journal.pone.0004708 (PMC2650803; doi:10.1371/journal.pone.0004708)
Supplement: Protocol S1 — Trial Protocol (1.43 MB DOC) [file pone.0004708.s002.doc]

**A Double-blind, Randomized, Controlled, Phase IIb Field Trial
in 12 to 47 Month-old Children in Western Kenya
to Evaluate the Efficacy, Safety and Immunogenicity
of the FMP1/AS02A Malaria Vaccine (WRAIR’s MSP-1 Antigen Adjuvanted in GlaxoSmithKline Biologicals’ AS02A)
Versus Rabies Vaccine**

IND No. 9202

WRAIR Protocol No. 1123

GSK Nomenclature eTrack study number 102010

eTrack abbreviated title MALARIA-036

KEMRI SSC No. 880

HSRRB Protocol No. A-13228

**Sponsor:** Department of the Army, Office of the Surgeon General

**Principal Investigator:** Bernhards R. Ogutu, MBChB, MMed (Peds), PhD

Kenya Medical Research Institute

US Army Medical Research Unit-Kenya

Box 54, Kisumu, Kenya

*Tel +254-57-22942*

*Fax +254-57-22903*

*Cell +254-733-812613*

*Email: bogutu@kisian.mimcom.net*

*STUDY REGULATORY MILESTONES*

Submitted to KEMRI CCR 19 May 04

Approved by KEMRI CCR 26 May 04

Submitted to PATH HSPC 2 Jun 04

Submitted to WRAIR ORM 2 Jun 04

Submitted to KEMRI SSC 28 Jun 04

Approved by KEMRI SSC 29 Jun 04

Submitted to KEMRI ERC 30 Jun 04

Submitted to WRAIR SRC 7 Jul 04

Approved by KEMRI ERC 13 Jul 04

Submitted to MRMC HSRRB 10 Nov 04

Submitted to US FDA 11 Nov 04

Approved by WRAIR SRC 1 Dec 04

Approved by MRMC HSRRB (Conditional) 8 Dec 04

Approved by PATH HSPC 1 Jan 05

End review period, US FDA 10 Dec 04

Approved by MRMC HSRRB (Final) 9 Feb 05

Begin recruitment 14 Feb 05

Begin screening 7 Mar 05

Study Day 0 (1st vaccination) 4 Apr 05

Table of Contents

[3 INVESTIGATORS and INSTITUTIONAL AFFILIATIONS 5](#__RefHeading___Toc112400608)

[4 SUMMARY 5](#__RefHeading___Toc112400609)

[3 INTRODUCTION AND BACKGROUND 5](#__RefHeading___Toc112400610)

[3.1 Life Cycle of the Malaria Parasite 5](#__RefHeading___Toc112400611)

[3.2 Status of Merozoite Stage Malaria Vaccines 5](#__RefHeading___Toc112400612)

[3.3 Clinical Experience with the AS02A Adjuvant 5](#__RefHeading___Toc112400613)

[3.3.1 Mal-015 and Mal-020 5](#__RefHeading___Toc112400614)

[3.3.2 Mal-025 5](#__RefHeading___Toc112400615)

[3.4 The FMP1 Antigen 5](#__RefHeading___Toc112400616)

[3.5 Experience with the FMP1/AS02A Malaria Vaccine 5](#__RefHeading___Toc112400617)

[3.5.1 Pre-clinical Trials 5](#__RefHeading___Toc112400618)

[3.5.2 Clinical Trials 5](#__RefHeading___Toc112400619)

[3.6 Justification of Dosing 5](#__RefHeading___Toc112400620)

[3.6.1 Justification of 0, 1, and 2 Month Dosing 5](#__RefHeading___Toc112400621)

[3.6.2 Justification of the 50 µg Dose 5](#__RefHeading___Toc112400622)

[3.7 Comparison Vaccine 5](#__RefHeading___Toc112400623)

[3.7.1 The Vaccine 5](#__RefHeading___Toc112400624)

[3.7.2 Vaccine Immunogenicity 5](#__RefHeading___Toc112400625)

[3.7.3 Vaccine Safety 5](#__RefHeading___Toc112400626)

[4 JUSTIFICATION 5](#__RefHeading___Toc112400627)

[4.1 Malaria in Western Kenya 5](#__RefHeading___Toc112400628)

[4.2 Target Population 5](#__RefHeading___Toc112400629)

[5 OBJECTIVES and HYPOTHESIS 5](#__RefHeading___Toc112400630)

[5.1 Primary 5](#__RefHeading___Toc112400631)

[5.2 Secondary 5](#__RefHeading___Toc112400632)

[5.3 Tertiary 5](#__RefHeading___Toc112400633)

[5.4 Hypothesis 5](#__RefHeading___Toc112400634)

[6 STUDY DESIGN and METHODOLOGY 5](#__RefHeading___Toc112400635)

[6.1 Overview 5](#__RefHeading___Toc112400636)

[6.2 Number of Subjects and Number of Study Arms 5](#__RefHeading___Toc112400637)

[6.3 Study Center 5](#__RefHeading___Toc112400638)

[6.3.1 The Walter Reed Project Kombewa Clinic 5](#__RefHeading___Toc112400639)

[6.3.2 Field Stations and Follow-up Centers in Kombewa Division 5](#__RefHeading___Toc112400640)

[6.3.3 The New Nyanza Provincial General Hospital, Kisumu 5](#__RefHeading___Toc112400641)

[6.4 Inclusion Criteria 5](#__RefHeading___Toc112400642)

[6.5 Exclusion Criteria 5](#__RefHeading___Toc112400643)

[6.6 Treatments Potentially Interfering with Vaccine-Induced Immunity 5](#__RefHeading___Toc112400644)

[6.7 Contraindications to Vaccination 5](#__RefHeading___Toc112400645)

[6.8 Indications for Deferral of Vaccination 5](#__RefHeading___Toc112400646)

[6.9 Safety Plan 5](#__RefHeading___Toc112400647)

[6.9.1 Criteria for Temporary Suspension of Vaccination 5](#__RefHeading___Toc112400648)

[6.9.2 Safety Review Prior to Administration of Consecutive Doses 5](#__RefHeading___Toc112400649)

[6.9.3 Procedure for Temporary Suspension of Vaccination 5](#__RefHeading___Toc112400650)

[6.9.4 Procedures for Resumption of Dosing Following Temporary Suspension of Vaccination 5](#__RefHeading___Toc112400651)

[6.9.5 Procedures for Permanent Discontinuation of Vaccination 5](#__RefHeading___Toc112400652)

[6.9.6 Dose Discontinuation in Individual Subjects 5](#__RefHeading___Toc112400653)

[6.9.7 Safety Precautions for Study Personnel 5](#__RefHeading___Toc112400654)

[6.10 Final and Addendum Efficacy and Safety Analyses and Reports 5](#__RefHeading___Toc112400655)

[7 CONDUCT OF THE STUDY 5](#__RefHeading___Toc112400656)

[7.1 General Study Aspects 5](#__RefHeading___Toc112400657)

[7.1.1 Screening and Enrollment Process 5](#__RefHeading___Toc112400658)

[7.1.2 Vaccination Process 5](#__RefHeading___Toc112400659)

[7.1.3 Post-immunization Evaluation Procedures 5](#__RefHeading___Toc112400660)

[7.1.4 Active and Passive Case Detection of Malaria 5](#__RefHeading___Toc112400661)

[7.1.5 Field Workers: Activities and Competencies 5](#__RefHeading___Toc112400662)

[7.1.6 Study Follow-up Periods 5](#__RefHeading___Toc112400663)

[7.2 Detailed Description of Study Stages and Visits 5](#__RefHeading___Toc112400664)

[7.3 Definition and Management of Symptomatic and Asymptomatic Malaria 5](#__RefHeading___Toc112400665)

[7.3.1 Definitions of Symptomatic and Asymptomatic Malaria for Research Purposes 5](#__RefHeading___Toc112400666)

[7.3.2 Definition of Symptomatic Malaria for Clinical Purposes 5](#__RefHeading___Toc112400667)

[7.3.3 Management of Symptomatic Malaria during the Study 5](#__RefHeading___Toc112400668)

[8 SAMPLE HANDLING and ANALYSIS 5](#__RefHeading___Toc112400669)

[8.1 Overview of Collection Time Points 5](#__RefHeading___Toc112400670)

[8.2 Handling of Biological Samples Collected by the Investigator 5](#__RefHeading___Toc112400671)

[8.2.1 Instructions for Handling of Serum Samples 5](#__RefHeading___Toc112400672)

[8.2.2 Labeling 5](#__RefHeading___Toc112400673)

[8.3 Laboratory Procedures 5](#__RefHeading___Toc112400674)

[8.3.1 Hematology 5](#__RefHeading___Toc112400675)

[8.3.2 Biochemistry 5](#__RefHeading___Toc112400676)

[8.3.3 Hemoglobinopathies 5](#__RefHeading___Toc112400677)

[8.3.4 Procedure for the Laboratory Diagnosis of Malaria 5](#__RefHeading___Toc112400678)

[8.3.5 Serology: Antibody Responses assayed with ELISAs 5](#__RefHeading___Toc112400679)

[8.3.6 Growth Inhibition Assay (GIA) 5](#__RefHeading___Toc112400680)

[8.3.7 Genotyping 5](#__RefHeading___Toc112400681)

[8.3.8 Optional Immunological Readouts 5](#__RefHeading___Toc112400682)

[8.3.9 Other Studies 5](#__RefHeading___Toc112400683)

[9 STUDY VACCINES and VACCINE ADMINISTRATION 5](#__RefHeading___Toc112400684)

[9.1 Study Vaccines 5](#__RefHeading___Toc112400685)

[9.1.1 FMP1/AS02A Vaccine 5](#__RefHeading___Toc112400686)

[9.1.2 Rabies Vaccine 5](#__RefHeading___Toc112400687)

[9.2 Vaccine Dosage and Administration 5](#__RefHeading___Toc112400688)

[9.2.1 Preparation of FMP1/AS02A Vaccine 5](#__RefHeading___Toc112400689)

[9.2.2 Reconstitution of Rabies vaccine 5](#__RefHeading___Toc112400690)

[9.2.3 Administration of Vaccines 5](#__RefHeading___Toc112400691)

[9.3 Vaccine Storage 5](#__RefHeading___Toc112400692)

[9.4 Randomization of Vaccine Allocation 5](#__RefHeading___Toc112400693)

[9.5 Method of Blinding and Breaking the Study Blind 5](#__RefHeading___Toc112400694)

[9.5.1 Blinding 5](#__RefHeading___Toc112400695)

[9.5.2 Breaking the Study Blind 5](#__RefHeading___Toc112400696)

[9.6 Replacement of Unusable Vaccine Doses 5](#__RefHeading___Toc112400697)

[9.7 Vaccine Accountability 5](#__RefHeading___Toc112400698)

[9.8 Concurrent Medication/Treatment 5](#__RefHeading___Toc112400699)

[9.8.1 Drugs for Treatment of Anaphylaxis 5](#__RefHeading___Toc112400700)

[9.8.2 Drugs to Treat Malaria 5](#__RefHeading___Toc112400701)

[10 ADVERSE EVENTS 5](#__RefHeading___Toc112400702)

[10.1 Eliciting and Documenting Adverse Events 5](#__RefHeading___Toc112400703)

[10.1.1 Definition of an Adverse Event 5](#__RefHeading___Toc112400704)

[10.1.2 Surveillance Period for Occurrence of Adverse Events 5](#__RefHeading___Toc112400705)

[10.1.3 Recording of Adverse Events 5](#__RefHeading___Toc112400706)

[10.1.4 Reporting of Adverse Events 5](#__RefHeading___Toc112400707)

[10.1.5 Solicited Adverse Events 5](#__RefHeading___Toc112400708)

[10.1.6 Unsolicited Adverse Events 5](#__RefHeading___Toc112400709)

[10.2 Assessment of Intensity 5](#__RefHeading___Toc112400710)

[10.3 Assessment of Causality 5](#__RefHeading___Toc112400711)

[10.4 Adverse Event Follow-up and Assessment of Outcome 5](#__RefHeading___Toc112400712)

[10.5 Serious Adverse Events 5](#__RefHeading___Toc112400713)

[10.5.1 Definition of a Serious Adverse Event 5](#__RefHeading___Toc112400714)

[10.5.2 Reporting Serious Adverse Events 5](#__RefHeading___Toc112400715)

[10.6 Treatment of Adverse Events 5](#__RefHeading___Toc112400716)

[11 SUBJECT COMPLETION and DROP-OUT 5](#__RefHeading___Toc112400717)

[11.1 Definition of a Drop-out 5](#__RefHeading___Toc112400718)

[11.2 Procedures for Handling Drop-outs 5](#__RefHeading___Toc112400719)

[11.3 Reasons for Drop-outs 5](#__RefHeading___Toc112400720)

[12 DATA MANAGEMENT and ANALYSIS 5](#__RefHeading___Toc112400721)

[12.1 Study Endpoints 5](#__RefHeading___Toc112400722)

[12.1.1 Primary Endpoint 5](#__RefHeading___Toc112400723)

[12.1.2 Secondary Endpoints 5](#__RefHeading___Toc112400724)

[12.1.3 Tertiary Endpoints 5](#__RefHeading___Toc112400725)

[12.2 Study Cohorts and Data Sets to be Evaluated 5](#__RefHeading___Toc112400726)

[12.2.1 Total Cohort 5](#__RefHeading___Toc112400727)

[12.2.2 Efficacy Cohort 5](#__RefHeading___Toc112400728)

[12.2.3 Safety Cohort 5](#__RefHeading___Toc112400729)

[12.2.4 Immunogenicity Cohort 5](#__RefHeading___Toc112400730)

[12.3 Sample Size 5](#__RefHeading___Toc112400731)

[12.4 Final analyses 5](#__RefHeading___Toc112400732)

[12.4.1 Analysis of Demographics 5](#__RefHeading___Toc112400733)

[12.4.2 Analysis of Test Article Efficacy 5](#__RefHeading___Toc112400734)

[12.4.3 Analysis of Immunogenicity 5](#__RefHeading___Toc112400735)

[12.4.4 Analysis of Safety 5](#__RefHeading___Toc112400736)

[12.4.5 Laboratory Parameters 5](#__RefHeading___Toc112400737)

[12.5 Administrative Matters 5](#__RefHeading___Toc112400738)

[13 ETHICAL CONSIDERATIONS 5](#__RefHeading___Toc112400739)

[13.1 Ethics and Regulatory Considerations 5](#__RefHeading___Toc112400740)

[13.1.1 Institutional Review Board/Ethics Review Committee (IRB/ERC) 5](#__RefHeading___Toc112400741)

[13.2 The Local Medical Monitor (Local Safety Monitor) 5](#__RefHeading___Toc112400742)

[13.3 The Data and Safety Monitoring Board (DSMB) 5](#__RefHeading___Toc112400743)

[13.3.1 Composition of the Board 5](#__RefHeading___Toc112400744)

[13.3.2 Role of the Board 5](#__RefHeading___Toc112400745)

[13.4 Risks and Potential Benefits to Subjects 5](#__RefHeading___Toc112400746)

[13.4.1 Vaccination 5](#__RefHeading___Toc112400747)

[13.4.2 Medical Treatment of Subjects 5](#__RefHeading___Toc112400748)

[13.4.3 Rabies Vaccination 5](#__RefHeading___Toc112400749)

[13.5 Precautions to Minimize Risk 5](#__RefHeading___Toc112400750)

[13.5.1 Vaccination 5](#__RefHeading___Toc112400751)

[13.5.2 Malaria Treatment During the Study 5](#__RefHeading___Toc112400752)

[13.6 Procedures for Maintaining Confidentiality 5](#__RefHeading___Toc112400753)

[14 REFERENCES 5](#__RefHeading___Toc112400754)

[15 STUDY BUDGET 5](#__RefHeading___Toc112400755)

[15.1 Study Budget Summary 5](#__RefHeading___Toc112400756)

[15.1 Budget Justification 5](#__RefHeading___Toc112400757)

[16 APPENDICES 5](#__RefHeading___Toc112400758)

[16.1 Appendix A: Study Personnel 5](#__RefHeading___Toc112400759)

[16.1.1 Roles of Study Personnel 5](#__RefHeading___Toc112400760)

[16.2 Appendix B: List of Study Specific Procedures (SSPs) 5](#__RefHeading___Toc112400761)

[16.3 Appendix C: List of Mal-036-Relevant Standard Operating Procedures (SOPs) 5](#__RefHeading___Toc112400762)

[16.3 Appendix D: Overview of Study Operational Scheme 5](#__RefHeading___Toc112400763)

[16.3.1 Operational Study Milestones 5](#__RefHeading___Toc112400764)

[16.3.2 Study Recruitment Plan 5](#__RefHeading___Toc112400765)

[16.3.3 Study Operational Field Plan 5](#__RefHeading___Toc112400766)

[16.4 Appendix E: Administrative Matters 5](#__RefHeading___Toc112400767)

[16.4.1 General Administrative Matters 5](#__RefHeading___Toc112400768)

[16.4.2 USAMRMC Specific Administrative Procedures 5](#__RefHeading___Toc112400769)

[16.6 Appendix F: World Health Organization Criteria for Severe Falciparum Malaria 5](#__RefHeading___Toc112400770)

[16.6.1 Severe Manifestations of P. falciparum malaria (WHO, 2000, pp S/1-2) 5](#__RefHeading___Toc112400771)

[16.6.2 EXTRACT: Definition of Severe Falciparum Malaria (WHO, 2000, pp S1/1-11) 5](#__RefHeading___Toc112400772)

[16.6.3 World Health Organization (2000). Severe falciparum malaria 5](#__RefHeading___Toc112400773)

List of Abbreviations

®: Registered

3D7: Clone of *Plasmodium falciparum*

Ab: Antibody

ACTG: AIDS Clinical Trials Group

AE: Adverse Event

AESDR: Addendum Efficacy and Safety Data Report (10 months of efficacy data)

AEFP: Addendum Efficacy Follow-up Period

ALC: Absolute lymphocyte count

ALT: Alanine aminotransferase

Anti R32LR: Anti-CSP repeat antibodies

AS02A: Adjuvant System 2 of GSK without thimerosal

AUC: Area under the curve

bac. virus: Baculovirus

CBC: Complete blood count

CHF: Congestive heart failure

CMI: Cell mediated immunity

CRA: Clinical Research Associate

CRF: Case Report Form

CRC: Clinical Research Coordinator

CCRC: Certified Clinical Research Coordinator

CCR: Centre for Clinical Research

CS gene: Circumsporozoite gene of *P. falciparum*

CSP: Circumsporozoite protein

CTL: Cytotoxic T lymphocyte

D1K: Anti-gamma interferon monoclonal antibody

DSMB: Data and Safety Monitoring Board

DMSO: Dimethyl sulphoxide

DNA: Deoxyribonucleic acid

EFP: Efficacy Follow-up Period (6 months of efficacy data)

EIR: Entomological Inoculation Rate

EGF: Epidermal Growth Factor

ELISA: Enzyme linked immunosorbent assay

Elispot: Enzyme linked immunospot

ERC: Ethical Review Committee

FDA: US Food and Drug Administration

FESDR: Final Efficacy and Safety Data Report

FFC: Field Follow-up Center

FMP1: Falciparum Merozoite Protein 1

FS: Field Station

FW: Field Worker

G6PD: Glucose-6-phosphate dehydrogenase

GIA: Growth Inhibition Assay

GSK: GlaxoSmithKline

GSK Biologicals: GlaxoSmithKline Biologicals

GMT: Geometric Mean Titer

HBsAg: Hepatitis B surface antigen

HDCV: Human Diploid Cell Vaccine

Hgb: Hemoglobin

HLA: Human leukocyte antigen

hpf: High power field

HSPC: Human Subjects Protection Committee (PATH IRB)

HSRRB: Human Subjects Research Review Board (MRMC)

HURC: Human Use Research Committee (WRAIR)

IATA: International Air Transport Association

IAW: In accordance with

ICH: International Conference on Harmonization

ID: Identification

IEC: Independent Ethics Committee

IFA: Indirect fluorescent antibody

IFN-gamma: Interferon gamma

IL‑2: Interleukin 2

IM: Intramuscular; intramuscularly

IRB: Institutional Review Board

ITT: Intention-to-treat

IU: International units

IU/l: International units per liter

IV: Intravenous; intravenously

kDa: kiloDalton

KEMRI: Kenya Medical Research Institute

KSDH: Kombewa Sub-District Hospital

Ksh: Kenya shillings

LDH: Lactate dehydrogenase

LMM: Local Medical Monitor

MBF: Malaria blood film

MHRA: Medicines and Healthcare products Regulatory Agency (UK)

ml: Milliliter

mmol/l: Millimoles per liter

MPL: Monophosphoryl Lipid A

MSP-1: Merozoite surface protein 1

MVI: Malaria Vaccine Initiative

NANP: Repeat epitopes of the circumsporozoite protein

NA: Not applicable

ND: Not done

NNPGH: New Nyanza Provincial General Hospital, Kisumu, Kenya

NR: Not related

o/a On or about

OD: Optical density

ORP: Office of Research Protections, USAMRMC

PATH: Program for Appropriate Technology in Health

*P. falciparum*: *Plasmodium falciparum*

PB: Probable

PBMC: Peripheral blood mononuclear cells

PCR: Polymerase chain reaction

PCV: Packed [red blood] cell volume

PO: Administered orally

POC: Point of contact

PPD: Pharmaceutical Product Development

QA: Quality Assurance

QC: Quality Control

QS21: *Quillaja saponaria* 21 (saponin derivative)

RAP: Reporting and Analysis Plan

RCO: Registered Clinical Officer

RIA Radioimmunoassay

RNAE: Reportable non-serious adverse event

RR: Relative risk

RTS,S: A candidate pre-blood stage malaria vaccine antigen [See next 2 entries]

RTS: Fusion protein consisting of a circumsporozoite protein-based antigen and the hepatitis B surface antigen (HBsAg)

S: 226 amino acid polypeptide corresponding to the surface antigen of hepatitis B virus (adw serotype)

SAE: Serious adverse event

SB Biologicals/SB Bio: SmithKline Beecham Biologicals

SB62: An oil-in-water emulsion

SBAS2: SmithKline Beecham Adjuvant System 2

SCI: Statistics Collaborative, Inc.

SD: Study Day (Study Day 0 = 1^st^ vaccinations)

Sf9: Insect cells derived from *Spodoptera frugiperda* (army worm)

SI: *Systemè* *International* [*d’Unité*]; International System [of

Units]

SID: Subject identification (number or card)

SOP: Standard Operating Procedure

SSC Scientific Steering Committee of KEMRI

SSP: Study Specific Procedures

SQ: Subcutaneous; subcutaneously

SU: Suspected

TBD: To be determined

TRAP: Thrombospondin-related anonymous protein

µg : Microgram

UL: Unlikely to be related

ul (or µl): Microliter

UAE: Unexpected Adverse Event

USAID: US Agency for International Development

USAMMDA: US Army Medical Materiel Development Activity

USAMRMC: US Army Medical Research and Materiel Command

USAMRU-K: US Army Medical Research Unit-Kenya

USD: United States dollars

Vacc: Vaccination

WB: Western Blot assay

WRAIR: Walter Reed Army Institute of Research, Silver Spring,

DC, USA

WRAMC: Walter Reed Army Medical Center, Washington, DC, USA

WRP: Walter Reed Project (USAMRU-K, local name)

WRP KC: WRP Kombewa Clinic

Glossary of Terms

**Blinding, Full**  Fully blind means that from Study Day 0 to 240, the only persons with access to individual study arm assignments will be (a) the two on-site Vaccine Preparation Teams consisting of the study pharmacist, pharmacist assistants, and drug manager, and (b) the statisticians preparing the randomization schedule who will not be performing statistical analysis until the study is unblinded.

**Blinding, Partial** Kenya site investigators and parents/subjects will remain blinded from Study Day 0 until full study unblinding. During the partially blinded period (from SD 240 to full unblinding), the study statisticians will be unblinded to individual and group study arm assignments so they can analyze the EFP database. (With release of the FESDR, study partners, including all investigators, will be unblinded to study results.)

**Clinician** A physician or clinical officer (physician assistant)

**Comparator** Rabies vaccine

**Drug Manager** A pharmacist, nurse, clinician, or research coordinator responsible for ensuring proper storage and accountability of test and comparator vaccines

**Eligible** In complete accord with all inclusion and exclusion criteria and, thus, qualified for enrollment into the study

**Enrolled** Eligible and having received at least one vaccine dose

**Evaluable** Eligible and having complied with all procedures defined in the protocol (see Section 12 for specific criteria)

**Local Medical** A medically qualified, host-country individual responsible

**Monitor** for actions with regard to the ethics and clinical safety of the study, especially the proper assessment of adverse events

**Parent/guardian** The biological parent or legal guardian granting informed parental consent for a child to enroll in the study

**Participant** Either a subject or a parent/guardian

**Sponsor** The study sponsor is the Office of the Surgeon General (OTSG), Department of the Army; OTSG authority devolves to the Office of Research Protections, US Army Medical Research and Materiel Command (USAMRMC-ORP); ORP in turn monitors regulatory compliance via the US Army Medical Materiel Development Activity (USAMMDA; Point of Contact: Dr. Moshe Shmuklarsky) and its human subject research is authorized by its Human Subjects Research Review Board (HSRRB; Point of Contact: Ms. Caryn Duchesneau, Vice Acting Chair)

**Study Auditor** An individual responsible for auditing the work of the Study Monitor(s)

**Study Monitor** An individual responsible for assuring proper conduct of a clinical study at one or more investigational sites

**Subject**  A child enrolled in the study

**Test article**  The FMP1/AS02A candidate malaria vaccine (The WRAIR’s MSP-1 antigen adjuvanted in GSK Biologicals’ AS02A)

**Title:**

A Double-blind, Randomized, Controlled, Phase IIb Field Trial in 12 to 47 Month-old Children in Western Kenya to Evaluate the Efficacy, Safety and Immunogenicity of FMP1/AS02A Malaria Vaccine (WRAIR’s MSP-1 Antigen Adjuvanted in GlaxoSmithKline Biologicals’ AS02A) Versus Rabies Vaccine

**Running Title** MSP-1 Phase IIb in Children

**IND No.** 9202

**WRAIR Protocol No** 1123

**GSK Nomenclature** eTrack study number: 102010

eTrack abbreviated title: MALARIA-036

**HSRRB Protocol No.** A-13228

**Sponsor** Department of the Army, Office of the Surgeon General

# INVESTIGATORS and INSTITUTIONAL AFFILIATIONS

**Principal Investigator** Bernhards R. Ogutu, MBChB, MMed (Peds), PhD

Kenya Medical Research Institute

US Army Medical Research Unit-Kenya

Box 54, Kisumu, Kenya

*Tel +254-57-22942*

*Fax +254-57-22903*

*Cell +254-733-812613*

*Email: bogutu@kisian.mimcom.net*

**Associate Investigators**

***KEMRI*** Mark R. Withers, LTC (P), MC, FS

Mark E. Polhemus, LTC, MC

Lucas Otieno, MBChB

Shon A. Remich, MAJ, MC

John N. Waitumbi, DVM, PhD

Denise A. McKinney, RN, CCRC, CCRA

Box 54, Kisumu, Kenya

*Tel +254-57-22942*

*Fax +254-57-22903*

***WRAIR*** D. Gray Heppner, COL, MC

Christian Ockenhouse, LTC, MC

Department of Immunology, WRAIR Silver Spring, Maryland 20910 USA

*Tel +301-319-9414*

**Study Contributors**

***KEMRI*** Ezekiel Oenga, BPharm

Joram Siangla, MSc

Willis Okoth, BSc

Raphael Pundo, MSc

Box 54, Kisumu, Kenya

*Tel +254-57-22942*

*Fax +254-57-22903*

***WRAIR*** Jeff Lyon, PhD

Evelina Angov, PhD

David Haynes, PhD

Carolyn Holland, MPH

Department of Immunology, WRAIR

*Tel +301-319-9414*

*Fax +301-319-7358*

***GSK Biologicals*** Amanda Leach, MSc, MRCPCH

Clinical Development Manager

*Tel +32-2-656-7788*

*Fax +32-2-656-6160*

Isabelle Ramboer, PhD

Central Study Coordinator, Malaria projects

*Tel +32-2-656-6820*

Fax +32-2-656-8044

***Malaria Vaccine Initiative*** Elissa Malkin, DO, MPH

Senior Medical Officer

The PATH Malaria Vaccine Initiative

Tel: +240-395-2711

Fax: +240-395-2591

***US Agency for International***  Carter Diggs, MD, PhD

***Development*** Senior Technical Advisor

*Tel +202-712-5728*

*Fax +202-216-3702*

Lorraine A. Soisson, PhD

Technical Advisor

USAID Malaria Vaccine Development Program

6 Van Fleet Court

Hillsborough, NJ 08844 USA

*Tel +908-369-7704*

*Fax +908-369-7724*

***Clinical Research*** Odika J. Apollo, RCO

***Coordinators*** Melanie Onyango, BSc, CCRC

Stacy M. Gondi, RCO

WRP/KEMRI

Box 54, Kisumu, Kenya

*Tel +254-57-22942*

*Fax +254-57-22903*

***Local Medical Monitors*** Juliana Otieno, MBChB, MMed, Peds

Chief, Dept of Pediatrics

New Nyanza Provincial General Hospital

PO Box 849, Kisumu, Kenya

*Cell +254-733-715917*

*Fax +254-57-41330*

*Alternate*:

James Gesami, MBChB, MMed

Nyanza Provincial Medical Headquarters

PO Box 721, Kisumu, Kenya

*Tel +254-57-40091/41550*

*Fax +254-57-21870*

*Cell +254-733-826090*

***Statistical Consultants*** Janet Wittes, PhD

Kathryn Tucker, MS

Wasima Rida, PhD

Statistics Collaborative, Inc.

1710 Rhode Island Ave, NW; Suite 200

Washington, DC 20036 USA

*Tel +202-429-9267*

*Fax +202-429-9268*

***Study Monitors*** Ms. Yolanda Lewis

Ms. Charleen Du Plessis

Pharmaceutical Product Development

Isabelle Ramboer, PhD

GlaxoSmithKline Biologicals

***Study Auditor*** Ms Shirley Roach, RN

US Army Medical Materiel Development Activity

***IND Sponsor*** Department of the Army, Office of the Surgeon General

***Funding*** ***Sources*** Malaria Vaccine Initiative, PATH

US Agency for International Development

***IRBs/ERCs*** Kenya Medical Research Institute

Ethical Review Committee

Mbagathi Road

PO Box 54840

Nairobi, Kenya

Human Subjects Protection Committee

Program for Appropriate Technology in Health

1455 NW Leary Way

Seattle, WA 98107-5136 USA

Office of Research Protections

Human Subjects Protection Division

USAMRMC

Ft. Detrick, MD USA

# SUMMARY

| **Title** | A Double-blind, Randomized, Controlled Phase IIb Field Trial in 12 to 47 Month-old Children in Western Kenya to Evaluate the Efficacy, Safety, and Immunogenicity of the FMP1/AS02A Malaria Vaccine (WRAIR’s MSP-1 Malaria Antigen Adjuvanted in GlaxoSmithKline Biologicals’ AS02A) Versus Rabies Vaccine |  |
| --- | --- | --- |
| **Study Population** | Healthy 12- to 47-month-old, malaria-exposed children |  |
| **Study Site** | The Walter Reed Project Kombewa Clinic in Kombewa Division, Kisumu District, Nyanza Province, western Kenya (and 12 small field stations within Kombewa Division) |  |
| **Objectives** | ***Primary***   - To assess the efficacy of the FMP1/AS02A malaria vaccine on clinical episodes of *P. falciparum* malaria during the Efficacy Follow-up Period (EFP)   ***Secondary (Efficacy)***   - To assess the efficacy of the FMP1/AS02A malaria vaccine against clinical episodes of *P. falciparum* malaria of differing severity during the EFP   ***Secondary (Safety and Immunogenicity)***   - To assess solicited adverse events occurring in the seven days following each dose of vaccine - To assess unsolicited adverse events occurring in the 30 days following each dose of vaccine - To assess serious adverse events occurring after the first dose of vaccine to the end of the EFP - To assess the immunogenicity of the FMP1/AS02A malaria vaccine from the first dose of vaccine to the end of the EFP - To assess the association between MSP-1 antibody titers and the subsequent risk of clinical malaria during the EFP   ***Tertiary (Safety and Immunogenicity)***   - To assess the safety of the FMP1/AS02A malaria vaccine during the Addendum Efficacy Follow-up Period (AEFP) - To assess the immunogenicity of the FMP1/AS02A malaria vaccine during the AEFP   ***Tertiary (Efficacy and Exploratory)***   - To assess the efficacy of the FMP1/AS02A malaria vaccine against clinical episodes of *P. falciparum* malaria during the AEFP - To assess the biological effects of the FMP1/AS02A malaria vaccine during the AEFP |  |
|  |  |  |
| **Test Vaccine** | 50 μg of lyophilized FMP1 antigen reconstituted with 0.50ml of AS02A adjuvant |  |
| **Comparator Vaccine** | Rabies vaccine |  |
| Immunization Schedule | 0, 1, and 2 months (Provisionally: Study Days 0, 29, and 57) |  |
| Route of Administration | IM injection into left anterolateral thigh muscle |  |
| Number of Subjects | Four hundred (400) subjects of either gender will be enrolled and randomized 1:1 to FMP1/AS02A or rabies vaccine |  |
| Study Duration | Enrollment is expected to be completed within 7 to 10 days; however, it may continue for up to an additional 15 (for a total of 25 days) if the desired number of subjects has not been enrolled by the end of the 10^th^ vaccination day. Once enrolled, each subject will be followed for 12 months. Total study duration will be about 14 months. |  |
| **Primary endpoint** | Measurement of clinical efficacy of the vaccine, during the efficacy follow-up period (EFP), as determined by time to first clinical episode of *P. falciparum* malaria meeting the primary case definition |  |
| **Secondary endpoints** | Measurements of efficacy during the EFP:   - Times to clinical episode of *P. falciparum* malaria meeting the various secondary case definitions shown in Exhibit 8 - Time to presence of asexual stage *P. falciparum* parasites during the six-month efficacy follow-up period - Time to parasite density ≥ 50,000 asexual stage *P. falciparum* parasites per microliter of blood during the six-month efficacy follow-up period - Number of clinical episodes of malaria, per subject, meeting the primary case definition - Presence of MSP-1 3D7 parasites in clinical cases of malaria meeting the primary case definition - Presence of MSP-1 3D7 parasites at a given cross-section survey time - Time to anemia defined as Hgb < 8.0 g/dl, whether by active or passive case detection - Proportion of subjects with anemia (Hgb < 8.0 g/dl)   Safety during the EFP:   - Occurrences of solicited AEs during a seven‑day follow-up period (vaccination day and six subsequent days) after each vaccination (five visits: vaccination day and days 1, 2, 3, and 6) - Occurrences of unsolicited AEs during a 30-day follow-up period after each vaccination (vaccination day and 29 subsequent days) - Occurrences of SAEs between the first vaccination and the end of the EFP   Immunogenicity during the EFP:   - Geometric means of MSP-1 Ab titers at Visits 2, 7, 12, 17, 20, and 23 (o/a Study Days 0, 29, 57, 85, 171, and 256) |  |
| **Tertiary Endpoints** | Measurement of safety (during the EFP and Addendum Efficacy Follow-up Period [AEFP]):   - Occurrences of SAEs between the first vaccination and the end of the study   Measurement of immunogenicity (during the EFP and AEFP):   - Geometric means of MSP-1 Ab titers at Study Days 0, 29, 57, 85, 171, 256, and 340   Measurement of efficacy and other biological effects by either active or passive detection (during the AEFP unless otherwise noted):   - Times to first clinical episode of *P. falciparum* malaria meeting the primary case definition (EFP and AEFP combined) - Times to first malaria episode of *P. falciparum* malaria meeting the secondary case definitions in Exhibit 8 (EFP and AEFP combined) - Time to presence of asexual stage *P. falciparum* parasites (EFP and AEFP combined) - Time to parasite density ≥ 50,000 asexual stage *P. falciparum* parasites per microliter of blood (EFP and AEFP combined) - Number of clinical *P. falciparum* malaria episodes, per subject, meeting the primary case definition (EFP and AEFP combined) - Presence of MSP-1 3D7 parasites in clinical cases of malaria meeting the primary case definition (EFP and AEFP combined) - Presence of MSP-1 3D7 parasites at a given cross-section survey time (EFP and AEFP combined) - Vaccine-induced GIA activity - The parasite density at each episode of malaria infection meeting the primary and secondary definitions of clinical malaria and infection (see Exhibit 8) - *P. falciparum* malaria allele types per malaria episode, meeting the primary and secondary definitions of clinical malaria and infection (see Exhibit 8) - Times to first observation of anemia (Hgb < 8.0 g/dl), whether by active or passive case detection (EFP and AEFP combined) - Hgb levels at all scheduled points of collection - Proportion of subjects with anemia (Hgb < 8.0 g/dl) at all scheduled points of hgb collection - Proportion of subjects with a documented decline in Hgb level of ≥ 20% - Proportion of subjects with anemia (Hgb < 8.0 g/dl) at last scheduled study hgb collection - Number of cases of documented decline in Hgb level from baseline to last study sample collection - Parasite density at all scheduled points of collection - Non-3D7 parasite densities at all scheduled points of collection - Genotyping for multiplicity of infections - Antibody against fragments of MSP-1_42_ - Antibody able to compete for binding to recombinant MSP-1_42_ - Antibody able to inhibit secondary processing of MSP-1_42_ |  |

# INTRODUCTION AND BACKGROUND

## Life Cycle of the Malaria Parasite

The malaria parasite has a complex life cycle. The bite of an infectious mosquito transmits the sporozoite form of the parasite to humans. These sporozoites travel through the bloodstream and ultimately invade liver cells where they multiply asexually as exoerythrocytic stage parasites. The exoerythrocytic forms of *Plasmodium falciparum* mature in five to seven days releasing thousands of tissue merozoites that invade erythrocytes and initiate the erythrocytic phase of the infection. The free merozoites invade erythrocytes, undergo asexual maturation, and ultimately rupture the erythrocytes releasing new merozoites. This phase, characterized by cyclic destruction of erythrocytes, results in the clinical disease known as malaria. A small number of the invading merozoites do not multiply, but instead differentiate into sexual forms known as gametocytes. When ingested by a female mosquito, male and female gametocytes unite to form a zygote, which then matures to release sporozoites which subsequently migrate to mosquito salivary glands where they are available to infect the next person bitten, thus completing the parasite’s life cycle.

## Status of Merozoite Stage Malaria Vaccines

Malaria is a major worldwide problem with transmission occurring throughout Africa, Asia, Oceania, and Latin America. Over two billion people are estimated to live in malarious areas of the world. In Africa, for example, an estimated 300 to 500 million cases of malaria occur each year (1). Between 700,000 and 2.7 million people die from malaria each year; the majority are African children (2, 3). The sub-Saharan African region accounts for 90% of the disease burden which affects mainly children below five years old. About 75% of the 550 million people in Africa live in areas of highly endemic stable transmission. Another 18% live in epidemic‑prone areas where malaria transmission is seasonal and unstable, with all age groups being vulnerable to infection and disease (4). Between 400 and 900 million acute febrile episodes occur yearly in African children under five years of age living in endemic areas. Although about half of these children are not parasitemic, all merit consideration for malaria-specific therapy. Fewer than 20% of febrile episodes and deaths come to the attention of any formal health system. The relatively few ill patients who have any contact with the health services represent the “ears of the hippopotamus” (2); the majority of the patients receive minimal or no medical attention (4). *P. falciparum* causes about 80% of the infections and practically all the severe forms of malaria and deaths.

The objective of developing an effective malaria vaccine has been a major focus of malaria research for many years. This task has proven more difficult than initially thought. To date, no highly effective malaria vaccine is on the horizon in spite of considerable progress. Several lines of evidence show that blood stage immunity is both important and achievable. Adults living in areas of high malaria transmission become relatively resistant to malaria while continuing to become infected, suggesting that the immune response against antigens expressed during the blood stage may play a role. This has been corroborated by experiments where serum has been transfused from heavily exposed individuals to ill patients and the disease has been modulated. Moreover, the feasibility of impacting the parasite with a recombinant protein vaccine has been demonstrated in recent field trials in Papua New Guinea that showed parasite density was decreased in a vaccinated group (5).

Two critical steps of the malaria life cycle have been closely examined as potential targets for vaccine-induced immunity: 1) the invasion of hepatocytes by sporozoites and 2) the invasion of erythrocytes by merozoites. The merozoite stage of the parasite, like the sporozoite, is a logical target for a malaria vaccine because blockade of erythrocyte invasion would completely prevent clinical disease. Even if the vaccine were incapable of completely blocking merozoites from invading red blood cells, an immunologic reduction in the magnitude of parasitemia may significantly impact disease. Therefore, the identification of the molecular mechanisms of merozoite invasion of red blood cells has been an active area of investigation in the field of malaria vaccine research. Several antigens have been identified that have the potential to inhibit merozoite invasion of red cells. The most studied of these antigens, and also the most promising candidate as a constituent of a blood stage vaccine, is the merozoite surface protein 1 (MSP-1), a 195 kDa antigen found on the surface of merozoites. MSP-1 undergoes processing by proteolytic cleavage to a 42 kDa fragment and further to a 19 kDa fragment that has been implicated in the invasion of erythrocytes by the merozoite (6).

Several lines of evidence point to MSP-1 as a promising vaccine candidate. Antibodies directed against portions of MSP-1, in particular against the 19 kDa C-terminal fragment, inhibit erythrocyte invasion (6, 7). At least one field study has demonstrated an association between the presence of antibodies against MSP-1 and resistance to clinical malaria (8). Immunization with recombinant fragments of this molecule also protects non-human primates against *P. falciparum* (9) when used with Complete Freund’s Adjuvant, and mice against *P. yoelii* (10, 11). Passive transfer of immune sera in mice also confers protection (12). Although the weight of evidence indicates that antibodies against the C-terminal fragment of MSP-1 are protective, in one case immunization with C-terminal constructs did not result in protection (13). Lack of protection in some cases could be due to the use of antigenic constructs that lack a proper conformational structure because this region of MSP-1 is known to be conformationally dependent. In addition, vaccine efficacy is critically dependent on vaccine formulation. MSP-1 may also be the target of CD4^+^ T cells. Several T-cell epitopes have been identified (14, 15).

A recombinant version of the 42 kDa C-terminal portion of MSP-1 has been produced at the Walter Reed Army Institute of Research. This falciparum merozoite protein 1 (FMP1) is produced as a histidine-tagged (His6) fusion protein in *Escherichia (E.) coli*. The antigen, derived from the 3D7 clone of *P. falciparum*, contains both T-cell and B-cell epitopes. Monoclonal antibodies raised against native parasite MSP-1 recognize correctly folded conformational disulfide-bonded epitopes within the recombinant 42 kDa antigen. An efficient fermentation and purification process has been developed to produce this antigen on a scale compatible with industrial manufacture. The vaccine is formulated in the same adjuvant system used in the RTS,S vaccine, now called AS02A (the second ‘A’ denotes absence of thimerosal). To increase product stability, the MSP-1_42_ antigen is manufactured as a lyophilized product and reconstituted just prior to injection.

As background, the “RTS,S” antigen is a recombinant protein based upon the *P. falciparum* CS protein covalently linked to the hepatitis B surface antigen. RTS,S when formulated with the adjuvant system AS02A, is a prototype malaria vaccine now undergoing field trials. Due to the more extensive experience with RTS,S/AS02A in children in malaria endemic populations, these data are summarized in the protocol as supporting evidence for the safety of a FMP1/AS02A formulation in children in Kenya.

The study proposed here is a Phase IIb efficacy study of FMP1/AS02A in a malaria‑endemic area of western Kenya. If this candidate vaccine proves efficacious and safe, further studies may be pursued with this same formulation to assess its efficacy singly or in combination with a pre-blood-stage antigen. Several trials have studied the antigen FMP1 and the adjuvant AS02A (see Exhibit 1).

Exhibit 1. Summary of clinical trials of FMP1 and AS02A

| **Study** | **Vaccine** | **Study population** | **Vaccine groups** | **Design (and results)** |
| --- | --- | --- | --- | --- |
| Mal-014 | FMP1/AS02A | 15 malaria-naïve adults at Walter Reed | 0.10, 0.25, 0.50 µg doses | Phase Ia open-label, dose-escalation. 0, 1, 3 month schedule |
| Mal-015 | RTS,S/AS02A | 6-11 year-old children in the Gambia | 0.10, 0.25, 0.50 µg doses  on a 0, 1 and 3 month schedule | Phase Ib trial |
| Mal-019 | FMP1/AS02A and RTS,S/AS02A | 71 malaria-naïve adults at Walter Reed | Four vaccine groups, 11 in challenge control: - FMP1/AS02A+RTS,S/AS02A at   separate sites of injection (n=15)  - FMP1/AS02A+RTS,S/AS02A at   one injection site (n=15)  - FMP1/AS02A (n=15)  - RTS,S/AS02A (n=15)  - Unvaccinated controls (n=11) | Phase I/IIa challenge study (Showed no interference between RTS,S/AS02A and FMP1; FMP1 alone did not prevent malaria) |
| Mal-020 | RTS,S/AS02A | 1-5 year-old children in the Gambia | 0.10, 0.25, 0.50 µg doses  on a 0, 1 and 3 month schedule | Phase Ib trial |
| Mal-024 | FMP1/AS02A vs. *Imovax®* Rabies | 40 malaria-exposed adults in western Kenya | Two study groups: - Full dose (0.50 µg) of FMP1/AS02A (n=20) - Imovax® Rabies (n=20) | Phase Ib to assess safety and immunogenicity. 0, 1, 2 month schedule (Vaccine deemed safe to perform Phase Ib trial in children) |
| Mal-025 | RTS,S/AS02A vs. *Engerix-B* (Hep B) | Sixty 1-4 year-old children in Mozambique | 0.25mL | Phase Ib safety & immunogenicity trial. 0, 1, 2 month schedule |
| Mal-031 | FMP1/AS02A vs. *Imovax®* Rabies | 135 1-4 year-old malaria-exposed children in western Kenya | Four study groups: - One-fifth dose (0.10 µg) of FMP1/AS02A (n=30);  - Half dose (0.25 µg) of FMP1/AS02A (n=30); - Full dose (0.50 µg) of FMP1/AS02A (n=30); - Imovax® Rabies (n=45). | Phase Ib trial to assess safety and immunogenicity and to decide on dose level for Mal-036. 0, 1, 2 month schedule  (Vaccine deemed safe to perform Phase IIb trial in children) |

## Clinical Experience with the AS02A Adjuvant

Clinical development of the RTS,S/AS02A vaccine provides the most extensive experience of the use of the AS02A adjuvant in children to date. This section, along with Exhibit 1 above, summarizes the aspects of those studies most relevant to the design of the current protocol.

### Mal-015 and Mal-020

Two phase I safety and immunogenicity studies evaluating 0.1 ml, 0.25 ml, and 0.5 ml doses of RTS,S/AS02A have been conducted in Gambian children. Mal-015 studied children aged 6 to 11 years, while Mal-020 studied children aged 1 to 5. A 0.5 ml dose of RTS,S/AS02A contains 50 µg RTS,S, 50 µg MPL®, 50 µg QS21, and 250 µl SB62. Each study used *Imovax®* human diploid cell rabies vaccine (Aventis Pasteur, Lyon) as the control. One purpose of these studies was to aid in the selection of the dose level in future pediatric development of the RTS,S/AS02A candidate vaccine.

Exhibit 2 displays the solicited symptoms reported in Mal-015 and Mal-020. Pain at the injection site occurred frequently across all study groups. The incidence of swelling increased with dose level. Most general solicited symptoms were mild to moderate in intensity and short lasting. Grade 3 general solicited symptoms were infrequent in both studies; the symptoms resolved or decreased in intensity within 24 hours.

In children aged 6 to 11 years (Mal-015), headache was the most frequently occurring general solicited symptom in subjects receiving RTS,S/AS02A at any dose level.

In the children aged 1 to 5 years (Mal-020), fever was the most frequently occurring general symptom in subjects receiving 0.1 ml dose RTS,S/AS02A. In subjects receiving 0.25 ml dose RTS,S/AS02A, loss of appetite was the most frequent symptom reported. In subjects receiving 0.5 ml dose of RTS,S/AS02A, irritability/fussiness was the most frequently reported symptom, occurring after 27% of doses, compared to 12% of doses in the control group.

Exhibit 2. Frequency of solicited symptoms per dose during the 4-day follow-up period for each vaccine group

|  | | **RTS,S/ AS02A**  **(0.1 ml)** | | **Imovax®** | | **RTS,S/ AS02A**  **(0.25 ml)** | | **Imovax®** | | **RTS,S/ AS02A**  **(0.5 ml)** | | **Imovax®** | |
| --- | --- | --- | --- | --- | --- | --- | --- | --- | --- | --- | --- | --- | --- |
| Mal-015: ages 6-11 years | | N=60 | | N=30 | | N=59 | | N=30 | | N=60 | | N=30 | |
| Symptom |  | n | % | n | % | n | % | n | % | n | % | n | % |
| Injection site pain | Any | 53 | 88.3 | 21 | 70.0 | 53 | 89.8 | 27 | 90.0 | 59 | 98.3 | 26 | 86.7 |
|  | G3 | 0 | 0.0 | 0 | 0.0 | 4 | 6.8 | 0 | 0.0 | 8 | 13.3 | 0 | 0.0 |
| Injection site swelling | Any | 5 | 8.3 | 1 | 3.3 | 10 | 16.9 | 0 | 0.0 | 12 | 20.0 | 0 | 0.0 |
|  | G3 | 1 | 1.7 | 0 | 0.0 | 1 | 1.7 | 0 | 0.0 | 4 | 6.7 | 0 | 0.0 |
| Limited arm motion | Any | 5 | 8.3 | 1 | 3.3 | 12 | 20.3 | 2 | 6.7 | 11 | 18.3 | 0 | 0.0 |
|  | G3 | 0 | 0.0 | 0 | 0.0 | 2 | 3.4 | 0 | 0.0 | 2 | 3.3 | 0 | 0.0 |
| Fever | Any* | 6 | 10.0 | 0 | 0.0 | 5 | 8.5 | 3 | 10.0 | 6 | 10.0 | 2 | 6.7 |
|  | G3R | 0 | 0.0 | 0 | 0.0 | 0 | 0.0 | 0 | 0.0 | 0 | 0.0 | 0 | 0.0 |
| Malaise | Any | 5 | 8.3 | 0 | 0.0 | 8 | 13.6 | 2 | 6.7 | 11 | 18.3 | 2 | 6.7 |
|  | G3R | 0 | 0.0 | 0 | 0.0 | 0 | 0.0 | 0 | 0.0 | 1 | 1.7 | 0 | 0.0 |
| Nausea | Any | 4 | 6.7 | 1 | 3.3 | 2 | 3.4 | 1 | 3.3 | 6 | 10.0 | 0 | 0.0 |
|  | G3R | 0 | 0.0 | 0 | 0.0 | 0 | 0.0 | 0 | 0.0 | 0 | 0.0 | 0 | 0.0 |
| Headache | Any | 11 | 18.3 | 4 | 13.3 | 14 | 23.7 | 4 | 13.3 | 12 | 20.0 | 3 | 10.0 |
|  | G3R | 0 | 0.0 | 0 | 0.0 | 0 | 0.0 | 0 | 0.0 | 1 | 1.7 | 0 | 0.0 |

| Mal-020: ages 1-5 years |  | N=89 | | N=39 | | N=89 | | N=45 | | N=89 | | N=41 | |
| --- | --- | --- | --- | --- | --- | --- | --- | --- | --- | --- | --- | --- | --- |
| Symptom |  | N | % | n | % | n | % | n | % | n | % | n | % |
| Injection site pain | Any | 70 | 78.7 | 34 | 87.2 | 75 | 84.3 | 38 | 84.4 | 82 | 92.1 | 32 | 78.0 |
|  | G3 | 1 | 1.1 | 0 | 0.0 | 5 | 5.6 | 1 | 2.2 | 13 | 14.6 | 0 | 0.0 |
| Injection site swelling | Any | 5 | 8.3 | 1 | 3.3 | 10 | 16.9 | 0 | 0.0 | 12 | 20.0 | 0 | 0.0 |
|  | G3 | 1 | 1.7 | 0 | 0.0 | 1 | 1.7 | 0 | 0.0 | 4 | 6.7 | 0 | 0.0 |
| Fever | Any* | 10 | 11.2 | 10 | 25.6 | 8 | 9.0 | 9 | 20.0 | 15 | 16.9 | 5 | 12.2 |
|  | G3R | 0 | 0.0 | 1 | 2.6 | 1 | 1.1 | 1 | 2.2 | 0 | 0.0 | 1 | 2.4 |
| Drowsiness | Any | 8 | 9.0 | 2 | 5.1 | 7 | 7.9 | 4 | 8.9 | 5 | 5.6 | 2 | 4.9 |
|  | G3R | 0 | 0.0 | 0 | 0.0 | 0 | 0.0 | 0 | 0.0 | 0 | 0.0 | 0 | 0.0 |
| Loss of appetite | Any | 7 | 7.9 | 1 | 2.6 | 11 | 12.4 | 9 | 20.0 | 13 | 14.6 | 4 | 9.8 |
|  | G3R | 0 | 0.0 | 0 | 0.0 | 2 | 2.2 | 1 | 2.2 | 2 | 2.2 | 0 | 0.0 |
| Irritability/fussiness | Any | 4 | 4.5 | 0 | 0.0 | 9 | 10.1 | 6 | 13.3 | 24 | 27.0 | 5 | 12.2 |
|  | G3R | 0 | 0.0 | 0 | 0.0 | 0 | 0.0 | 1 | 2.2 | 3 | 3.4 | 0 | 0.0 |

*axillary body temperature ≥37.5°C

N = number of documented doses; n (%) = number (percentage) of doses followed by a local/general symptom.

All local injection site symptoms were considered related to study vaccine.

**Definition of Grade 3 reactions (Notation: G3 = Grade 3; G3R=Grade 3 reaction related to study vaccine)**

Injection site pain Mal-015: spontaneously painful

Mal-020: cries when limb is moved/spontaneously painful

Injection site swelling Mal-015: > 50 mm and persisting for more than 24 hours

Mal-020: > 20 mm

Limited arm motion Mal 015 only: abduction at the shoulder < 30°

Fever Mal-015 and Mal-020: axillary body temperature ≥ 39.0°C

Drowsiness Mal-020 only: drowsiness that prevents normal activity

Loss of appetite Mal-020 only: not eating at all

Irritability/fussiness Mal-020 only: crying that cannot be comforted/prevents normal activity

Other symptoms Mal-020 only: an adverse event is one that prevents normal activity

Unsolicited symptoms were recorded with similar (Mal-015: 6 to 11 year-olds) or lower (Mal-020: 1 to 5 year-olds) frequency in the study vaccine groups compared to the control vaccine groups. Most unsolicited symptoms were mild to moderate in intensity and unrelated to vaccination.

In both Mal-015 and Mal-020, hematocrit values were generally low but comparable among all study groups. A total of six children in the RTS,S/AS02A groups (one in 0.1 ml dose group, five in 0.25 ml dose group) experienced moderate anemia, defined as a hematocrit level of 15% to 24% during the period to 30 days post Dose 3. No case of anemia occurred in the 0.5 ml or control groups. All these children had documented malaria episodes before or at the time that anemia was recorded.

All children were monitored in both Mal-015 and Mal-020 for liver function. Among the 6 to 11 year-olds (Mal-015), two children in the RTS,S/AS02A groups experienced a transient rise in ALT levels judged not related to vaccination and not clinically relevant. In the 1 to 5 year-olds (Mal-020), increases in ALT levels from pre-vaccination were observed in two subjects in RTS,S/AS02A groups and two subjects in the rabies groups. Of these, only one case in the rabies control group was judged clinically relevant; vaccination was discontinued after Dose 1. No other clinically relevant abnormality of hematological or biochemical laboratory parameters was observed.

In the interval to 30 days post Dose 3, a total of six serious adverse events (SAEs) occurred. In the 6 to 11 year-olds (Mal-015), one SAE occurred: a case of bronchopneumonia, reported in a subject in the rabies control group. The event was not considered causally related to vaccination, and the subject fully recovered. Five SAEs were reported in the 1 to 5 year-olds (Mal-020). Among the RTS,S/AS02A recipients, one subject suffered acute malaria with acute upper respiratory tract infection (0.1 ml dose) and one subject suffered cerebral malaria (0.25 ml dose). In the rabies control group three SAEs were reported: acute severe malaria with urinary tract infection and salmonella septicemia, bronchopneumonia with bronchial asthma, and accidental death due to drowning. All SAEs were considered unrelated to study vaccines. Apart from the fatal SAE, all subjects fully recovered and were not withdrawn from the study.

The results of Mal-015 and Mal-020 led to the selection of the 0.25 ml dose of RTS,S/AS02A for future pediatric development.

### Mal-025

The RTS,S/AS02A (0.25 ml dose) vaccine was evaluated in a double-blind, randomized, controlled Phase I study under a new schedule (0, 1, 2 month) in children aged 1 to 4 years in Mozambique (Mal-025). Subjects received RTS,S/AS02A (0.25 ml dose) or *Engerix™-B* hepatitis B vaccine (GSK Biologicals, Rixensart, Belgium). The frequency of solicited symptoms per dose during the 4-day follow-up period for each vaccine group during the study is summarized in Exhibit 3.

As previously observed in Mal-020, local reactions at the site of injection were common. As was observed in Mal-020, swelling at the injection site was the most frequently observed local reaction. Grade 3 swelling was reported after 23% of doses of RTS,S/AS02A (0.25 ml dose) but did not occur following administration of *Engerix™-B*. The incidence of swelling in the RTS,S/AS02A group decreased after Dose 2 of vaccine but increased after Dose 3.

Few solicited general adverse events were reported. In the RTS,S/AS02A group, the most frequently reported solicited adverse events related to vaccination were fever (9.5% after all doses) and loss of appetite (7.5% after all doses). In the *Engerix™-B* comparator group, fever (1.2%) was the only solicited general adverse event related to vaccination. With subsequent doses, there was no increasing trend in solicited general adverse events related to vaccination. The study reported one Grade 3 solicited general adverse event, a fever in the RTS,S/AS02A group. All solicited general symptoms resolved within the 4‑day follow-up period after vaccination. The frequency of solicited symptoms was similar to that previously observed in Mal-020.

Exhibit 3. Frequency of solicited symptoms per dose during the 4-day follow-up period for each vaccine group in Mal-025

|  |  | **RTS,S/AS02A**  **(0.25 ml dose)** | | **Engerix™-B** | |
| --- | --- | --- | --- | --- | --- |
| Mal-025: ages 1-4 years |  | N=84 | | N=83 | |
| Symptom |  | n | % | n | % |
| Injection site pain | Any | 25 | 29.8 | 3 | 3.6 |
|  | Grade 3 | 1 | 1.2 | 0 | 0.0 |
| Injection site swelling | Any | 63 | 75.0 | 37 | 44.6 |
|  | Grade 3 | 19 | 22.6 | 0 | 0.0 |
| Fever | Any* | 12 | 14.3 | 3 | 3.6 |
|  | Grade 3/Related | 1 | 1.2 | 0 | 0.0 |
| Drowsiness | Any | 4 | 4.8 | 1 | 1.2 |
|  | Grade 3/Related | 0 | 0.0 | 0 | 0.0 |
| Loss of appetite | Any | 10 | 11.9 | 1 | 1.2 |
|  | Grade 3/Related | 0 | 0.0 | 0 | 0.0 |
| Irritability | Any | 6 | 7.1 | 2 | 2.4 |
|  | Grade 3/Related | 0 | 0.0 | 0 | 0.0 |

*axillary body temperature ≥37.5°C

N = number of documented doses; n (%) = number (percentage) of doses followed by a local/general symptom.

All local injection site symptoms were considered related to study vaccine.

**Definition of Grade 3 reactions in Mal-025**

Injection site pain Cries when limb is moved/spontaneously painful

Injection site swelling >20 mm

Fever Axillary body temperature ≥ 39.0°C

Drowsiness Drowsiness that prevents normal activity

Loss of appetite Not eating at all

Irritability/fussiness Crying that cannot be comforted/prevents normal activity

Unsolicited adverse events, which were recorded over a 30-day follow-up period after vaccination, occurred with similar frequency in the study vaccine and control groups. No unsolicited event was considered by the investigator to be related to vaccination. The unsolicited events reflect the pattern of childhood morbidity expected in the population. Unsolicited adverse events showed similar frequency and severity in the two treatment groups.

Two children experienced moderate anemia (hematocrit 15 to 24%) during the period to one-month post Dose 3. One child who received RTS,S/AS02A had anemia associated with an acute case of malaria. In the control group, the cause was not identified but the child recovered when administered a course of antibiotics and iron supplementation.

Seven subjects in the control group and three in the RTS,S/AS02A group had an elevation in ALT during the period to one months post Dose 3 (reference range < 60 IU/L). One subject in the control group was observed to have an ALT level of 61 IU/L at a single time point. The subject was clinically well, and because the value was just outside the reference range, he was not investigated further. In the other nine subjects the raised ALT was associated with a viral hepatitis. Five of these subjects had acute hepatitis A. One subject in each group was a chronic carrier of hepatitis B and one subject in the RTS,S/AS02A group was suffering from acute hepatitis B. One other subject who received *Engerix™-B* was HBsAg positive and was a probable chronic carrier of hepatitis B; no confirmatory serology was performed.

No other clinically relevant abnormality of hematological or biochemical laboratory parameters was observed.

Two serious adverse events were reported in each group during the period to one month post Dose 3. One subject in the *Engerix*™-B group contracted *P. falciparum* malaria; a second subject experienced glomerulonephritis secondary to skin lesions. In the RTS,S/AS02A (0.25 ml dose) group, one subject suffered a febrile convulsion 15 days after the second vaccination and another subject suffered from bronchopneumonia. No SAE was considered related to study vaccine. All subjects made a full recovery and none was withdrawn from the study (16).

The clinical experience with AS02A in adults and children when used with FMP1 is described in Section 3.5.

## The FMP1 Antigen

The falciparum merozoite protein 1 (FMP1) study antigen is a lyophilized recombinant antigen produced in, and purified from, *E. coli* bacteria at the Walter Reed Army Institute of Research, Silver Spring, MD, USA. This antigen consists of the 42‑kDa carboxy-terminal end (392 amino acids) of the merozoite surface protein-1 (MSP-1_42_) from the malaria parasite, *P. falciparum,* 3D7 clone. The protein is expressed as a fusion protein where six histidines are added to the N terminal to facilitate purification. The FMP1 product used in the previous RTS,S/FMP1 combination trial (Mal-019; under FDA IND #9772) in the previous phase I trials in adults and children at Kombewa (Mal-024, Mal-031; under FDA IND #9202), and to be used in the present study, is designated Lot 1034, and was prepared according to BPR-425-01. This lot was produced from Purified Bulk FMP1 Lot 0774, BPR-335-03. The lyophilized antigen will be dissolved in approximately 0.5 ml of AS02A adjuvant prior to injection.

The total amount of antigen contained in a single adult dose is approximately 50 μg. This dose was chosen on the basis of results from phase I studies in malaria-naïve adult subjects (Mal-019 and Mal-014) as described in Section 3.5.2. The specific dose of 50 μg for the present study was selected on the bases of data regarding safety, reactogenicity, and immunogenicity from Mal-031 (see Section 3.5.2).

## Experience with the FMP1/AS02A Malaria Vaccine

### Pre-clinical Trials

The FMP1/AS02A formulation was safe, well tolerated, and highly immunogenic in a pre-clinical trial conducted in rhesus macaques (*Macaca mulatta*) performed at the Armed Forces Research Institute of the Medical Sciences (AFRIMS) in Bangkok, Thailand. Eight monkeys were immunized intramuscularly on a 0, 1, 3 month schedule using a standardized safety and immunogenicity model. All eight immunized monkeys seroconverted to the immunogen. Group mean antibody titers against a 19 kDa subunit of the immunogen rose to 10,000 ELISA units after 2 doses and to 17,000 units after 3 doses. Rhesus antibody against *P. falciparum*-parasitized red blood cells was also highly positive in an indirect fluorescence antibody assay.

Clinical grade lots of FMP1 adjuvanted with AS02A have been administered to mice and guinea pigs. No significant local or systemic toxicity was observed in any of the animals. Immune responses to the formulation in mice indicated excellent antibody responses to the FMP1 antigen.

### Clinical Trials

Mal-014

An open label, phase I, dose-escalation study to evaluate the safety, reactogenicity, and immunogenicity of FMP1 with AS02A adjuvant (Mal-014) was undertaken in October 2000 at the WRAIR. Fifteen human subjects were randomized to receive either approximately 10μg (N=5), 25μg (N=5), or 50μg (N=5) doses of vaccine on a 0, 1, 3 month schedule by intramuscular injection. The ratio of adjuvant to antigen was constant (i.e., 0.10 ml, 0.25 ml, or 0.50 ml of AS02A, respectively). After 3 doses, no Grade 3 reaction (defined as a reaction that prevents normal day-to-day activities) and no serious adverse event occurred. The laboratory tests have been normal except for an occasional elevated CPK level detected both at the time of immunization and 48 hours after immunization probably related to physical activity of the subjects. All 15 individuals seroconverted after a single immunization. Boosting of antibody levels occurred after the second and third doses. The antibody titers against MSP-1_42_ are summarized below in OD units (the dilution that gives an OD_415_= 1).

Exhibit 4. Immunogenicity of three different doses of the FMP1/AS02A vaccine (Mal 014)

| **OD Units (000’s)**  (N=5 in each arm) | | | | | | | | | | | | |
| --- | --- | --- | --- | --- | --- | --- | --- | --- | --- | --- | --- | --- |
| **Cohort** |  | **Day 0** |  | **Day 14** |  | **Day 28** |  | **Day 42** |  | **Day 84** |  | **Day 98** |
| **1/5 Dose (10 μg)** |  |  |  |  |  |  |  |  |  |  |  |  |
| Arithmetic mean |  | 0.012 |  | 0.3 |  | 0.5 |  | 18.1 |  | 7.4 |  | 32.6 |
| Standard Deviation |  | 0.008 |  | 0.2 |  | 0.2 |  | 8.4 |  | 6.3 |  | 24.0 |
| Geometric mean |  | 0.010 |  | 0.3 |  | 0.4 |  | 16.4 |  | 5.7 |  | 26.6 |
|  |  |  |  |  |  |  |  |  |  |  |  |  |
| **1/2 Dose (25 μg)** |  |  |  |  |  |  |  |  |  |  |  |  |
| Arithmetic mean |  | 0.028 |  | 1.3 |  | 2.5 |  | 44.2 |  | ND |  | 57.8 |
| Standard Deviation |  | 0.006 |  | 1.9 |  | 2.6 |  | 21.2 |  | ND |  | 24. 2 |
| Geometric mean |  | 0.028 |  | 0.6 |  | 1.8 |  | 40.7 |  | ND |  | 53.6 |
|  |  |  |  |  |  |  |  |  |  |  |  |  |
| **Full Dose (50 μg)** |  |  |  |  |  |  |  |  |  |  |  |  |
| Arithmetic mean |  | 0.032 |  | 0.7 |  | 1.0 |  | 32.5 |  | 15.9 |  | 50.1 |
| Standard Deviation |  | 0.032 |  | 0.4 |  | 0.3 |  | 19.3 |  | 5.7 |  | 30.0 |
| Geometric mean |  | 0.022 |  | 0.6 |  | 1.0 |  | 28.4 |  | 14.9 |  | 42.8 |

ND: Not done

NB: Although the above results suggest little or no difference between recipients of 25 and 50 μg, IFA titers against whole merozoites were higher in the latter group than in the former.

Mal-019

Following Mal-014, the WRAIR conducted a double‑blind, phase I/IIa study (Mal‑019) to evaluate the potential synergy of FMP1/AS02A and RTS,S/AS02A. This study assessed whether concomitant administration of the two vaccines interfered with immunogenicity. The study enrolled 71 volunteers in March 2001 and followed them until January 2002. Each vaccine study arm had 15 people; the challenge (unvaccinated) control had 11 people. The study arms were:

Group A: FMP1/AS02A + RTS,S/AS02A administered concomitantly at separate sites of injection

Group B: FMP1/AS02A + RTS,S/AS02A administered at one injection site and saline at the opposite site

Group C: FMP1/AS02A + AS02A concomitantly at separate sites of injection

Group D: RTS,S/AS02A + AS02A administered concomitantly at separate sites of injection.

Group E: Unvaccinated control

Study endpoints included safety, immunogenicity, reactogenicity, and efficacy (defined as either delay or prevention of parasitemia in comparison to controls as determined by light microscopy). The primary efficacy analysis aimed to determine major agonist or antagonist effects of FMP1 on RTS,S/AS02A-mediated protection. Secondary analyses included experimental molecular analyses to determine delay in release of hepatic merozoites and correlation of efficacy results with the functional and quantitative antigen-specific antibody.

Fifty-five subjects (11 in A, 12 in B, 10 in C, 12 in D, and 10 in E) completed the study. All volunteers received the first two immunizations (except Group E, which did not receive any immunization). Pain at the site of injection was the most frequently reported local symptom at 50%, 32%, 32%, and 19% in groups A, B, C, and D respectively. One subject in each group had Grade 3 pain in the first four days after vaccination. Two cases of Grade 3 redness were reported. Headache was the most commonly reported general symptom with a frequency of 23% and 19% in groups A and D, respectively. Myalgia was reported in 23% of subjects in group B. Fever occurred in 11% of subjects in group C. One dose was followed by Grade 3 malaise in group B. All local symptoms resolved within 4 days after vaccination and there was no apparent increase in the incidence of symptoms from dose to dose.

A total of 82 unsolicited symptoms were reported by 39 subjects following 60 doses in the 30 days following vaccination during the study. One volunteer in group B experienced a Grade 3 symptom of wrist pain which was not related to the vaccination and resolved without sequelae after 23 days. No abnormal laboratory change was detected during the study.

Two SAEs were reported: one subject in group D reported chest pain after anesthesia, and one subject in group C reported a nervous breakdown. Both of these SAEs were deemed unrelated to vaccination.

All subjects in groups A, B, and D were anti-CSP seropositive after the second dose (at day 42) and remained seropositive at day 134 after challenge. All subjects in groups A, B, and D were seropositive with respect to anti-HBs and all were seroprotected (anti-HBs titers > 10 mIU/ml) after the second dose at day 42. The geometric mean titers (GMT) were 27, 23, and 28 ×10^3^ mIU/ml, respectively, after the third dose. All subjects tested at day 134 maintained seroprotection. All subjects in groups A, B, and C had seroconverted with respect to anti-MSP-1 (allotype 42) at day 42 after the second dose; the seroconversion was maintained at day 134 after challenge.

All 11 infectivity controls contracted malaria during the challenge phase. Their mean and median pre-patent periods were both 11 days. The median pre-patent periods in the infected volunteers who received RTS,S/AS02A vaccine were significantly prolonged by 2 to 3 days. (p<0.001). There was no protection or delay in the pre-patent period among the volunteers receiving FMP1/AS02A. There was no difference in the pre-patent period for volunteers receiving RTS,S/AS02A with FMP1/AS02A as compared to those receiving RTS,S/AS02A.

In conclusion, the study showed that all the vaccination regimens were safe with acceptable reactogenicity and were immunogenic for anti-CSP repeat, anti-MSP-1 antibody titer, and anti‑HBs antibody titer. FMP1/AS02A alone provided no protection against malaria infection. This study provides evidence that the concomitant administration of FMP1 does not interfere with the efficacy of RTS,S.

This combination study of RTS,S and FMP1 demonstrated the non-interference of MSP-1 with the previously described protective effect of RTS,S. FMP1/AS02A vaccine, a blood stage vaccine, is not expected to prevent malaria infection but rather to affect the clinical disease presentation. The clinical sporozoite challenge model is not designed to, and not capable of, predicting efficacy against clinical malaria (as opposed to malaria infection); therefore, this challenge study had limited ability to detect the protective effect of FMP1. In this model, treatment is required upon detection of parasitemia, and thus Mal-019 cannot address whether FMP1/AS02A could limit parasitemia with the net effect of attenuating clinical disease. The present study will assess whether FMP1/AS02A can limit disease.

Mal-024

In 2002, following the Mal-019 challenge study, 40 participants (14 females and 26 males) were enrolled into the Mal-024 study in Kombewa, Kenya. Subjects were randomized 1:1 to receive FMP1/AS02A or *Imovax*®. (Throughout the remainder of this section, we refer to the FMP1/AS02A group simply as the FMP1 group.)

Each study arm had 7 females and 13 males. The mean age ± standard deviation was 33.7 ± 9.6 years for the FMP1 arm and 32.9 ± 11.4 years for the *Imovax*® arm. All 40 participants received the first two immunizations. Three did not receive the third immunization but were followed according to the protocol. One participant who received *Imovax*® was excluded from the third dose because of transient transaminase elevation; a second who had received *Imovax*® missed the third immunization because of noncompliance with the visit schedule; and a third was excluded because he left the study area.

The most common side effect related to immunization was pain at the site of injection. Seventeen FMP1 and 10 *Imovax*® vaccinees complained of pain at the injection site within 24 hours of immunization (p = 0.04). In most cases the pain resolved within 48 hours. There was no other difference in vaccine-related signs and symptoms.

Ten documented SAEs occurred in nine subjects during the study - seven in the *Imovax*® group and three in two subjects who received FMP1. In the *Imovax*® group, three volunteers contracted malaria, two developed sepsis, one experienced a ruptured ectopic pregnancy, and one experienced gastroenteritis. In the FMP1 group, one subject developed simultaneous pneumonia and malaria; a second contracted pneumonia, and after recovery, had dysenteric gastroenteritis/possible typhoid fever. No SAE was causally related to immunization. The baseline GMT was slightly higher in the *Imovax*® group (~24 × 10^3^ versus ~18 × 10^3^); however, by study day 44 (14 days after the second immunization), the GMT in the FMP1 group had surpassed that in the *Imovax*® group. From that point forward, the FMP1 group’s GMT remained higher than the *Imovax*® group’s (17).

Please see Exhibit 4a for a summary of solicited symptoms.

Exhibit 4a: Incidence of Grade 3 solicited symptoms during eight-day follow-up period, by immunization and overall, for all subjects (ages 18-55 years) in MAL-024

|  | ***Imovax®*** | | |  | **FMP1/AS02A** | | |  | **Overall** | | |  | **p-value** |
| --- | --- | --- | --- | --- | --- | --- | --- | --- | --- | --- | --- | --- | --- |
| Symptom | n | N | % |  | n | N | % |  | n | N | % |  |  |
| Any Symptom Any  G3 | 18  8 | 20  20 | 90  40 |  | 20  9 | 20  20 | 100  45 |  | 38  17 | 40  40 | 95  43 |  | 0.49  1.00 |
| Local symptom Any  G3 | 17  8 | 20  20 | 85  40 |  | 19  9 | 20  20 | 95  45 |  | 36  17 | 40  40 | 90  43 |  | 0.60  1.00 |
| General symptom Any  G3 | 8  0 | 20  20 | 40  0 |  | 12  0 | 20  20 | 60  0 |  | 20  0 | 40  40 | 50  0 |  | 0.34  1.00 |

N = number of volunteers who received immunization; n (%) = number (%) of who experienced at least one symptom during follow-up

p-values are based are Fisher’s exact test

Mal-031

Following the successful Mal-024 adult phase I trial in Kombewa, a phase I trial (Mal-031) in children was launched in August 2003. Follow-up ended in September 2004. The study enrolled 135 children, 58 males and 77 females, into three dose cohorts: A (10 µg), B (25 µg), and C (50 µg). Each cohort had 45 children. The subjects were further subdivided into three age cohorts of 12 to 23, 24 to 35, and 36 to 47 months (15 children each). In each of the nine age cohorts, 10 children received FMP1/AS02A vaccine and five received *Imovax*® (the comparator). The vaccines were administered in a dose escalation fashion, with each cohort on a 0, 1, 2 month schedule.

The interim safety data report showed that the most common solicited adverse events were pain and swelling at the injection site. More children who received FMP1/AS02A experienced more local symptoms than those who received *Imovax*®. About 30% of children had local symptoms during the first immunization; the percentage decreased in doses 2 and 3. Grade 3 local symptoms occurred in fewer than 10% of children in any dose cohort. These local symptoms resolved within 48 to 72 hours. The most common general symptoms were fever and irritability; these also resolved within 24 to 48 hours. No incidence of drowsiness was noted. The most common unsolicited adverse events during the 30-day period following each vaccination were malaria and respiratory tract infection. About 76% of those who received FMP1/AS02A vaccine and 87% of those who received *Imovax*® vaccine experienced at least one episode of malaria. About 80% in both FMP1/AS02A and *Imovax*® vaccine arms had respiratory tract infections. No subject experienced a Grade 3 vaccine‑related unsolicited AE.

The Day 74 GMT titers (14 days after the third vaccination) showed a five-fold increase in the 10 µg dose and a greater than 15-fold increase in the 25 and 50 µg dose compared to the baseline GMT titers. (See Figure A). Approximately 80% of study subjects receiving the 50 µg dose achieved a four-fold or greater rise in anti-FMP1 titer at study day 90 (i.e., 30 days after the third dose), which is a higher percentage than in either the 10 or 25 µg doses. The 50 µg dose gave a 16-fold increase in the GMT of the anti-MSP-1 antibody titers in most of the children (see Figure B). The DSMB reviewed the interim data report and found FMP1/AS02A to be safe and immunogenic in this pediatric population. The DSMB then approved proceeding with the Phase IIb trial with the 50 µg dosage as an appropriate dose for further evaluation.

Please see Exhibit 4b for details of solicited symptoms.

Exhibit 4b: Frequency of solicited symptoms per dose during the 8-day follow-up period for each vaccine group (of subjects aged 12-48 months) in MAL-031

|  | **FMP1/AS02A**  **(10 ug)** | | **Imovax®** | |  | **FMP1/AS02A**  **(25 ug)** | | **Immovax®** | |  | **FMP1/AS02A**  **(50 ug)** | | **Immovax®** | |
| --- | --- | --- | --- | --- | --- | --- | --- | --- | --- | --- | --- | --- | --- | --- |
|  | N=88 | | N=42 | |  | N=88 | | N=43 | |  | N=84 | | N=42 | |
| Symptom | n | % | n | % |  | n | % | n | % |  | n | % | n | % |
| Injection site pain Any  G3 | 12  0 | 40  0 | 2  0 | 13  0 |  | 24  2 | 80  7 | 1  0 | 7  0 |  | 26  1 | 87  3 | 2  0 | 13  0 |
| Injection site swelling Any G3 | 6  0 | 20  0 | 0  0 | 0  0 |  | 7  0 | 23  0 | 0  0 | 0  0 |  | 15  0 | 50  0 | 0  0 | 0  0 |
| Fever Any G3 | 14  4 | 47  13 | 6  0 | 40  0 |  | 12  2 | 40  7 | 8  2 | 53  13 |  | 16  0 | 53  0 | 6  2 | 40  13 |
| Drowsiness Any G3 | 0  0 | 0  0 | 0  0 | 0  0 |  | 0  0 | 0  0 | 0  0 | 0  0 |  | 0  0 | 0  0 | 0  0 | 0  0 |
| Loss of appetite Any  G3 | 13  0 | 43  0 | 6  0 | 40  0 |  | 15  0 | 50  0 | 8  0 | 53  0 |  | 14  0 | 47  0 | 5  0 | 33  0 |
| Irritability Any  G3 | 2  0 | 7  0 | 0  0 | 0  0 |  | 4  0 | 13  0 | 1  0 | 7  0 |  | 2  0 | 7  0 | 0  0 | 0  0 |

N = number of documented doses; n (%) = number (%) of doses followed by a local/general symptom.

All local injection site symptoms were considered related to the study vaccine.

Figure A. Reverse cumulative plot of Day 74 anti‑FMP1 titers, by dose cohort

Figure B. Reverse cumulative plot of ratio of Day 90 versus Day 0 anti FMP1 titers

## Justification of Dosing

### Justification of 0, 1, and 2 Month Dosing

The Phase I trials in Kombewa Division, Kenya, Mal-024 (40 adults) and Mal-031 (135 children) both used a 0, 1, 2 month schedule. This schedule is compatible with incorporation into the World Health Organisation’s Expanded Programme of Immunisations (EPI) and thus is relevant to our plans for further development of the vaccine in pediatric populations.

For justification of the 0, 1, and 2 month dosing of the comparator vaccine, see Section 3.7.

### Justification of the 50 µg Dose

Preliminary data from Mal-031 indicate no increased incidence of adverse events associated with the 50 µg dose compared to the lower doses. Moreover, approximately 80% of study subjects receiving the 50 µg dose achieved a four-fold or greater rise in anti-FMP1 titer at study day 90 (i.e., 30 days after the third vaccination), a percentage higher than either the 10 or 25 µg dose. The 50 µg dose gave a 16-fold increase in the GMT of the anti-FMP1 antibody titers in most children. The assumption that vaccine efficacy will increase with level of antibody titer (a surrogate marker) has led us to select the high (50 µg) dose for further evaluation (see Section 3.5.2).

## Comparison Vaccine

### The Vaccine

A rabies vaccine (either *Imovax*® [HDCV rabies vaccine] or (*Rabipur*®/*RabAvert*® [PCEC rabies vaccine]) will serve as the comparator in this study. The recommended doses and schedules are the same for infants, children, and adults (see Section 9.2 for more details). Rabies vaccine is a parenteral preparation of inactivated rabies virus used to promote active immunity to rabies in individuals exposed to the disease or virus. Prophylactic doses of rabies vaccine are administered to certain groups of people at high risk for acquiring rabies infection. Advantages of providing pre-exposure vaccination include reduced number of doses of the vaccine after a rabies exposure, elimination of need for rabies immune globulin post-exposure, possible protection in persons whose post-exposure therapy is delayed or unavailable, and possible protection for persons at risk for inapparent exposures to rabies. Pre-exposure vaccine is recommended for individuals at risk of occupational exposure to the rabies virus (e.g., physicians, veterinarians, laboratory workers, animal handlers). Pre‑exposure vaccination is also recommended for other persons whose activities involve frequent contact with rabies virus or potentially rabid animals such as bats, raccoons, skunks, cats, or dogs. Pre-exposure vaccination is commonly recommended in international travelers who are likely to come in contact with animals in areas where dog rabies is enzootic and immediate access to appropriate medical care, including biologics, might be limited. In our study area, as throughout Kenya (18), most people are at risk for dog and cats bites, but are rarely vaccinated against rabies. Since rabies vaccine is not widely available in the public health facilities, provision of the product is of great benefit to the study participants.

The 200 comparison subjects will receive this comparator vaccine during the active phase of the study; it will also be offered free of charge to the remaining 200 children after study completion.

### Vaccine Immunogenicity

When rabies vaccine is administered according to the recommended immunization schedule (days 0, 7, 21), nearly all subjects attain a protective titer. In two studies carried out in the United States in 101 subjects, all subjects attained antibody titers > 0.5 IU/ml by day 28. In studies carried out in Thailand (22 subjects), and in Croatia (25 subjects), all subjects achieved antibody titers above 0.5 IU/ml by day 14 (injections on days 0, 7, 21) (19-23). High antibody titers have also been demonstrated with off-label immunization with rabies vaccine. Among participants in England, Germany, France, and Belgium who received two doses one month apart, nearly all of the participants developed specific antibody; the geometric mean titer for the group was 10 IU (24-27). In a comparative study of two different rabies vaccines administered by the intramuscular route on either a 0, 7 and 21 day schedule or on a 0, 28 and 56 day schedule, the peak group GMT of rabies neutralizing antibody in international units (IU) was higher for both the chick embryo-basede vaccines (8.4 vs. 14.1 IU) and for the human diploid cell-based vaccine (13.7 vs. 19.3 IU) (21). The proposed immunization schedule of 0, 1, and 2 months is therefore expected to be highly successful in conferring protective immunity against rabies among the control participants.

### Vaccine Safety

Chiron’s PCEC rabies vaccine (purified chick embryo cell rabies vaccine; trademarked *RabAvert*® in the US and *Rabipur*® outside the US) is effective pre-exposure immunization and post-exposure treatment against rabies for all age groups. It is the most widely used high quality cell culture vaccine with more than 55 million doses administered in ~70 countries worldwide. This vaccine is WHO recommended and has been FDA approved since 1997. It is also licensed by MHRA, the UK regulatory agency, and is widely used in Kenya.

Aventis Pasteur’s *Imovax*® rabies vaccine is a human diploid cell vaccine (HDCV) whose safety and efficacy have been established in children. Local and/or mild systemic reactions may occur after injection, but these reactions are usually transient and do not contraindicate continuing immunization. In a study using five doses of HDCV, about 25% of recipients reported local reactions such as pain, erythema, and swelling or itching at the injection site (28). About 20% of recipients reported mild systemic reactions such as headache, nausea, abdominal pain, muscle aches, and dizziness (29). Two cases of neurologic illness resembling Guillan-Barre syndrome, a usually transient neuroparalytic illness, and a focal sub-acute central nervous system disorder temporally associated with HDCV, have been reported (30-32). Aventis Pasteur’s *Imovax*® rabies vaccine is FDA approved.

Systemic allergic reactions characterized by generalized urticaria and in some cases by arthralgia, angioedema, fever, nausea, and vomiting have been reported following administration of HDCV. These reactions, although uncommon in primary administrations, have been reported in up to 7% of persons receiving a booster dose (33).

In a comparative study in a pediatric population, both a HCDV and chick embryo-based rabies vaccines were found to have no statistically significant differences in adverse events (19). The study will plan to use the same rabies vaccine (*Rabipur*®) throughout the study, but in the event *Rabipur*® is not available, the *Imovax*® rabies vaccine will be used.

# JUSTIFICATION

## Malaria in Western Kenya

Malaria in western Kenya is holoendemic. Intense transmission occurs primarily by bites of the *Anopheles gambiae* mosquito. *P. falciparum* parasitemia is present in over 90% of malaria cases. *P. ovale* and *P. malariae*, which together constitute less than 10% of cases, are usually present in mixed infections with *P. falciparum.* Malaria transmission occurs all year but at a very high level during the two rainy seasons. The “long rainy season” of late March through May produces intense transmission from April through August; the “short rainy season” of October through December produces another, somewhat less intense, transmission season from November through January. The intensity of both transmission seasons can be predicted by the amount of rain seen in the preceding month and correlates with both the total population of female anopheline mosquitoes and the entomological inoculation rate (EIR, i.e., the number of infected mosquito bites per person per unit time). Cumulative malaria attack rates have been roughly 95% during the long rains and 75% during the short rains. The adult population of several villages not far from the study site has been the subject of several malaria chemotherapy and epidemiology studies. These studies typically demonstrate about 90% incidence of parasitemia over 12 weeks during a high transmission period. Approximately 25% of these infected adults will become symptomatic at some time during that period. Over the course of two disease seasons a large-scale longitudinal cohort project recently undertaken in Kisumu District (34) documented an 83% malaria parasite prevalence in the proposed study district and in the proposed study age groups (1 to 4 year-olds). The investigators of the present study conducted a longitudinal epidemiological study in 2003-2004 that indicates that monthly attack rates range from 20 to 55% in children aged 1 to 3 years in the proposed study area (unpublished data). As a conservative estimate for the purpose of calculating the sample size of this study, we have assumed that 50% of children will develop clinical malaria (as defined by the primary case definition) during the efficacy follow-up period of this study from study day 71 through study day 240. All individuals in this population are parasitemic multiple times over a lifetime. Undoubtedly, children in this population are malaria-exposed.

The Walter Reed Project (WRP) and the Kenya Medical Research Institute (KEMRI) have been involved in malaria research in western Kenya for many years. Their studies cover nearly every aspect of the disease: epidemiology, entomology, immunology, hospital-based treatment trials, and community-based studies of antimalarials. The WRP/KEMRI laboratories near Kisumu have served as the base for both phase I and II trials of candidate malaria vaccines and antimalarial drugs over the past decade. In spite of these research efforts, malaria infections in this area continue unabated and improved control strategies are required.

## Target Population

Children are the population with the highest rates of morbidity and mortality due to malaria, accounting for more than one million deaths annually in sub-Saharan Africa. Malaria is a significant cause of morbidity and mortality in both adults and children in western Kenya. In this area, the burden of malaria falls particularly hard on the younger age groups. Worldwide, the populations at risk for malaria include not only the infants, children, and adults in malaria-endemic regions, but also non-immune travelers to malarious areas including vacationers and deploying military personnel. A safe and effective vaccine that prevents infection, or even clinical symptoms of *P. falciparum* malaria, would be a milestone public health achievement and would become a mainstay in efforts to control this serious infectious disease around the world. The increasing prevalence of drug resistance in various malaria strains makes an effective vaccine an international priority in the struggle for control of this devastating disease.

The target population for this vaccine is children at risk for clinical disease (including severe disease) due to infection with *P. falciparum*. FMP1 can only be tested for proof of concept in this population, as there is no predictive animal model and no reliable challenge system to detect efficacy against clinical malaria. As described above, this vaccine has been tested in malaria‑naïve adults in the United States to establish safety and reactogenicity as well as to identify a dose for further evaluation. Subsequent evaluation in malaria-exposed Kenyan adults and children found it safe, well tolerated, and immunogenic in these populations. Three different doses and a control vaccine have been evaluated in 135 Kenyan children aged between 12 and 47 months (Mal-031). Data pertaining to 30 days post-dose 3 are described in section 3.5.2. This study aimed to assess long-term safety and immunogenicity. The AS02A adjuvant has been tested in combination with FMP1, as well as with the RTS,S vaccine in adults and children aged one year and older with prior (and ongoing) exposure to *P. falciparum* malaria. Both AS02A-containing vaccines have been found safe and well tolerated in adults and children.

The next logical step in the clinical development of FMP1/AS02A is to evaluate its efficacy in the pediatric population in a cautious, step-wise manner as described in this protocol. If this vaccine confers a significant delay in the onset of clinical episodes of malaria, it may be further developed either alone or with a pre-blood stage antigen as a combination vaccine, as indicated by the degree of efficacy demonstrated and by the results of a separate Phase IIb study. If the vaccine confers no reduction in clinical episodes of malaria, and there is a significant reduction in the episodes of parasitemia due to vaccine‑homologous *P. falciparum* strains, this vaccine may be developed in combination with a second MSP-1 vaccine based upon the FVO allele. If the vaccine confers no reduction in clinical episodes of malaria, and there is no reduction in the episodes of parasitemia due to vaccine‑homologous *P. falciparum* strains, the vaccine formulation will not be further developed.

# OBJECTIVES and HYPOTHESIS

The primary and secondary objectives pertain to children living in western Kenya and aged 12 to 47 months at the time of randomization.

## Primary

- To assess the efficacy of the FMP1/AS02A malaria vaccine on clinical episodes of *P. falciparum* malaria during the Efficacy Follow-up Period (EFP)

## Secondary

***Efficacy***

- To assess the efficacy of the FMP1/AS02A malaria vaccine against clinical episodes of *P. falciparum* malaria of differing severity during the EFP.

***Safety and Immunogenicity***

- To assess solicited adverse events occurring in the seven days following each dose of vaccine
- To assess unsolicited adverse events occurring in the 30 days following each dose of vaccine
- To assess serious adverse events occurring after the first dose of vaccine to the end of the EFP
- To assess the immunogenicity of the FMP1/AS02A malaria vaccine from the first dose of vaccine to the end of the EFP
- To assess the association between MSP-1 antibody titers and the subsequent risk of clinical malaria during the EFP

## Tertiary

The tertiary objectives pertain to the Addendum Efficacy Follow-up Period (AEFP).

***Safety and Immunogenicity***

- To assess the safety of the FMP1/AS02A malaria vaccine
- To assess the immunogenicity of the FMP1/AS02A malaria vaccine

***Efficacy and Exploratory***

- To assess the efficacy of the FMP1/AS02A malaria vaccine against clinical episodes of *P. falciparum* malaria
- To assess the biological effects of the FMP1/AS02A malaria vaccine

## Hypothesis

This trial is testing the null hypothesis that during the EFP the time to first clinical episode of *P. falciparum* malaria, according to the “Primary Case Definition”, is the same in the FMP1/AS02A and comparator vaccine arms.

Success in the context of evaluating this time is defined as a significant two-sided log‑rank test statistic (p-value <0.05), where time to first clinical episode is longer in the FMP1/AS02A arm than in the comparator arm.

# STUDY DESIGN and METHODOLOGY

## Overview

- Double-blind, randomized, controlled, phase IIb (pilot efficacy) field trial of a candidate antigen/adjuvant
- One study center, with 12 outlying (satellite) field stations
- Screening will be performed no more than 45 days prior to the first inoculation
- 400 subjects will be randomized 1:1 between two arms (FMP1/AS02A and rabies vaccine)
- Immunization schedule will be 0, 1, and 2 months for both study arms
- The four-week intervals between doses may be extended for up to two additional weeks if *temporary suspension* (see Section 6.9.1) is deemed advisable because of SAEs or other concerns
- All subjects will be randomized on the day of first vaccination
- Route of inoculation will be by IM injection into the left anterolateral thigh muscle (unless compelling reason for an alternate injection site is evident)
- Study duration will be approximately about 14 months per subject
- Seven-day follow-up period for solicited adverse events (five visits: vaccination day plus post-vaccination days 1, 2, 3, and 6)
- Thirty day follow-up period for unsolicited adverse events (vaccination day plus 29 subsequent days)
- Follow-up of serious adverse events (SAEs) for study duration
- Efficacy Follow-up Period (EFP): Starting 14 days after the third vaccination (Study Day 71), active case detection commences with visits approximately every 28 days to the WRP Kombewa clinic and terminates after six months (o/a Study Day 240): EFP = 169 days = six (28 day) months
- The primary study analysis for all endpoints will be done on the cleaned EFP database after a data-lock-point
- Addendum Efficacy Follow-up Period (AEFP): Starting with the end of the EFP (o/a Study Day 240), active case detection commences with visits approximately every 28 days to the WRP Kombewa clinic and terminates after 10 months (o/a Study Day 364); AEFP = 125 days = four and a half (28 day) months. This portion of the trial is partially unblinded.
- The study addendum analysis for all endpoints will be done on the AEFP database after a data-lock-point
- Malaria cases will be detected by active and passive means
- Active case detection will be through scheduled, facilitated visits by participants to the Kombewa clinic and scheduled, field worker visits to participants’ homes
- Passive case detection will take place by unscheduled, self-presentation of participants to the Kombewa Clinic
- At scheduled clinic visits, blood samples from all subjects will be taken to determine parasite density and hemoglobin levels. At home visits, only subjects with fever or history of fever within 24 hours (or other illness) will be transported to the clinic and sampled for detection of parasite densities and hemoglobin.
- The primary study analysis conducted at about month 10 (on the EFP data through SD 240) will be considered a “final” analysis; the study will continue an additional 4 months in a partially unblinded fashion (the AEFP data).

## Number of Subjects and Number of Study Arms

The study will randomize 400 subjects, irrespective of gender or age, who fulfill all enrollment criteria. The 400 subjects will be randomized 1:1 between the two arms (FMP1/AS02A and comparator).

## Study Center

### The Walter Reed Project Kombewa Clinic

The Walter Reed Project (WRP) Kombewa Clinic (WRP KC) is located in the village of Kombam (near Kombewa Town), in Kombewa Division, Kisumu District, Nyanza Province, Kenya. Kombewa Town is about 40 kilometers (40 minutes drive) west of Kisumu Town.

The WRP KC is located directly across the Kombewa-Maseno road (a murram road) from the Kombewa Sub-District Hospital (KSDH) where it is less than one kilometer north of the Kisumu-Bondo road (a macadamized road). (The KSDH, a Kenya Ministry of Health sub-district hospital, is an inpatient and outpatient facility; however, it is not to be utilized by study subjects.)

The WRP KC is readily accessible by foot or ground vehicle to all study subjects. It consists of a clinical laboratory equipped to carry out malaria diagnosis and basic clinical laboratory assays (such as measurement of serum chemistries and complete blood counts), a phlebotomy room, an outpatient facility that includes six examination/consultation rooms, a pharmacy, an active records room, and a records archive. Subject records and CRFs will be kept in locked cabinets in the latter two rooms. The facility has a dedicated data entry room, offices for clinical officers and CRCs, on-call rooms, an urgent treatment room, a four-bed observation suite, a washroom, a general store, and a kitchen with dining area.

The PI, associate investigators, and clinical officers (physician assistants) carry out clinical evaluations, including basic laboratory studies, at the WRP KC. Management of symptomatic malaria during this study will be undertaken here as detailed in Section 7.3. During the course of evaluation for malaria in the study subjects, other acute illnesses such as upper respiratory infections and other minor bacterial or viral infections may be uncovered. The pharmacy at the WRP KC will provide subjects with common over-the-counter analgesics such as paracetamol, as well as antibiotics (for the treatment of minor infections) as prescribed by the clinicians. The KC will be staffed at all times (24 hours a day, 7 days a week) by at least one clinical officer and one community nurse. A medical officer (physician) will be on site during working hours Monday through Friday. Additionally, a pediatrician will be on site on most working days and will always be available by telephone (See SOP 501.03: After Hours Care of Subjects).

This facility recently underwent expansion with the construction of a two-story annex block including new office spaces, more commodious general storage areas, a secure records archive room, meeting rooms, and a library.

### Field Stations and Follow-up Centers in Kombewa Division

To facilitate accessibility to the study participants, several field stations have been designated within Kombewa Division; all are located within one-mile radii of the homes of study subjects. Twelve field stations, of which three will be designated as field follow-up centers, will be utilized. Field stations and follow-up field centers are intended to be points of first contact for sick children; therefore, they are staffed 24 hours a day and are equipped to communicate with the WRP KC to summon transportation. (See Appendix D for details.)

### The New Nyanza Provincial General Hospital, Kisumu

The WRP KC does not have an inpatient facility or capability. Furthermore, blood transfusions cannot be administered there and a pediatrician is not usually present after hours or on weekends. If such procedures or expertise are required, or if hospital admission is deemed necessary, study personnel will transport the subject to the New Nyanza Provincial General Hospital in Kisumu. This hospital includes a 44-bed pediatric inpatient ward with an attached, dedicated laboratory staffed by WRP personnel. Inpatients, including any admitted study subjects, receive care overseen by Kenyan pediatricians. Three pediatricians are on staff (one of whom is employed by the WRP) and there are routine 24-hour nursing coverage, and blood transfusion and oxygen supplementation capabilities. In November or December 2004, the pediatric department plans to move to a new, more spacious building immediately adjacent to the existing hospital compound. This new facility will represent a significant upgrade with expansion to 64 beds and new laboratory, pharmacy, and outpatient departments. Limited laboratory support will also be available at the new WRP-Kisumu headquarters building adjacent to the new pediatric ward.

## Inclusion Criteria

- A healthy male or female child, 12 to 47 months of age on the day of screening
- Written informed consent obtained from at least one parent/guardian before study start
- Available to participate for the study duration (about 14 months)

## Exclusion Criteria

**The following criteria will be checked at the time of study entry. If any apply at the time of study entry, the subject will not be enrolled in the study. Additionally, parents will be queried on these criteria immediately before the second and third vaccinations, and any new information suggesting presence of one or more will serve as “elimination criteria” whereby subjects will have vaccination deferred pending further evaluation to determine whether to exclude them from further vaccinations.**

- Acute disease at the time of entry into the study that in the opinion of the investigator may pose a threat to the subject
- Axillary temperature ≥ 37.5°C
- Respiratory rate ≥ 50 breaths per minute
- Serum ALT ≥ 50 IU/l (i.e., > 1.5 X ULN)
- Decreased renal function: serum creatinine levels > 92.2 mM/l (> 1.1 mg/dl)
- Significant anemia (Hgb < 8.0 g/dl)
- Thrombocytopenia (Platelets < 100,000 per mm^3^)
- Impaired immunity: (Absolute lymphocyte count [ALC] for 1 and 2 year olds < 2.5 x 10^3^/mm^3^; for 3 year‑olds < 2.0 x 10^3^/mm^3^)
- Homozygous sickle cell disease (Hgb SS)
- Malnutrition (Z score; Malnutrition = Weight for height < - 3 z-scores)
- Prior receipt of a rabies vaccine or any investigational vaccine
- Use of any investigational drug or vaccine other than the study vaccine within 30 days preceding the first dose of study vaccine, or planned use up to 30 days after the third dose
- Administration of chronic (defined as more than 14 days) immunosuppressants or other immune-modifying drugs within six months of vaccination. (For corticosteroids, this will mean prednisone, or equivalent, greater than or equal to 0.5 mg/kg/day. Inhaled and topical steroids are allowed)
- Administration or anticipated administration of a vaccine not foreseen by the study protocol within 30 days of the first dose of vaccine(s) with the exception of tetanus toxoid
- Previous vaccination with a vaccine containing MPL or QS21 (e.g., RTS,S/AS02A)
- Any confirmed or suspected immunosuppressive or immunodeficient condition, including human immunodeficiency virus (HIV) infection. (No HIV test will be performed as part of this study.)
- History of allergic reactions or anaphylaxis to immunizations or to any vaccine components, such as eggs
- History of surgical splenectomy
- Administration of immunoglobulins, blood transfusions, or any other blood products within the six months preceding the first dose of study vaccine or planned administration during the study period
- Simultaneous participation in any other clinical trial
- Acute or chronic cardiovascular, pulmonary, hepatic, or renal condition that in the opinion of the PI, may increase the risk to the subject from participating in the study
- Any other condition or circumstance that in the opinion of the investigator may pose a threat to the subject

## Treatments Potentially Interfering with Vaccine-Induced Immunity

**The following criteria should be checked at each scheduled clinic visit. If any become applicable during the study, the subject will not be required to discontinue, but a separate analysis may be carried out excluding these individuals. See Section 12.2 for definitions of study cohorts and datasets to be evaluated.**

**Any routine immunization deferred as a result of participation in this study, will be administered expeditiously starting 30 days after last receipt of study vaccine.**

- Use of any investigational drug or vaccine other than the study vaccine during the study period
- Administration of chronic (defined as more than 14 days) immunosuppressants or other immune-modifying drugs within six months of vaccination. (For corticosteroids, this will mean prednisone, or equivalent, ≥ 0.5 mg/kg/day. Inhaled and topical steroids are allowed.)
- Administration of a vaccine not foreseen by the study protocol during the period starting from 30 days prior to first dose to 30 day after the third dose visit
- Administration of immunoglobulins or any blood products up to 30 days after the last dose of vaccine

## Contraindications to Vaccination

The occurrence, in any eligible study subject, of any event enumerated in Section 6.9.6 (“Dose Discontinuation in Individual Subjects”) represents a contraindication to further immunization of that subject; however, the parent/guardian of such a subject will be encouraged to continue participation in the safety and immunogenicity evaluation for the duration of the study.

## Indications for Deferral of Vaccination

The following adverse events constitute grounds for deferral of vaccine administration at a given point in time; if any one of these adverse events occurs at the time scheduled for vaccination, the subject may be vaccinated later, within the allowable time interval specified in the protocol (see Section 7.1.2), or may be permanently discontinued from further vaccination at the discretion of the investigator (the only withdrawals from follow-up, i.e., from the study proper, would be for withdrawal of consent). The subject must be followed until resolution of the event as with any adverse event (see Section 10.4). If the subject is discontinued from further vaccination, the parent/guardian will be encouraged to keep the subject in the study for the safety and immunogenicity evaluation for the duration of the study.

- Axillary temperature ≥ 37.5 °C or evidence of clinical malaria (see Section 7.3.2) at the time of vaccination will warrant deferral of immunization until fever and symptoms resolve.
- Any other condition that in the opinion of the investigator poses a threat to the individual if immunized or that may complicate interpretation of the safety of the vaccine following immunization.

Such subjects will be followed in the clinic until the symptoms resolve or the window for immunization expires. No further vaccination will be performed if the subject does not recover (axillary temperature < 37.5°C or lack of symptoms, as appropriate) within nine days of the originally scheduled vaccination date. The subject, however, will continue to be followed for safety and immunogenicity. **If the subject meets any of the above criteria for deferral on the day of first immunization the PI may elect to exclude the subject from further participation in the study.**

## Safety Plan

### Criteria for Temporary Suspension of Vaccination

If any of the following SAEs occurs, the PI will ensure the temporary suspension of vaccination for all study subjects, and the events will be reported as described in Section 10.5 and in SSP No. MAL036-14.

1) Death in any subject where the cause of death is judged to be related to study vaccine

2) The occurrence in any subject of an anaphylactic reaction to study vaccine

3) The occurrence in any subject of a life-threatening SAE which is judged to be causally related to the vaccine (see definition of “Yes” as regards causality in Section 10.3, pg. 77)

4) The occurrence of a non-life-threatening SAE which is judged to be causally related to the vaccine

5) The occurrence, in one or more subjects, of laboratory abnormalities judged by the PI to be potentially life-threatening and related to receipt of study vaccine

The following findings will mandate temporary suspension of additional doses:

1. In 20 (5%) or more children, occurrence of Grade 3 solicited systemic (not local) AEs judged related to the study vaccine, or
2. In 20 (5%) or more children occurrence of Grade 3 unsolicited AEs judged related to the study vaccine.

NB: a) and b) will come from pooled, blinded data.

### Safety Review Prior to Administration of Consecutive Doses

After each dose, the PI will provide the LMM with a report covering the seven-day post‑dose observation period. This report will detail all SAEs occurring within the seven day period in all vaccinated children, as well as all solicited AEs and all Grade 3 unsolicited AEs for the first 200 children vaccinated. The PI will provide a copy of these reports to the DSMB, MVI, GSK Biologicals, USAID, and SCI. For each event, a statement of causality (event considered related/unrelated to vaccination, according to the PI’s best judgment) will be included. For the first and second doses, the reports will be submitted no less than seven days before the upcoming dose.

Administration of consecutive doses will not proceed until both of the following have occurred:

1. The LMM has reviewed the available safety data (and has had the opportunity to discuss the data with the DSMB, if she feels it necessary), and
2. The PI has determined that none of the SAEs or events outlined in Section 6.9.1 has occurred.

### Procedure for Temporary Suspension of Vaccination

If the PI understands that any of the criteria cited in Section 6.9.1 has been met, he will immediately suspend all further vaccinations and disseminate a brief preliminary report, by electronic mail, within 24 hours, to the LMM, study sponsor, study partners, and the DSMB, notifying them of the suspension.

### Procedures for Resumption of Dosing Following Temporary Suspension of Vaccination

1) Upon determining the need to *temporarily suspend* further vaccinations, the PI will, within three working days, produce a full written report to the DSMB on the incident in question including as much relevant information as possible. Within five working days of receiving the report, the DSMB Chairperson will convene a meeting (via teleconference, videoconference, face-to-face, or a combination of these) with the full membership of the DSMB, plus the LMM, to review and discuss the clinical information, safety data, and general course of events leading to the suspension. During the DSMB’s deliberations, all study partners (the PI, the sponsor [USAMMDA], WRAIR, GSK Biologicals, MVI, SCI, and USAID) will maintain a “standby status” and be available to provide, on short notice, any required information or other input.

2) After due discussion, the DSMB will submit a “verbal” (telephonic or electronic mail) recommendation as to an appropriate course of action to the study sponsor and other “voting” study partners (the PI, WRAIR, GSK Biologicals, and MVI); this will be followed up with a written version of the recommendation within three working days of the meeting. (Copies will be maintained at the study site and with the study sponsor.) Upon receiving the verbal recommendation, the voting partners will decide on the best course of action to be taken. Consultative, or “observer”, study partners (SCI and USAID) will also contribute to this discussion. Unanimous agreement by all five voting partners will be required for resumption of vaccinations.

3) If the DSMB, LMM, and voting partners agree that vaccinations should be recommenced, the HSRRB and the KEMRI ERC will be promptly notified, and the study will go forward with the remaining vaccinations. If, however, the DSMB recommends non-resumption of vaccinations, but the voting partners agree that vaccinations should be recommenced, then the IRBs (PATH/HSPC, the HSRRB, the KEMRI ERC) and US FDA must be consulted and all must concur with this decision before vaccinations resumption.

### Procedures for Permanent Discontinuation of Vaccination

Any decision for a *permanent discontinuation* of further vaccinations will take the form of a formal notification to the study sponsor from the PI. Such notification will come only after due discussion among the voting and consultative study partners and the LMM (as described in Section 6.9.4), and might or might not follow a recommendation for permanent discontinuation from the DSMB. The formal notification will also be provided promptly to PATH/HSPC, the HSRRB, the KEMRI ERC, and the DSMB. Regardless of whether permanent discontinuation of vaccinations occurs for an individual or for the entire study population, safety follow-up (for the entire study duration) and crossover rabies vaccine administration (after study completion) will still be performed.

### Dose Discontinuation in Individual Subjects

Reasons for discontinuation of study vaccine administration in an individual subject include, but are not limited to, the following:

1. Anaphylactic reaction following administration of study vaccine
2. Significant reactions (e.g., Grade 3 AEs) following administration of study vaccine (including severe pain, severe swelling, severe headache, persistent high fever or other severe systemic or local reactions) that, in the opinion of the investigator or sponsor, should preclude further administration of study vaccine
3. The occurrence of an SAE (See Section 10.5.1) related to the study vaccine
4. Receipt of any investigative drug, investigative vaccine or immunosuppressive medication (inhaled or topical corticosteroids are allowed) within 28 days of study vaccine
5. The occurrence of other medical conditions, unrelated to study vaccine that in the opinion of the PI contraindicate administration of study vaccine
6. Moderate or severe unsolicited AEs (clinical events or laboratory tests) that are indicative of new and significant end organ dysfunction and that, in the opinion of the investigator or sponsor, should preclude further administration of study vaccine

### Safety Precautions for Study Personnel

Laboratory safety and blood-borne precautions for all USAMRU-K laboratories are delineated in the following USAMRU-K SOPs:

1) SOP NO. KCL 007: Bloodborne and Infectious Control

2) SOP NO. KCL 006: Hazard Communication and Chemical Hygiene

3) SOP NO. KCL 003: Finger-Prick for Blood Smear

4) SOP NO. KCL 002: Emergency Phlebotomy Safety Procedures

5) SOP NO. KCL 005: Laboratory Hazards and Safety Procedures

## Final and Addendum Efficacy and Safety Analyses and Reports

The “Final Efficacy and Safety Data Report” (FESDR) will reflect the fully blinded period of efficacy surveillance from about SD 71 to about SD 240 (specific days differ among subjects, but all subjects will be surveilled for 169 days = six [28 day] months). This is the formal “Efficacy Follow up Period” (EFP). The FESDR will address the study objectives (Section 5), endpoints (Section 12.1), and test null hypothesis (Section 5.4) for the effect of the test article during the EFP (six months). A cleaned EFP database will be submitted to SCI; SCI will query the data, lock the database, and ultimately distribute the FESDR to the DSMB and all study partners as soon as it is available. No interim analysis will be performed. .

After the completion of the EFP, the study will continue in a partially unblinded manner (for efficacy, immunogenicity, and SAEs) for another 4 months. This period of additional efficacy surveillance lasts from about SD 240 to about SD 364. At the end of this 4 ½ month partially unblinded phase, SCI will receive a cleaned database. Information from this database will be appended to the FESDR.

The FESDR will be conducted on final, cleaned data. It will include sections on intention-to-treat (ITT) analyses and will reflect safety and efficacy surveillance data from SD 0 to SD 240 ~~71).~~ The AEFP data will be analyzed on final, cleaned data and appended to the FESDR.

# CONDUCT OF THE STUDY

## General Study Aspects

### Screening and Enrollment Process

(See also SSP No. MAL036-01: Recruitment and Informed Consent Process, and SSP No. MAL036-02: Screening Day Procedures.)

Recruitment and screening will proceed until 400 subjects are enrolled. Non-coercive means of recruitment will be used according to existing U.S. Army regulations (viz., AR 70‑25 and AR 40-38). Community leaders (assistant chiefs and local village elders) will be formally briefed in their own language on the nature and purpose of the study. They will be allowed to ask questions freely of the PI or his designees. Parents/guardians of prospective subjects will view a videotaped briefing on the nature and purpose of the study and will then receive both oral and written explanations of the study, after which written informed consent will be obtained from at least one parent per subject. No question relating to health will be asked of parents/guardians or children prior to consenting. So that language and illiteracy will not be impediments to informed consent, all such briefings and explanations will be in Dholuo, the local language. All screening tests and procedures (see below) will be performed only after informed consent has been obtained from the parent or guardian.

A record will be prepared for each subject at the screening visit. Source document forms will also serve as the Case Report Forms (CRFs) for this study. The screening forms, which will be labeled with a subject identification (SID) number, will contain information about the subject's date of birth, medical history, physical examination findings, date of screening visit, whether the subject was enrolled, and (where applicable) reasons for exclusion from the study.

Parents of subjects will provide a medical history (with special attention to any history of recurrent infections to suggest immune suppression, previous history of splenectomy, and prior vaccine reactions). Subjects will also undergo physical examination and routine standard laboratory screening tests, which include complete blood count (CBC), creatinine, and ALT. Hemoglobinopathies (sickle cell disease/trait, G6PD deficiency, and alpha-thalassemia) will also be screened. Subjects may be screened one additional time if re-screening after clinical intervention is determined to be necessary (e.g., treatment for malaria). Subjects will be excluded from participation if they meet any of the exclusion criteria (Section 6.5). Subjects excluded from this study because of significant abnormalities will be referred to the local health center for evaluation and treatment. All screening tests will be completed within 45 days prior to entry into the study; laboratory tests may be conducted at other times during the course of the trial if the investigator judges it necessary for the safety of the subject. All subjects randomized into the study will be identified and followed by SID number. All screening and follow-up diagnostic laboratory studies will be performed at the WRP KC laboratory. Information gathered during screening (medical history, physical examination, and laboratory analysis) will be recorded on the CRFs.

A photograph will be taken of each child who is screened, along with the parent/guardian, and stored securely in a computer at WRP KC. The investigator will randomize (see also Section 9.4) 400 eligible subjects who fulfill all the inclusion criteria and none of the exclusion criteria. A picture ID card of each eligible child and parent/guardian will be created using the stored photo, and the ID provided to each parent/guardian. The card will include contact details for study staff to facilitate documentation of medical care received outside the study health facilities. Copies of these pictures will also be kept in the subjects’ records to aid the study staff in confirmation of the child’s identification for future visits. The photos of subjects who are determined ineligible to participate will be deleted from the computer. CRFs will be filled in for all subjects enrolled in the study, and all data collected during the study will be recorded on the CRFs.

### Vaccination Process

On each vaccination day, criteria for continued eligibility will be reviewed and reverified. A history-directed physical examination will be done and axillary temperature, pulse, blood pressure, respirations and baseline general symptom history will be recorded. Venous blood will be collected for laboratory analysis as detailed in Section 8.1.

After the identity of the subject is checked (by comparing the SID number and photograph in subject’s record with the parent/subject’s photo ID), subjects will be vaccinated by intramuscular injection into the left anterolateral thigh muscles unless a compelling reason for using the right anterolateral thigh is evident (See also SSP No. MAL036-04: Vaccination Day Procedures). All vaccinations will be done in a fashion that maintains double-blinding (See Section 9.5.1). The vaccination periods may be up to ten consecutive days, but every effort will be made to vaccinate all 400 children over seven days.

Although all the foregoing study days are provisional (circumstances may require delays of up to two weeks), vaccinations (200 test article recipients, 200 rabies vaccine recipients) will occur on or about Study Days 0 (Dose 1), 29 (Dose 2), and 57 (Dose 3).

### Post-immunization Evaluation Procedures

Immediately after immunization, each subject will proceed to the next station for assessment of local and systemic reactions. Each subject will be observed for at least 60 minutes post-vaccination. Appropriate medical treatment will be readily available in the unlikely case of an anaphylactic reaction following the administration of the vaccine (see Section 9.8.1 and SOP 501.07). Signs and symptoms (pain, swelling, fever [defined as axillary temperature ≥ 37.5°C], and irritability/ fussiness) will be solicited from subject and parent and then recorded on the CRF by an evaluator according to adverse events recording procedures as outlined in Section 10.1.3.

Subjects will complete a seven-day (day of vaccination, plus six subsequent days) follow-up after each vaccination (five visits: day of vaccination, plus days 1, 2, 3, and 6 post‑vaccination) at the WRP KC or one of three outlying follow-up centers (see Section 16.3.3). All AEs will be followed until resolution (see Section 10.4). For any solicited signs or symptoms that persist beyond the first six days post-vaccination, the subject will be followed daily until resolution of the adverse event. Anorexia and any unsolicited AEs will be followed until resolution or study conclusion at intervals determined appropriate by the PI. At each visit, the subjects will be evaluated by soliciting post‑vaccination signs and symptoms from the parent and by examination of the injection site. A physical exam will be performed on day 30 (Visit 6). Every effort will be made to ensure parental and subject compliance with visits. However, if a parent and subject do not appear for a scheduled clinic visit, a clinician or nurse will attempt to contact them on the same day. The staff member who contacts the subject will measure vital signs and collect information on any solicited or unsolicited symptoms for the previous 24 hours. If the clinician determines that the subject has experienced a serious adverse event (SAE), appropriate measures will be taken to notify the Local Medical Monitor (LMM), the DSMB, the IRBs, and the sponsor (see Section 10.5.2).

### Active and Passive Case Detection of Malaria

*Active case detection* is represented by 1) the 15 scheduled MBF/CBC collections at the clinic and 2) the 13 monthly field worker home visits (see Exhibit 7 and Section 7.2), “Detailed Description of Study Stages and Visits”). All these scheduled sample collections and home visits will be undertaken whether or not children are symptomatic, and will include checks of temperature. All children known to be parasitemic with symptoms, including those with fever (≥ 37.5°C) or history of fever will be documented as such and treated as medically appropriate on the same day. The malaria blood films (MBFs) of children who are afebrile and well at the clinic will be read according to a time schedule dictated by microscopist workload. Therefore, any asymptomatic, but parasitemic, children who do not have their MBFs read “in real time” will not receive antimalarial treatment. (It is anticipated that these will represent 80-90% of children during high-transmission months). During field worker visits to the subjects’ homes, any child found to be ill from any cause will be promptly transported by the field worker to the WRP KC for evaluation and appropriate medical management.

*Passive case detection* is represented by subjects’ accessibility to the 12 field stations, which are manned on a 24-hour basis by field workers, as described in Section 16.3.3. Passive case detection will be in effect from the day of screening to Study Day 0 (for all those screened), and from Study Day 0 to study end (for all those enrolled). Parents of subjects will be advised on a continuous basis to bring any ill subject to a field station for immediate transportation to the KC at any time, night or day. Field workers will have standing instructions to arrange transport to the KC for any subject with reported fever or history of fever in the past 24 hours. Thus, all such children will be evaluated with an acute MBF and CBC. Subjects will then be treated at the KC IAW Section 7.3.3 (for malaria), Section 6.3.1 (for other illnesses), clinic SOPs, and established clinic guidelines. (This facility and its staffing are described in detail in Section 7.3.)

At study start, parents will be advised to seek medical attention for subjects *only* from the WRP KC. The exception of emergency medical care when remote from Kombewa Division will be fully explained to the parents. If a subject receives medical attention elsewhere, the investigators will attempt to obtain copies of any medical records and will encourage the parent/guardian to seek care for the subject only from the study physicians in the future.

Procedures for identification of subjects upon arrival at WRP KC are described in SSPs No. MAL 036-02 and MAL 036-04.

### Field Workers: Activities and Competencies

Field workers will have undergone a 2- to 3-week training period during which they will be taught basic study procedures such as taking vital signs and collecting finger stick blood samples. They will be given additional didactic information regarding malaria and other general health concerns. At the end of this period, they will have been tested on their knowledge and skills. Only those who satisfactorily pass this test will participate in the study.

Throughout the study period, field workers will man 12 satellite field stations 24 hours a day, 7 days a week. These stations will be located within a one-mile radius of parents/subjects and will provide a point of contact with the WRP KC by way of a hand-held radio or cellular phone. These field stations are not intended to be “aid stations”; they will be capable of rendering only the most basic first aid measures prior to transport of a patient/subject to the WRP KC. If an ill subject is brought to a field station, the field worker on duty will be able to arrange transport to the WRP KC.

Throughout the study period, field workers will conduct monthly home visits to determine the location of parents/subjects and remind parents of scheduled visits. Field workers will perform their 13 monthly home visits in order to identify malaria cases.

Field worker supervisors will visit a sample of subjects periodically to ensure that field workers are indeed tracking and collecting the required information (IAW SOP No. 101.01: Fieldworker Evaluation).

### Study Follow-up Periods

The solicited AE follow-up periods are the seven days following each vaccination (vaccination day and post-vaccination days 1, 2, 3, and 6). The unsolicited AE follow-up periods run for 30 days (vaccination days and the subsequent 29 days). SAE reporting will continue throughout the duration of the study (12 months). During the EFP all SAEs must be reported to the ORP within 24 hours of occurrence if possible (see Section 10.5.2). After the conclusion of the EFP, SAEs will be reported within 30 days from the time of occurrence except for deaths or events judged to be causally related to vaccine administration, which must be reported within 24 hours. In the event that a child is treated outside the WRP KC or the NNPGH, copies of all available clinical records of the encounter will be obtained and placed in the subject’s record. If a study subject is admitted to NNPGH, a member of the clinical team will visit the subject at least once daily and report to the PI or designee. The PI will be in contact with the pediatrician providing care to the subject and will ensure that the locally appropriate medical care is provided.

The efficacy follow-up period (EFP: surveillance for clinical malaria cases, parasite densities, and immunogenicity measurement) runs from study day 71 (fourteen days after the third vaccine dose) until Study Day 240(i.e., for 6 months).

The addendum efficacy follow-up period (AEFP: surveillance the same parameters as for the EFP) commences with the end of the EFP and continues until Study Day 364 (i.e., for 4 months).

Both the FESDR and the AESDR will include separate sections on intention-to-treat (ITT) analysis, which will reflect efficacy surveillance data beginning at the first vaccination (SD 0).

On study day 57 (the third vaccine dose), a period of twice monthly subject visits commences. These follow-up visits consist of monthly field worker home visits alternating with monthly WRP KC visits, such that surveillance visits of one kind or another occur every two weeks.

Exhibit 5. Mal-036 AE, SAE and 2 Efficacy Follow-up (F/U) Periods

| **Month** | **1** | | **2** | **3** | | **4** | | **5** | | **6** | **7** | | **8** | **9** | | | **10** | **11** | **12** |
| --- | --- | --- | --- | --- | --- | --- | --- | --- | --- | --- | --- | --- | --- | --- | --- | --- | --- | --- | --- |
| Study Day | 0-30 | | 31-60 | 61-90 | | 91-120 | | 121-150 | | 151-180 | 181-210 | | 211-240 | 241-270 | | | 271-300 | 301-330 | 331-364 |
|  | V | | V | V | |  | |  | |  |  | |  |  | | |  |  |  |
|  | F/U Period for Unsolicited AEs | | | | | |  |  | |  |  | |  |  | | |  |  |  |
|  | SAE F/U Period (ITT follow-up period) | | | | | | | | | | | | | | | | | | |
|  |  |  | | | Efficacy Follow-up Period (EFP)*  (Fully double-blinded) | | | | | | | | | |  |  | |  |  |
|  |  |  | |  | |  | | |  |  |  |  | | | Addendum EFP*  (Partially unblinded) | | | | |
|  | Monthly Field Worker Home Visits  (Staggered against monthly WRP KC Visits) | | | | | | | | | | | | | | | | | | |
|  |  |  | | Monthly WRP KC Visits  (Staggered against monthly FW Visits) | | | | | | | | | | | | | | | |
|  | ITT Analysis Period  (Passive Case Detection) | | | | | | | | | | | | | | | | | | |

V = 7 day F/U for Solicited AEs following each vaccination

* For malaria cases, parasite densities, genotyping and immunogenicity

## Detailed Description of Study Stages and Visits

The following schedule shows the visits for a single subject. Each event/day indicated on this schedule reflects a range of 7-10 days (or up to 25 days if the desired number of subjects has not been reached by the 10^th^ day) during which these events occur for the entire study population. Any death, out-migration, absence from Kombewa, and antimalarial usage will be elicited and documented for every scheduled clinic or home visit from Study Day 0.

**Days –45 to 0** **Screening of Subjects**

***Visit 1***

- Verification of written Informed Parental Consent
- Assignment of Subject Identification (SID) number
- Record axillary temperature, blood pressure, pulse, respirations
- Provision of medical history by subject’s parent/guardian
- Health assessment and physical examination (includes physical exam of all major body systems: ENT, pulmonary, cardiovascular, musculoskeletal, central nervous, renal, gastrointestinal and skin). Abnormal findings will be recorded in the subject’s record.
- Check of all inclusion and exclusion criteria
- Check weight
- Collect 2 ml venous blood sample for measurement of:
- Complete blood count (CBC), creatinine, ALT
- MBF
- Sickle cell, G6PD deficiency, and alpha-thalassemia
- 3D7 allotyping
- Provide each parent with SID card containing subject’s unique study number and parent/subject photograph. The photograph will be taken only of eligible (pending laboratory results) subjects who agree to participate and have been consented. It may be taken on any day within this period prior to the first vaccination

**Day 0 Enrollment, Randomization, and Vaccination 1**

***Visit 2***

Before vaccination:

- Check subject’s SID card to confirm identity
- Record axillary temperature, blood pressure, pulse, respirations
- Review screening laboratory test results
- Review inclusion/exclusion criteria and check of contraindications/ precautions
- Record any parental or subject complaints, interim history, history-directed physical examination and examination of the immunization leg for any abnormalities
- Review concomitant medications
- Record baseline data for solicited general symptoms
- Collect 3 ml of whole venous blood for the measurement of:
- CBC, creatinine, ALT
- Serum for anti-MSP-1 antibody titer
- 3D7 allotyping
- MBF
- Enrollment/Randomization
- Confirm that the subject’s randomization number and SID number agree with label on syringe
- Administer study vaccine dose 1; record site, date and time of injection.

After vaccination:

- Observe for 60 minutes.
- Record blood pressure, pulse, axillary temperature at the end of 60 minute observation period
- Record site of injection examination
- Record solicited and unsolicited AEs
- Instruct parent(s) to return with subject to WRP Kombewa Clinic immediately should subject manifest any sign or symptom perceived as serious.

**Days 1-3 Daily Post- vaccination 1 follow-up visits**

***Visits 3-5***

- Record pulse, axillary temperature
- Examine site of injection
- Record solicited and unsolicited AEs
- History-directed general physical examination
- Check concomitant medications

**Day 6 ± 3 Day 6 Post- vaccination 1 follow-up visit**

***Visit 6***

- Record pulse, axillary temperature
- Examine site of injection
- Record solicited and unsolicited AEs
- History-directed general physical examination
- Check concomitant medications

**Day 29 ± 9 days Vaccination 2**

***Visit 7*** Before vaccination:

- Check SID card to confirm subject identity
- Confirm that vaccination dose 1 was administered
- Record axillary temperature [must be <37.5 C], blood pressure, pulse, respirations
- Review inclusion/exclusion criteria and check of contraindications/ precautions
- Review day 0 laboratory test results
- Record any parental or subject complaints, interim history, history-directed physical examination and examination of the immunization leg for any abnormalities
- Review concomitant medications
- Record baseline data for solicited general symptoms.
- Collect 3 ml of whole venous blood for the measurement of:
- CBC, creatinine, ALT
- Serum for anti-MSP-1 antibody titer
- 3D7 allotyping
- MBF
- Confirm that the subject’s randomization number and SID number agree with label on syringe
- Administer study vaccine dose 2; record site, date and time of injection.

After vaccination:

- Observe for 60 minutes.
- Record blood pressure, pulse, axillary temperature at the end of 60 minute observation period
- Record site of injection examination
- Record solicited and unsolicited AEs
- Instruct parent(s) to return with subject to WRP Kombewa Clinic immediately should subject manifest any sign or symptom perceived as serious

**Days 30-32 Daily Post-vaccination 2 follow-up visits**

***Visits 8-10***

- Record pulse, axillary temperature
- Examine site of injection
- Record solicited and unsolicited AEs
- HIstory-directed physical examination
- Check concomitant medications

**Day 35 ± 3 Day 6 Post - vaccination 2 follow-up visit**

***Visit 11***

- Record pulse, axillary temperature
- Examine site of injection
- Record solicited and unsolicited AEs
- HIstory-directed general physical examination
- Check concomitant medications

**Day 57 ± 9 days Vaccination 3**

***Visit 12***

Before vaccination:

- Check SID card to confirm subject identity
- Confirm that vaccination dose 1 and 2 were administered
- Record axillary temperature [must be <37.5 C], blood pressure, pulse, respirations
- Review inclusion/exclusion criteria and check of contraindications/ precautions
- Review day 29 laboratory test results
- Record any parental or subject complaints, interim history, history-directed physical examination and examination of the immunization leg for any abnormalities
- Review concomitant medications
- Record baseline data for solicited general symptoms.
- Collect 3 ml of whole venous blood for the measurement of:
- CBC, creatinine, ALT
- Serum for anti-MSP-1 antibody titer
- 3D7 allotyping
- MBF
- Confirm that the subject’s randomization number and SID number agree with label on syringe
- Administer study vaccine dose 3; record site, date and time of injection.

After vaccination:

- Observe for 60 minutes.
- Record blood pressure, pulse, axillary temperature at the end of 60 minute observation period
- Record site of injection examination
- Record solicited and unsolicited AEs
- Instruct parent(s) to return with subject to WRP Kombewa Clinic immediately should subject manifest any sign or symptom perceived as serious.

**BEGIN EFFICACY FOLLOW-UP PERIOD 14 DAYS AFTER THIS VISIT.**

**Days 58-60 Daily Post-vaccination 3 follow-up visits**

***Visits 13-15***

- Record pulse, axillary temperature
- Examine site of injection
- Record solicited and unsolicited AEs
- History-directed physical examination
- Check concomitant medications

**Day 63 ± 3 Day 6 Post- vaccination 3 follow-up visit**

***Visit 16***

- Record pulse, axillary temperature
- Examine site of injection
- Record solicited and unsolicited AEs
- History-directed general physical examination
- Check concomitant medications

**Day 85 ± 10 Day 30 Post-vaccination 3 follow-up**

***Visit 17***

- Record pulse, axillary temperature
- Interim medical history (including any history of fever in last 24 hours)
- History-directed physical examination
- Record any unsolicited adverse events occurring after the last vaccine dose
- Check concomitant medications
- Collect 3 ml of blood for determination of:
- Serum for anti-MSP-1 antibodies
- 3D7 allotyping
- CBC, creatinine, ALT
- MBF

**Days 14 to 354 ± 7 Field Worker Home Visits**

***Field Worker Visits 1-13***

- Monthly field worker home visits will fall o/a Study days 14, 43, 71, 99, 129, 158, 186, 214, 242, 270, 298, 326, and 354 (all ± 7)
- Field workers will take axillary temperatures, solicit history of fever in the last 24 hours, remind parents of appointments and reconfirm their locations
- Any subject with a fever or history of fever in the past 24 hours will be taken to the KC for evaluation, including MBF and CBC.
- Any deaths, out-migrations, absences from Kombewa, and antimalarial usage will be documented
- Parents/subjects will continue to attend the WRP KC whenever the latter are sick
- Check concomitant medications

**Days 115 to 364 ± 14 WRP KC Visits**

***Visits 18-27***

- Monthly WRP KC visits will fall o/a Study days 115, 143, 171, 200, 228, 256, 284, 310, 340, and 364 (all ± 14)
- Beginning on study day 115, parents/subjects will visit the WRP KC monthly ± 14 days for interim history, including history of fever in 24 hours, axillary temperature, and 1 ml blood sample collection for MBF, CBC, and 3D7 allotyping
- On Study days 171, 256, and 340, an additional 1 ml of blood will be collected from which serum for anti-MSP-1 antibody titers will be aliquoted
- Check concomitant medications
- History-directed physical exam
- Parents/subjects will continue to attend the WRP KC whenever the latter are sick
- Any deaths, out-migrations, absences from Kombewa, and antimalarial usage will be documented

Exhibit 6. Intervals Between Study Visits

| **Interval** | **Size of Interval** |
| --- | --- |
| ***(Visit 1****→****Visit 2)***  ***(Visit 2****→****Visit 6)*** | ***Variable***  ***6 ± 9 days*** |
| ***(Visit 2****→****Visit 7)*** | ***28 ± 9 days*** |
| ***(Visit 7****→****Visit 11)*** | ***6 ± 9 days*** |
| ***(Visit 7****→****Visit 12)*** | ***28 ± 9 days*** |
| ***(Visit 12****→****Visit 16)*** | ***6 ± 9 days*** |
| ***(Visit 12****→****Visit 17)*** | ***29 ± 7 days*** |
| ***(Visit 18****→****Visit 27)*** | ***29 ± 7 days*** |

Exhibit 7. Schedule^a^ of Routine Subject Evaluations

| Clinic Visit | 1 | 2 | 3-6 | 7 | 8-11 | 12 | 13-16 | 17 | 18, 19 | 20 | 21, 22 | 23 | 24, 25 | 26 | 27 | FW 1-13 |
| --- | --- | --- | --- | --- | --- | --- | --- | --- | --- | --- | --- | --- | --- | --- | --- | --- |
| **Study Day** | **Scr** | **0** | **1,2,3,6** | **29** | **30, 31, 32, 35** | **57** | **58, 59, 60, 63** | **85** | **115, 143** | **171** | **200,228** | **256** | **284, 310** | **340** | **364** | **14-354** |
| Written Informed Consent | x |  |  |  |  |  |  |  |  |  |  |  |  |  |  |  |
| Verification of Eligibility Criteria | x | x |  | x |  | x |  |  |  |  |  |  |  |  |  |  |
| Medical History^k^ | x |  |  |  |  |  |  |  |  |  |  |  |  |  |  |  |
| Weight | x |  |  |  |  |  |  |  |  |  |  |  |  |  |  |  |
| Respirations | x | x |  | x |  | x |  |  |  |  |  |  |  |  |  |  |
| Assign SID | x |  |  |  |  |  |  |  |  |  |  |  |  |  |  |  |
| Check SID |  | x |  | x |  | x |  |  |  |  |  |  |  |  |  |  |
| Interim Medical History |  | x | x | x | x | x | x | x | x | x | x | x | x | x | x |  |
| Physical Examination | x |  |  |  |  |  |  |  |  |  |  |  |  |  |  |  |
| History-directed PE |  | x | x | x | x | x | x | x | x | x | x | x | x | x | x |  |
| Review labs |  | x |  | x |  | x |  | x | x | x | x | x | x | x | x |  |
| Examine injection site |  | x | x | x | x | x | x |  |  |  |  |  |  |  |  |  |
| CBC^d^ | x^b^ | x^c^ |  | x^c^ |  | x^c^ |  | x | x | x | x | x | x | x | x | x^m^ |
| Creatinine, ALT | x^b^ | x^c^ |  | x^c^ |  | x^c^ |  |  |  |  |  |  |  |  |  |  |
| Check Hemoglobinopathies | x |  |  |  |  |  |  |  |  |  |  |  |  |  |  |  |
| Serum for MSP-1 Ab Responses and GIA |  | x^c^ |  | x^c^ |  | x^c^ |  | x |  | x^e^ |  | x^e^ |  | x^e^ |  |  |
| 3D7 Allotyping | x | x |  | x |  | x |  | x | x | x | x | x | x | x | x | x^m^ |
| Malaria Blood Film | x | x |  | x |  | x |  | x | x | x | x | x | x | x | x | x^m^ |
| Axillary Temperature | x | x | x | x | x | x | x | x | x | x | x | x | x | x | x | x |
| Blood Pressure | x | x^f^ |  | x^f^ |  | x^f^ |  |  |  |  |  |  |  |  |  |  |
| Pulse | x | x | x |  | x |  | x | x |  |  |  |  |  |  |  |  |
| Study Vaccine Administration |  | x |  | x |  | x |  |  |  |  |  |  |  |  |  |  |
| Check Contraindications / Precautions |  | x |  | x |  | x |  |  |  |  |  |  |  |  |  |  |
| Assess Adverse Events^g^ |  | x | x | x | x | x | x | x |  |  |  |  |  |  |  |  |
| *(continued)* | | | | | | | | | | | | | | | | |

Exhibit 7. Schedulea of Routine Subject Evaluations (continued)

| Clinic Visit | 1 | 2 | 3-6 | 7 | 8-11 | 12 | 13-16 | 17 | 18, 19 | 20 | 21, 22, | 23 | 24, 25 | 26 | 27 | FW 1-13 |
| --- | --- | --- | --- | --- | --- | --- | --- | --- | --- | --- | --- | --- | --- | --- | --- | --- |
| **Study Day** | **Scr** | **0** | **1,2,3,6** | **29** | **30, 31, 32, 35** | **57** | **58, 59, 60, 63** | **85** | **115, 143** | **171** | **200,228** | **256** | **284, 310** | **340** | **364** | **14-354** |
| Assess Serious Adverse Events |  | x | x | x | x | x | x | x | x | x | x | x | x | x | x | x |
| Concomitant Medications^h^ | x | x | x | x | x | x | x | x | x | x | x | x | x | x | x | x |
| Monthly FW home visits^i^ |  |  |  |  |  |  |  |  |  |  |  |  |  |  |  | x |
| Hx fever in previous 24 hrs |  |  |  |  |  |  |  | x | x | x | x | x | x | x | x | x |
| Monthly WRP KC visits |  |  |  |  |  |  |  |  | x | x | x | x | x | x | x |  |
| Record baseline covariates^n^ | x |  |  |  |  |  |  |  |  |  |  |  |  |  |  |  |
| Exposure time variables^l^ |  | x | x | x | x | x | x | x | x | x | x | x | x | x | x | x |
| Scheduled blood volumes (ml) | 2 | 3 | 0 | 3 | 0 | 3 | 0 | 3 | 1 | 2 | 1 | 2 | 1 | 2 | 1 | 0 |
| **Cumulative** Blood Vol. (ml) | 2 | 5 | 5 | 8 | 8 | 11 | 11 | 14 | 16 | 18 | 20 | 22 | 24 | 26 | 27^j^ |  |

1. Days/dates for doses 2 and 3 are for planning purposes and are provisional only.
2. Performed within 45 days prior to randomization.
3. Blood collected just before immunization.
4. Limited to Hgb, WBC, RBC, lymphocytes, platelets.
5. Serum for MSP-1 antibodies collected on Study Days 171, 256, and 340.
6. Pre-dose and 60 min after each dose.
7. Solicited symptoms collected on Study Days 0, 1, 2, 3, and 6 after each dose. Unsolicited AEs collected from SD 0 through 85.
8. Concomitant medications collected from Study day -7 through 364.
9. Monthly field worker visits to checkaxillary temps, history of fever, and location and general status of subjects.
10. Volume does not include that taken for repeat screenings (if necessary) or for diagnostic samples in case of illness.
11. Includes history of malaria prevention methods used by the subject.
12. Includes death, out-migration, absences from Kombewa, and antimalarial drug usage.
13. Collected if temp ≥ 37.5°C, or if history of fever in previous 24 hours.
14. Age, sex, bed net use, nearest field site station, Hgb genotype.

## Definition and Management of Symptomatic and Asymptomatic Malaria

### Definitions of Symptomatic and Asymptomatic Malaria for Research Purposes

The following exhibit describes the definitions of clinical episodes of malaria (symptomatic cases) and asymptomatic malaria this study will use. The definitions of symptomatic malaria are listed in decreasing order of specificity. Note that the Primary Case Definition (temperature ≥37.5ºC in the simultaneous presence of a density of asexual stage *P. falciparum* ≥ 50,000 parasites/μl blood) is the third most specific of the definitions. We have chosen it as the primary endpoint because it is quite specific and we expect enough cases to occur under that definition to yield adequate power for the study.

All of the case definitions may present in this study through either active or passive case detection.

| Exhibit 8. Malaria Case Definitions | | | |
| --- | --- | --- | --- |
| **Definitions of Clinical Cases of Malaria** | | | |
| ***Name*** | ***Fever^a^*** |  | ***Parasite density^b^*** |
| Secondary Case Definition (200) | ≥37.5ºC | AND^c^ | ≥200K |
| Secondary Case Definition (100) | ≥37.5ºC | AND | ≥100K |
| **Primary Case Definition** | **≥37.5ºC** | **AND** | **≥50K** |
| Secondary Case Definition (10) | ≥37.5ºC | AND | ≥10K |
| Secondary Case Definition (0) | ≥37.5ºC | AND | >0 |
| Secondary Case Definition (0^*^) | ≥37.5ºC OR history of fever in the last 24 hrs | AND | >0 |

| **Definitions of Asymptomatic Malaria** | | |
| --- | --- | --- |
| ***Name*** | ***Description*** | ***Parasite density^b^*** |
| Infection Definition | Episode of malaria infection | >0 |
| Infection above 50K | Parasite density > 50K | ≥50K |

1. Fever is documented axillary temperature.
2. Asexual stage *P. falciparum* parasites/μl blood; K denotes thousands.
3. AND means “simultaneous presence of.”

### Definition of Symptomatic Malaria for Clinical Purposes

In the event that a subject presents to the WRP KC with fever (≥ 37.5ºC), history of fever during the past 24 hours, or any complaints that the attending clinician suspects are due to malarial infection, an MBF will be taken and read immediately to confirm the diagnosis, and a CBC will be performed. All subjects with asexual parasitemia will then be treated with antimalarials as indicated in Section 7.3.3.

Subjects found to meet any of the above criteria through active case detection will be evaluated by MBF and treated the same day, if appropriate.

### Management of Symptomatic Malaria during the Study

In April 2004, the Kenyan Ministry of Health announced artemether/lumefantrine (*Coartem***/***Riamet*®) as the new treatment-of-choice of non-severe malaria in all age groups; the new MoH guidelines are in preparation. *Coartem*® will be used for treatment of children who acquire uncomplicated malaria during the study. This drug has been shown to be safe and effective in children in Africa (35). Artemether/ lumefantrine will be administered as a six-dose regimen over 60 hours in accordance with the scheme outlined in Exhibit 9. This is the dosage regimen the WHO recommends for use in malaria endemic areas such as Kenya. The six-dose regimen has been found to be efficacious in Kenyan children under five years old (36).

Exhibit 9. Artemether/Lumefantrine (*Coartem*/*Riamet*®) Dosing Schedule

| **Body weight (Kg)** | **Number of tablets* at 0, 8, 24, 36, 48, and 60 hours** |
| --- | --- |
| 5 to 14.9 | 1 |
| 15 to 24.9 | 2 |
| 25 to 35 | 3 |

*Each tablet contains 20 mg of artemether and 120 mg of lumefantrine

In addition to artemether/lumefantrine, and in the unlikely event of treatment failure with that drug combination, the following regimen will also be available at the WRP KC as an alternative for the treatment of uncomplicated malaria:

- *Malarone* Pediatric Tablets (atovaquone 62.5 mg and proguanil Hcl 25 mg) dosed according to the body weight schedule presented in Exhibit 10.

**Exhibit 10.** ***Malarone* Dosage for Treatment of Acute Uncomplicated Malaria in Pediatric Patients**

| **Weight (kg)** | ***Malarone* Pediatric Tablets**: **Total Daily Dosage** | **Dosage Regimen** |
| --- | --- | --- |
| 11-20 | 250 mg/100 mg | 4 *Malarone* Pediatric Tablets as a single dose daily for 3 consecutive days |
| 21-30 | 500 mg/200 mg | 8 *Malarone* Pediatric Tablets as a single dose daily for 3 consecutive days |
| 31-40 | 750 mg/300 mg | 12 *Malarone* Pediatric Tablets as a single dose daily for 3 consecutive days |
| >40 | 1 g/400 mg | 16 *Malarone* Pediatric Tablets as a single dose daily for 3 consecutive days |

For subjects of < 11 kg body mass, the following regimen will also be available at the WRP KC as an alternative for the treatment of uncomplicated malaria:

- Quinine: Standard oral course of quinine 10 mg/kg t.i.d. for 7 to 10 days.

Children with manifestations of severe malaria (see Section 16.6; Appendix F) will be treated with parenteral quinine, 15 mg/kg loading dose followed by 10 mg/kg, q12 hours. Intravenous quinine will be administered in 5% dextrose. Other antimalarials (e.g. IM artemether) may be used at the physician investigator’s discretion should clinical circumstances dictate. Subjects with uncomplicated malaria and persistent vomiting, particularly those who are observed to not retain oral medication, should receive parenteral antimalarials. All subjects requiring parenteral treatment will be treated under observation at Kombewa Clinic or admitted to a referral hospital. At the investigator’s discretion, definitive clinical management may need to be rendered at the New Nyanza Provincial General Hospital (NNPGH), Kisumu, Kenya. A clinical member of the study team will review any such subject admitted to NNPGH each day until discharge. (See SOP 501.02: “Referral of Study Subjects at the WRP Kombewa Clinic for Medical or Dental Care”.)

All study subjects treated for malaria will be followed (at the clinic or by field worker home visit) until resolution of symptoms. All treatment courses will be administered under the direction of the attending clinician and recorded in the subject’s record. Subjects found to have clinical malaria will be treated before any further immunization. Subjects evaluated in this manner will be given the appropriate dose of vaccine if their clinical symptoms resolve within the vaccination window. If the clinical symptoms do not resolve within that period, the subject will not be vaccinated; however, the subject will be followed for collection of all study data points.

# SAMPLE HANDLING and ANALYSIS

## Overview of Collection Time Points

Blood will be collected from subjects for study purposes by venipuncture up to 15 times during the study. The maximum amount of blood requested from any subject for standard collection over the study duration should not exceed 30 ml. Additional blood may be obtained as deemed necessary by one of the investigators or clinicians to evaluate any illness or condition. Specimens collected during the study will be stored under the conditions specified within this protocol at either the laboratories within WRP-Kisumu or the WRAIR Department of Immunology. Only the investigators, laboratory managers and their staff will be permitted access to the specimens.

***Safety***

Testing for CBC, creatinine, and ALT at Visit #1 (Screening Period: Days -45 to 0) and at Visits # 2, 7, 12, and 17 (approximately Study Days 0, 29, 57 and 85).

Testing for hemoglobinopathies (sickle cell disease/trait, G6PD deficiency and alpha-thalassemia) will be performed at Visit 1 (Screening Period: Days -45 to 0).

***Malaria Blood Films (MBFs)***

MBFs for baseline and efficacy evaluation of parasite densities will be performed, commencing on screening days, continuing on vaccination days (Visits 2, 7, 12 o/a Study Days 0, 29, and 57), and every month thereafter, at the WRP KC. (For specific monthly dates, see Section 7.2).

The procedures for MBFs are outlined in the following WRP KC SOPs:

SOP KCL 301: Malaria Blood Films – Blood Sample Reception and Smear Preparation

SOP KCL 302: Preparation of Buffer Solution

SOP KCL 303: Preparation of Working Giemsa and Staining

SOP KCL 304: Slide Coordination and MBF Results Interpretation

SOP KCL 305: Malaria Blood Films – Reading and Quantification of Malaria Parasites

SOP KCL 306: Malaria Blood Films – Documentation and Storage of MBFs

SOP KCL 307: Malaria Blood Films – Quality Control and Quality Assurance

SOP KCL 003: Finger-Prick for blood Smear

SOP KCL 008: Processing of Laboratory Blood Specimens

SOP KCL 012: pH meter Calibration

SOP KCL 017: Microscope Maintenance

***Serology***

Separation of serum/plasma from the venous blood will be performed at the WRP KC and serum samples (approximately 0.5-1.0 ml) will be aliquoted for later use to determine anti-MSP-1 antibody levels at Visits 2, 7, 12, and 17 (approximately Study Days 0, 29, 57 and 85) and every three months (Visits 20, 23, 26) thereafter until the conclusion of the study.

## Handling of Biological Samples Collected by the Investigator

### Instructions for Handling of Serum Samples

*Collection*

Venous whole blood will be collected under appropriate aseptic conditions. Serum will be collected whenever possible using Vacutainer® tubes with integrated serum separator (e.g., Becton-Dickinson *Vacutainer*®SST or *Corvac*® Sherwood Medical). The blood will be mixed immediately by inverting the tube five times in order to mix the blood with the clot activator.

*Serum Separation*

- The clot will be allowed to set for 45 to 60 minutes at room temperature. Thereafter, the tubes will be centrifuged at 1500 g for 10 minutes.
- Following separation, the serum will be transferred to the appropriate standard screw top cryovials using a disposable pipette. The serum will be transferred as gently as possible to avoid blood cell contamination.
- The cryovials will not be overfilled (max. 3/4 of the total volume) to allow room for expansion upon freezing.
- The tube will be identified by an appropriate cryo-resistant label.

### Labeling

- Standard cryolabels will be used to label each serum or blood sample. In addition to a barcode, each label will contain the KEMRI SSC number, WRAIR protocol number, the SID number, and the date of collection.
- Any hand-written additions to the labels must be made using indelible ink.
- To ensure optimal attachment of the label, it should be attached to the tube as follows:
  - First attach the blank end of the label to the tube
  - Wrap the label around the tube so that the opposite end of the label overlaps the blank end ensuring that no written portion is covered
- Labels should not be attached to caps.
- The tubes of serum should be stored in a vertical position at a temperature ≤ -50°C.

## Laboratory Procedures

The PI and laboratory officer will maintain detailed SOPs governing laboratory assays in the WRP Kombewa Clinic laboratory. The following overview summarizes the methods that will be used.

### Hematology

Complete blood counts (CBCs) will be measured at regular intervals throughout the study period. The PI and WRP KC senior lab supervisor will ensure performance of laboratory reference value checks and will provide documentation to the study monitor before study start.

### Biochemistry

Renal (creatinine) and liver (ALT) function tests will be measured at regular intervals throughout the study safety period. The PI and WRP KC senior lab supervisor will ensure performance of laboratory reference value checks and will provide documentation to the study monitor before study start.

### Hemoglobinopathies

Assays for detection of sickle cell disease (SS) or trait (SA), G6PD deficiency, and alpha-thalassemia will be run at screening. The PI and WRP KC senior lab supervisor will ensure performance of laboratory reference value checks and will provide documentation to the study monitor before study start.

### Procedure for the Laboratory Diagnosis of Malaria

Routine MBFs will be collected at all scheduled blood draws. Whenever a child is suspected to have malaria, an acute MBF will be obtained and read immediately. Malaria parasite density will be estimated IAW SOP KCL 304 (See Section 16.3)

(Details for acute MBF preparation and reading are found in SOP KCL 305: Malaria Blood Films – Reading and Quantification of Parasites on Giemsa Stained Slides, Section E.)

### Serology: Antibody Responses assayed with ELISAs

Serological assays for antibody determination will be performed at the WRP-Kisumu laboratories and will undergo QC at the WRAIR. Blood will be collected at Visits 2, 7, 12, 17, 20, 23, and 26 (o/a Study Days 0, 29, 57, 85, 171, 256, and 340) from each subject and processed as detailed in Section 8.2.1. All blood samples will be labeled with the KEMRI SSC number, WRAIR protocol number, the SID number and the date the sample was obtained.

Immunogenicity (antibody levels) will be determined by evaluating antibody (IgG) responses to the *P. falciparum* MSP-1_42_ as measured using standard ELISA methodologies with appropriate capture antigens. (Details of this ELISA procedure are found in SOP No. KSM 201.1: SOP for Validation and QC of 3D7 MSP-42 Human ELISA and SOP No. KSM 202.0: SOP for Test Samples of 3D7 MSP-42 Human ELISA.)

*i. ELISA to measure immunogenicity against MSP-1_42_ (3D7):*

Primary analysis will be performed at USAMRU-K in Kenya, in the laboratory of Dr. John Waitumbi.

Secondary analysis will be performed at the WRAIR, in the Malaria Serology Laboratory, to ensure quality assurance/quality control, under the direction of Dr. Ann V. Stewart.

For the analysis of MSP‑1_42_-specific antibody titers, an ELISA will be performed according to an established and validated standard operating procedure. Induction of humoral immunity will be measured using MSP‑1_42_ (3D7) (Lot # 0649) as the capture antigen. Pre-immune and immune sera following each vaccine dose will be analyzed for the induction of MSP‑1_42_-specific antibody titers. Antibodies will be measured following secondary antibody conjugate with anti-human IgG horseradish peroxidase (HRP) incubation with the HRP substrate ABTS. Absorbance will be measured after 60 minutes at 405 nm with a microplate reader (Molecular Devices, Spectra-max Plus 384 using SoftMax Pro software). The antibody titer will be calculated as the serum dilution that produced an absorbance of one optical density unit in the ELISA assay.

*ii. ELISAs to measure immunogenicity against fragments of MSP-1 (3D7, FVO, CAMP) (MSP-1_42_, MSP-1_19_, and MSP-1-Domain 2):*

Assay will be performed at the WRAIR, in the laboratory of Dr. Jeff Lyon and Dr. Evelina Angov.

The C-terminal fragment of MSP‑1_42_ is highly conserved and constrained with six disulfide bridges. The p19 fragment is organized into two tandem EGF-like domains designated "EGF-domain 1" and "EGF-domain 2". Previous data show that mAbs that inhibit parasite invasion in vitro and inhibit the secondary processing of MSP‑1_42_ bind to p19 on either EGF-domain 1 (mAb 12.8) or to an epitope formed through double-domain (p19) (mAb 12.10). Pre-immune and immune sera obtained following each vaccine dose will be analyzed for the induction of antibody specificities induced to fragments of MSP‑1 by ELISA using six capture antigens, either homologous with the 3D7 vaccine strain, or the heterologous FVO and CAMP strains: full-length *E. coli*- MSP‑1_42_, C-terminal MSP‑1_19_ (p19), and -EGF-like domain 2 (EGF2). For the analyses comparing MSP‑1_42_-fragment-specific antibody titers, ELISA wells will be coated with equimolar concentrations of each antigen. Following incubation with immune sera, antibody titers will be measured using anti-human IgG, (H+L)-alkaline phosphatase (AP) conjugate followed by development with AP substrates, p-nitrophenol phosphate (Sigma) in diethanolamine. Absorbance will be measured after 30 minutes at 405 nm using a Molecular Devices, Spectra-max Plus 384 using SoftMax Pro software. The antibody titer will be calculated as the serum dilution that produced an absorbance of 1 optical density unit in the ELISA assay.

*iii. Competition ELISAs:*

Assay will be performed at WRAIR, in the laboratory of Dr. Jeff Lyon and Dr. Evelina Angov.

This assay has been previously described (38).

Immune sera from volunteers vaccinated with FMP1/AS02A will be evaluated to determine their ability to compete for binding to recombinant MSP‑1_42_ using a panel of monoclonal antibodies (mAbs) having known functional antibody specificities, i.e. growth invasion/inhibition activities (i.e., mAbs 12.10, 12.8). The competition ELISA is based on the hypothesis that serum antibodies that share overlapping epitope specificities with mAbs bind close to the mAbs and therefore sterically interfere with mAb binding. The degree of competition is influenced by the relative affinities of the serum antibodies and the mAbs, the degree of overlap of epitopes, and the concentration of antibodies in the serum.

iv. Inhibition of MSP-1_42_ Secondary Processing:

Assay will be performed at the WRAIR, in the laboratory of Dr. Jeff Lyon and Dr. Evelina Angov.

This assay has been described previously (39).

A target of protective immunity is the induction of antibody specificities to inhibit secondary processing of MSP1 to a membrane-bound fragment (MSP-1_19_) and a soluble fragment (MSP-1_33_). Therefore, the secondary processing is a pre-requisite for merozoite invasion and antibodies that block this processing also inhibit invasion.

Merozoites released from the *P. falciparum* 3D7, FVO or CAMP strains will be isolated and prepared for evaluation with immune sera for their ability to inhibit the secondary processing of MSP-1_42_ to p19 and p33. The merozoite pellet is suspended in ice cold PBS (Ca^+2^ and Mg^+2^ free) containing leupeptin, antipain, and aprotinin at 10 mg/ml and TLCK at 100 μM and washed three times in this same buffer. The merozoites are suspended in the same buffer supplemented with 1 mM CaCl_2_ and 1 mM MgCl_2_, and distributed in aliquots of 2x10^9^ merozoites in 1.4 ml microfuge tubes and pelleted at 12,000 x g for 2 min. Pellets are suspended in 30 μl of ice cold PBS supplemented CaCl_2_, MgCl_2_ and the immune sera from vaccinees. Assay controls include: 1 mM PMSF (inhibits secondary processing); no addition (permits secondary processing to proceed); and no addition followed by immediate addition of SDS (time zero control). All samples are incubated in a water bath at 37^o^C for 1 hour. Reactions are stopped by addition of SDS, followed by electrophoresis and analysis by western blotting for production of p33. Processing is detected by reaction with biotinylated-rabbit p42 polyclonal sera.

### Growth Inhibition Assay (GIA)

Invasion/Growth Inhibition Assays (i.e., measurement of invasion inhibitory antibody responses by parasite-derived lactate dehyrodrogenase assay [pLDH]) will be performed at the WRAIR using the pLDH method.

This assay will be performed at the WRAIR, in the laboratories of Dr. Jeff Lyon, Dr. David Haynes, and Dr. Evelina Angov.

Sera will be evaluated for inhibition of malaria parasite invasion/growth by measuring lactate dehydrogenase levels in late trophozoite stage *P. falciparum* adapted from a method developed by Prudhomme and Sherman (40). 3D7, FVO and CAMP strain *P. falciparum* are synchronized by thermal cycling as described by Haynes and Moch and collected for use at the beginning of schizogony (41). Cultures are adjusted to 0.3% parasitemia with type A human erythrocytes and assays are conducted in a final hematocrit of 1%, with test serum concentrations ranging from 5 to 20%. Assays with 3D7, FVO, and CAMP strains of *P. falciparum* will be collected 40 hours or 46 hours, after initiation. Parasite invasion inhibition is calculated as follows:

% Inhibition = [(OD_immune serum_ − OD_RBC_) ÷ (OD_pre-immune serum_ − OD_RBC_)] × 100

### Genotyping

Anticoagulated whole blood will be collected in heparin or EDTA as per schedule detailed in Exhibit 7 for purposes of genotyping the molecular allele of MSP-1 and determination of multiplicity of infections. Approximately 200 µl whole blood will be collected and processed for DNA by Qiagen whole blood method as described by manufacturer. DNA will be stored at –70˚C until used for molecular analysis.

*i. MSP-1_42_ Genotyping:*

Anti-coagulated EDTA blood collected at the indicated time points in Exhibit 7 from each subject will be used for DNA preparation. 200 μl of whole blood from each subject will used to prepare DNA using QIAamp DNA Mini Kit (Valentia, CA). For back-up, 20 μl whole blood will be spotted on *Isocode Stix* (Schleicher and Schuell, Dassel, Germany) in quadruplicate and stored at WRP-Kisumu. The DNA and the spotted blood will be shared on-a-need-basis between WRP-Kisumu and the WRAIR. A single primer set that amplifies a common genetic region in the MSP-1 gene from both the 3D7 (MAD20) allele and the FVO (K1) allele will be performed prior to real-time PCR detection of individual single nucleotide polymorphisms (SNPS). Each DNA sample from each time point will be assessed for the frequency of single or multiple SNPs for each of the eight polymorphisms. Four SNP assays have been developed for the 3D7 allele (vaccine allele), namely the 1) 3D7-33 SNP; 2) E allele; 3) T allele; 4) SR allele. Four SNP assays have been developed for the FVO (K1) allele, namely the 1) FVO-33 SNP; 2) Q allele; 3) K allele; 4) NG allele. As the technology currently stands (2^nd^ quarter 2004) the assay is both sensitive and specific for detection of presence/absence (frequency) of each MSP-1_42_ allele. Assays (real-time PCR, sequencing) that quantitatively measure the proportion of each allele in a sample are under development and may be utilized to quantitatively determine changes in abundance of MSP-1 alleles after vaccination.

*ii. Multiplicity of Infection Genotyping:*

To determine how many different infectious strains of *P. falciparum* the child was infected with, a molecular genotyping method that makes use of a PCR amplification method followed by RFLP (restriction fragment length polymorphism) will be used. Standard methods that measure the frequencies of three *P. falciparum* genes (MSP-1, MSP‑2, GLURP) will be analyzed for determination of multiplicity of infection. This approach will assess whether the FMP1/AS02A malaria vaccine helps to limit the number of infectious strains of malaria with which a child is infected. Further, it will allow us to measure the fitness/vulnerability of these parasite subpopulations, which are present in areas of high transmission.

### Optional Immunological Readouts

Results from these optional tests will not be encoded into the final database but will be analyzed and summarized separately by WRP or WRAIR. Immunofluorescence (IFA) titers against whole merozoites may also be measured in subjects both pre- and post-immunization; standard procedures will be followed. A subject will be considered a seroconverter by IFA if, two weeks after the second dose of vaccine, the subject develops an antibody titer (as measured by the highest IFA titer that gives a positive reaction) that is greater than four times that of the pre-immune serum. IFA assessments will be performed in either the Department of Immunology, WRAIR or at WRP Kisumu.

Any serum not immediately used in antibody assays will be stored indefinitely at ≤ ‑ 50°C and may be used in additional *in vitro* tests which may be useful in optimizing the follow-up of study subjects or in evaluating the immune response to the test vaccine preparation in these subjects. If this assay is not specified in this protocol, approval will be requested from the pertinent IRB(s) should the need for that assay arise.

### Other Studies

Long-term storage of sera will be undertaken so that other laboratory assays or tests, unanticipated at the present time, may be done in the future. Any such proposal for future testing, however, must be submitted for approval to the KEMRI ERC (Parents will be asked to sign a consent form to allow their child’s blood to be stored for future research purposes.)

# STUDY VACCINES and VACCINE ADMINISTRATION

## Study Vaccines

### FMP1/AS02A Vaccine

*Active Ingredients: FMP1 Antigen*

The FMP1 antigen is manufactured by the WRAIR Bioproduction Facility in Silver Spring, Maryland. The lot of formulated FMP1 to be used in this trial [#1034] consists of vials of lyophilized material prepared from volumes of 600 μl each. This includes the excipients (cryoprotectants) Tween 80 (0.2%) and lactose (3.15%), as well as the FMP1 antigen itself (approximately 61.8 μg of lyophilized protein per 600 μl vial or 103 μg/ml of the formulated vaccine).

Each vial of FMP1 will be labeled with the product name, manufacturer name and address, vial contents, lot number, date of manufacture, storage instructions and the caution: “New Drug Limited by Federal Law to Investigational Use Only”.

*Active Ingredients: AS02A Adjuvant*

The AS02A adjuvant is manufactured by GSK Biologicals in Rixensart, Belgium. AS02A contains 50 µg MPL, 50 µg QS21, 250 µl of SB62 (oil/water emulsion) in phosphate buffered saline (PBS) per volume of 0.5 ml AS02A pre-filled syringes stored at 2°C to 8°C.

Each syringe of AS02A will be labeled with the GSK study identifier, product name, manufacturer name and address, lot number, expiration date, storage instructions and the caution: “Limited by Federal (USA) Law to Investigational Use”.

*Reconstitution of Vaccine with Adjuvant*

Each vial of FMP1 antigen (approximately 61.3 μg) will be reconstituted with the complete contents of one AS02A pre-filled syringe. Thus, the injection of 0.5 ml will deliver an FMP1 dose of about 53.0 µg.

### Rabies Vaccine

The *Rabipur*®/*RabAvert*® [PCEC = purified chick embryo cell] rabies vaccine, manufactured in India by Chiron Behring Vaccines Pvt, Ltd, is a sterile, stable, freeze-dried lyophilisate of inactivated rabies virus prepared from the strain flury LEP. Each 1.0 ml dose of reconstituted vaccine contains the rabies antigen with polygeline, salts, and sugars. The antibiotics amphotericin B, chlortetracycline, and neomycin sulfate, which are used during the cell and virus propagation steps, are extensively removed by purification steps and therefore cannot be detected by currently available methods. The potency of the final product as determined by the NIH mouse potency test using the US reference standard is > 2.5 IU. The vaccine is supplied as single dose vials containing lyophilized antigen with 1.0 ml of diluent (sterile water for injection) in a snap-off ampoule and one sterile, disposable syringe with needle.

The *Imovax* ® [HDCV = human diploid cell culture vaccine] rabies vaccine, manufactured by Aventis Pasteur, SA, is a sterile, stable, freeze-dried suspension of rabies virus prepared from the strain PM-1503-3M, obtained from the Wistar Institute, Philadelphia, PA. Each 1.0 ml dose of reconstituted vaccine contains 100 mg of human albumin, < 150 μg of neomycin sulfate and equal or greater than 2.5 IU of rabies antigen. The potency of the final product is determined by the NIH mouse potency test using the US reference standard. The vaccine is supplied as single dose vials containing lyophilized antigen with 1.0 ml of diluent in a pre-filled syringe (sterile water for injection).

## Vaccine Dosage and Administration

### Preparation of FMP1/AS02A Vaccine

The top of a lyophilized FMP1 antigen vial will be disinfected with alcohol swabs and allowed to dry for a few seconds. The complete contents of one syringe pre-filled with AS02A will be injected using a sterile needle into a vial of lyophilized antigen. The needle will be discarded and the used syringe retained for accountability purposes. The pellet of FMP1 will then be dissolved by gently swirling the vial and waiting for about 1 minute to ensure complete dissolution of vial contents before withdrawing 0.50 ml (for a dose 53.0 µg) of reconstituted FMP1/AS02A into a sterile syringe with a 1” 23 gauge needle. The used vial will be retained for accountability purposes. (See also Section 9.5.1 for instructions on concealment of the syringe contents.)

### Reconstitution of Rabies vaccine

The top of a vaccine vial of rabies vaccine will be disinfected with alcohol swabs and allowed to dry for a few seconds. The complete contents of a syringe containing diluent (1.0 ml of water for injection) will be injected into a vial of lyophilized vaccine. The pellet will then be allowed to dissolve by gently swirling the vial and waiting for about 1 minute to ensure complete dissolution of vial contents before withdrawing about 1.0 ml of the reconstituted rabies vaccine into a sterile syringe with a needle. (See also Section 9.5.1 for instructions on concealing the syringe contents.) Once the single-dose vial has been penetrated, the withdrawn vaccine will be used promptly, and the vial will be retained for accountability purposes.

### Administration of Vaccines

Each 0.50 ml (53.0 µg) dose of FMP1/AS02A and each 1.0 ml (2.5 IU) dose of rabies vaccine will be administered by slow intramuscular injection into the left anterolateral thigh muscle. This is the recommended route and site for rabies administration in neonates, infants, and young children. In the case of FMP1/AS02A, injection will be done immediately after reconstitution. (“Immediately” in this context implies not more than several minutes.)

Intravenous, subcutaneous, and intradermal routes of administration will not be used. Aspiration will be performed prior to injection to avoid injection into a blood vessel. If a vessel is inadvertently penetrated, the needle will be withdrawn and reinjected at a slightly different site. As an alternative site of immunization, the right anterolateral thigh muscle will be used when injection at the left injection site is contraindicated or not advisable, such as in the case of severe pain, infection or if the subject or parent voices a preference to be immunized in the alternative site. A separate syringe and needle will always be used for each subject receiving either vaccine. In no case (whether FMP1/AS02A or rabies) will more than one vaccine dose be obtained from a single vial. The vaccines will be visually inspected for particulate matter and discoloration prior to administration if solutions and containers permit. Once the single-dose vial has been penetrated, the withdrawn vaccine will be used promptly, and the used vial will be retained for purposes of accountability.

Vaccines will be administered at the WRP KC under the supervision of clinicians skilled in the management of anaphylactic reactions. The vaccinees will be observed closely for at least 60 minutes, with appropriate medical treatment readily available in the unlikely event of anaphylactic reaction following the administration of the vaccine (See SOP No. 501.07: Treatment of Anaphylaxis in Children). In order to maintain the study blind, the team of clinicians tasked to administer the vaccine will be different from those involved in the evaluation of subjects following immunization.

## Vaccine Storage

# THE FMP1 ANTIGEN, AS02A SYRINGES, AND RABIES VACCINE MUST BE STORED UNDER REFRIGERATION (+ 2°C to + 8°C) AND MUST NOT BE FROZEN. The refrigerator designated for storing these products will have a 24-hour temperature recording. A back-up refrigerator and generator will be available in case of breakdown/power failure. The refrigerator will be kept locked; keys to it will only be routinely possessed by the designated drug manager, the clinic pharmacist, or the deputy clinic director for administration. Records will document receipt, temperature during transit, release for immunization, disposal, or return to the manufacturer for all vaccine vials. Copies of these records will be provided to the sponsor for archiving. (See also SOP No. 301.03: Vaccine and/or Drug Refrigerator Monitoring and Maintenance, SOP No. 301.04: Vaccine and/or Drug Refrigerator Failure, and SSP No. MAL036-10: Vaccine Storage and Packaging for Transport.)

## Randomization of Vaccine Allocation

Subjects will be randomized to one of two study arms at the time of first vaccination. One arm will receive FMP1/AS02A vaccine (200 subjects) and the other arm will receive rabies vaccine (200 subjects). The study will use blocked randomization, but will not be stratified by age or gender. Subjects will be blocked sequentially in order of enrollment. Block size will not be disclosed by the study statistician until after completion of the study in order to maintain study blinding. It is not expected that this will result in an important imbalance in the age or gender distribution between the test and comparison groups.

The randomization list will contain sequential codes linked to a study vaccine assignment (FMP1/AS02A or rabies vaccine). The codes will be assigned to subjects in the order in which they present to the clinic on the first day of immunization. The only persons at the study site with access to the randomization list are the study drug manager (or designee), the clinic pharmacist and the pharmacist’s assistant(s); therefore, these individuals will be unblinded. It is essential that these unblinded individuals understand the importance of not revealing the contents to anyone else involved in the study. The LMM will also keep one set of the randomization codes in a sealed envelope in if emergency unblinding is necessary.

## Method of Blinding and Breaking the Study Blind

### Blinding

The study will be double-blinded: both study participants and those investigators responsible for evaluation of the endpoints will be blinded as to who receives the test article versus the comparator. (See Glossary for definitions of full and partial blinding.) On vaccination days, the comparator vaccine will be in the same package as received from the manufacturer. After agitation it will appear clear and pink. The FMP1 antigen and the AS02A adjuvant will be packaged separately. The reconstituted FMP1 antigen in the AS02A adjuvant/diluent will have a milky white appearance. Because the vaccines will have a markedly different appearance, contents of the syringe will be concealed as described later in this section. The two vaccine preparation teams, consisting of the study pharmacist, pharmacy assistants, and drug manager (an experienced nurse, clinician, or pharmacist), will be responsible for vaccine preparation. They will also verify that the proper vaccine and vaccine dose is prepared and delivered to each subject. To determine which vaccine each subject will receive, the drug manager will refer to the unique randomization code assigned to that subject (See Section 9.4). The drug manager will check the subject number on the parent/subject’s photo ID and will confirm that it matches the SID in the subject’s record. The drug manager will then refer to a key matching the randomization code given to the subject to the vaccine to be administered. The study pharmacist and assistants will also confirm that the randomization code of the subject matches the vaccine to be given in the key list. The vials will be prepared (rabies or FMP1/AS02A), as in Sections 9.2.1 and 9.2.2, and the appropriate volumes will be drawn into syringes. Each syringe will be labeled with a sticker containing the randomization code for the individual and the individual’s SID number. The contents of the syringe will be sealed with a masking tape. The nurses or clinicians assigned the task of immunizing will be different from those assigned to do post-immunization assessment.

Immunization will be carried out simultaneously in 2 to 4 different exam/consultation rooms, which are connected to the vaccine preparation room by small, closable service hatches. The hatches will remain closed at all times except to exchange records and the pre‑filled syringe for immunization. The vaccine-filled syringes will be handed through this window to the vaccinators. The vaccinators will be medically qualified personnel (*e.g*., a clinician or nurse) with experience in IM injection of vaccines. For each subject, the SID number, the randomization code from the chart, and the randomization code on the syringe containing the vaccine will be recorded on the vaccination CRF. (For more details, see SSP No. MAL036-09: Vaccine Preparation and Administration).

### Breaking the Study Blind

A subject's study randomization code may be unblinded only for safety purposes. Such exceptional procedures should be discussed by the sponsor, the PI, the LMM, and the DSMB. If deemed necessary for reasons such as safety, the LMM or the Drug Manager (in Kenya) or SCI (in the US) will unblind a specific subject without revealing (unless clinically indicated) the study blind to the investigators. Any opening of these coded envelopes will be well documented.

The PI and Kenya field team members will remain blinded to all individual study data until the completion of the study (SD 364; for details, see Section 6.10 and Glossary). The study will remain fully blinded until the distribution of the FESDR; thereafter, it will continue in a partially unblinded fashion for the remainder of the study duration. Full unblinding will occur after study day 364, upon release of the appended study information. If a study code becomes known to the PI prior to the study’s partial unblinding (upon distribution of the FESDR), the PI must notify the sponsor immediately and document the reasons for unblinding in the study record.

## Replacement of Unusable Vaccine Doses

In addition to study vaccines, additional single doses of FMP1, AS02A and rabies vaccine will be provided to replace broken or lost doses. All the procedures described for study vaccines apply.

## Vaccine Accountability

The study vaccines and diluents will be kept in a locked refrigerator at either the WRP KC or the WPR Kisumu Research Laboratory, both of which have 24-hour temperature recording capability (See SSP No. MAL036-10: Vaccine Storage and Packaging for Transport and Section 9.3 above). Movement of these items into and out of the site will be documented appropriately. In no case (whether FMP1/AS02A or rabies vaccine) will more than a single vaccine dose be obtained from a single vial. Used vaccine vials, as well as unusable vaccine vials, will be retained for vaccine accountability purposes until authorization from GSK (for AS02A) and the study sponsor (for FMP1) has been received to destroy or return these vials.

## Concurrent Medication/Treatment

At each study visit or other clinical encounter, an investigator or other clinician will question a subject’s parent about any concurrent medication taken by the subject.

Concurrent medications—including any vaccine other than the study vaccines, malaria prophylaxis, any blood product, and any medication relevant to the protocol, including any specifically contraindicated—administered during the period starting from one week before the first dose and continuing to study end must be recorded on the case report form with trade name or generic name of the medication, medical indication, start and end dates.

### Drugs for Treatment of Anaphylaxis

Drugs to treat anaphylaxis include epinephrine 1:1,000 and 1:10,000 (aqueous), diphenhydramine and hydrocortisone, all of which are readily available at the WRP KC. A kit with supplies necessary for airway management and oxygen will also be available on site. A clinician familiar with Pediatric Advanced Life Support (PALS) procedures will be present on-site during immunizations. The WRP Kombewa Clinic guideline is SOP No. 501.07: Treatment of Anaphylaxis in Children.

### Drugs to Treat Malaria

*Coartem*® (artemether/lumefantrine) will be the first‑line agent for the treatment of uncomplicated malaria. In addition, both *Malarone*® Pediatric Tablets (atovaquone 62.5 mg and proguanil Hcl 25 mg) and oral and parenteral quinine will be available on site at the WRP Kombewa Clinic as alternatives. These drugs will be used in standard doses and regimens as described in Section 7.3.3.

# ADVERSE EVENTS

The recording of adverse events is an important aspect of study documentation. The PI is responsible for ensuring the documentation of all adverse events according to the detailed guidelines below. The subjects’ parent(s) will be instructed to contact the investigator immediately should the subject manifest any sign or symptom perceived as serious.

## Eliciting and Documenting Adverse Events

### Definition of an Adverse Event

Reportable non-serious adverse events (RNAEs) are discussed in Section 10.1.4; serious adverse events (SAEs) are discussed in Section 10.5.

An adverse event (AE) includes any noxious, pathological, or unintended change in anatomical, physiological, or metabolic functions as indicated by physical signs, symptoms, or laboratory test abnormalities occurring in any phase of the clinical study whether associated with the study vaccine and whether considered vaccine-related. This includes an exacerbation of pre-existing conditions or events, intercurrent illnesses, a vaccine or drug interaction, or the significant worsening of a previously documented condition. A symptom present just prior to a first vaccination, and not exacerbated after the vaccination is administered, should not be considered an AE. Similarly, anticipated day-to-day fluctuations of pre-existing conditions, including the disease under study, that do not represent clinically significant exacerbations need not be considered AEs. However, discrete episodes of chronic conditions occurring during a study period (after the first vaccination) should be reported as AEs in order to assess changes in frequency or severity.

AEs should be documented in terms of a medical diagnosis. When this is not possible, an AE will be documented in terms of signs and symptoms observed by the investigator or reported by the participant at each study visit.

Pre-existing conditions, or signs and symptoms (including any that are not recognized at study entry but are recognized during the study period) that are present in a subject prior to the start of the study, should be recorded on the Medical History CRF. Any solicited sign or symptom present during a pre-vaccination assessment should be recorded on the Pre‑Vaccination Assessment CRF.

AEs that occur after informed consent is obtained, but prior to a first vaccination, will be documented on the Medical History CRF.

Any non-elective hospitalization will be considered an SAE, rather than an AE, regardless of the reason for hospitalization and possible causal relationship to vaccine administration (See Section 10.3).

Solicited AEs to be recorded are described in Section 10.1.5. Any other AEs will be recorded as unsolicited AEs.

### Surveillance Period for Occurrence of Adverse Events

All AEs occurring within one month (maximum 30 days) following administration of each dose of vaccine must be recorded on the Adverse Event CRF, irrespective of severity or likelihood of vaccination-relatedness.

### Recording of Adverse Events

At each visit, assessment, or encounter, any AEs not previously documented in the study will be recorded in the Adverse Event CRF. The nature of each event, date and time (if appropriate) of onset, outcome, intensity and relationship to vaccination will be established. Details of any corrective treatment should be recorded on the appropriate CRF. See Section 10.5.2 for instructions for reporting and recording of SAEs.

As a consistent method of soliciting AEs, the subject’s parent(s) will be asked a non‑leading question such as:

"Has the child acted normally and appeared to feel as usual?"

N.B. The investigator should record only those AEs having occurred within the time frames defined above, see Section 10.1.3.

AEs already documented on the CRF, (i.e., at a previous assessment), and designated as ‘ongoing’ should be reviewed at subsequent visits, as necessary. If these have resolved, the documentation on the CRF should be completed. If an AE changes in frequency or intensity during a study period, a new record of the event will be started.

Solicited AEs will be elicited for the first six days post immunization; unsolicited AEs will be recorded during a 30-day follow-up period (day of vaccination and 29 subsequent days).

Serious adverse event (SAE) reporting is detailed in Section 10.5.2.

### Reporting of Adverse Events

The PI will provide to the LMM reports detailing solicited AEs no less than seven days prior to a subsequent dose (i.e., provisionally on Study days 22 and 50). After reviewing each report, the LMM will, if she concurs, recommend proceeding with the next scheduled dose. This concurrence may be given informally (e.g., telephonically) but will be recorded by the PI in the study regulatory file. This report to the LMM will consist of the data on the first 200 children vaccinated at each dose. A complete report on data for all children vaccinated at a given dose will be presented to the LMM later. This will be followed by a complete report of all the children eligible for vaccination. For information purposes, the solicited AE reports will also be provided to the DSMB and study partners (GSK Biologicals, MVI, WRAIR and USAID).

A special category of AE describing events of particular concern to the PI, but falling short of the reporting criteria for an SAE, are herein defined as “reportable, non-serious adverse events” (RNAEs). RNAEs, while not life-threatening, incapacitating or requiring non-elective hospitalization, nevertheless elicit a level of concern to the PI such that he considers it imperative to detail them in an informal, but expedited, fashion to study partners. Examples are non-life-threatening allergic reactions, striking skin rashes, elective hospitalizations, cancer diagnoses, or other signs or symptoms with suspicious temporal relationships to study vaccinations. For the purposes of this study, RNAEs will be reported in simple memorandum format, rather than on a dedicated CRF.

### Solicited Adverse Events

Exhibit 11. Solicited Adverse Event (AE) Categories

| Categories | Adverse Events |
| --- | --- |
| Local (injection site) AEs | Pain at the injection site |
|  | Swelling at the injection site |
| General (systemic) AEs | Fever (i.e., axillary temperature ≥ 37.5°C) |
|  | Irritability/fussiness |
|  | Loss of appetite |
|  | Drowsiness |

N.B.: Axillary temperature will be recorded at the time of the clinic visit. Should additional temperature measurements be recorded at another time of day, the highest temperature will be recorded.

The assessment of severity/intensity will be as described in Section 10.2. For general signs and symptoms reported, the investigator will ensure that causality is assigned as described in Section 10.3.

For all signs and symptoms reported, the investigator will ensure that the outcome is reported as described in Section 10.4.

### Unsolicited Adverse Events

A CRF will be provided to record unsolicited AEs. Unsolicited AEs are signs or symptoms reported by the subjects or their parents within 30 days of a vaccination that are different from those solicited symptoms recorded during the seven-day post-vaccination follow‑up period. Signs and symptoms that would fit the solicited AE category, but actually start after the sixth day post-vaccination, will also be considered unsolicited AEs. An investigator will ensure that any general (systemic) signs or symptoms reported will be evaluated, and their relationship with the study vaccine determined and transcribed onto the CRF.

## Assessment of Intensity

Intensity of solicited AEs will be assessed and graded by an investigator or clinician as presented in Exhibit 12.

| Exhibit 12. Assessment of Solicited Adverse Event (AE) Intensity | | |
| --- | --- | --- |
| **AE** | **Grade** | **Intensity Definition** |
| Pain at injection site | 0 | Absent |
|  | 1 | Minor reaction to touch |
|  | 2 | Cries/protests on touch |
|  | 3 | Cries when limb is moved or spontaneously painful or limping or reluctance to bear weight |
| Swelling at injection site | 0 | Absent |
|  | 1 | < 5 mm |
|  | 2 | 5-20 mm |
|  | 3 | > 20 mm |
| Fever | 0 | < 37.5°C |
|  | 1 | 37.5-38.0°C |
|  | 2 | 38.1-39.0°C |
|  | 3 | > 39.0°C |
| Irritability/Fussiness | 0 | Behavior as usual |
|  | 1 | Crying more than usual/no effect on normal activity |
|  | 2 | Crying more than usual/ interferes with normal activity |
|  | 3 | Crying that cannot be comforted/ prevents normal activity |
| Drowsiness | 0 | Behavior as usual |
|  | 1 | Drowsiness easily tolerated |
|  | 2 | Drowsiness that interferes with normal activity |
|  | 3 | Drowsiness that prevents normal activity |
| Loss of appetite | 0 | Normal |
|  | 1 | Eating less than usual/no effect on normal activity |
|  | 2 | Eating less than usual/interferes with normal activity |
|  | 3 | Not eating at all |

For all other AEs, maximum intensity should be assigned to one of the following categories:

0 = No adverse event

1 = An adverse event that is easily tolerated by the subject, causing minimal discomfort and not interfering with everyday activities.

2 = An adverse event that is sufficiently discomforting to interfere with normal everyday activities.

3 = An adverse event that prevents normal, everyday activities. (Such an adverse event would, for example, prevent attendance at school, church or other typical assemblies, and would necessitate the administration of corrective therapy.)

## Assessment of Causality

For each AE, the investigators will ensure that a determination is made on the presence or absence of a causal relationship to the administration of the study vaccine.

The degree of certainty with which an AE can be attributed to administration of the study vaccine (or alternative causes, e.g., natural history of underlying diseases, concurrent therapy, etc.) will be determined by how well the event can be understood in terms of one or more of the following:

- Reactions of a similar nature having previously been observed with this type of vaccine or formulation.
- The event having often been reported in literature for similar types of vaccines.
- The event being temporally associated with vaccination or reproduced on re-vaccination.

All fevers occurring within 24 hours of a vaccination will be considered causally related to the vaccination unless a plausible concurrent illness is a more likely cause. All solicited local (injection site) reactions that occur within 24 hours of vaccination will be considered causally related to vaccination.

Assessment of causality in all other AEs should be documented by the investigators or clinicians by answering the following query on the Serious and Unexpected Adverse Event Report:

“In your opinion, did the vaccine(s) possibly contribute to the adverse event?”

NO: Either the adverse event is not causally related to administration of the study vaccine, or there are other, more likely causes and administration of the study vaccine is not

suspected to have contributed to the adverse event.

YES: There is a reasonable possibility that the vaccine contributed to the adverse event.

Non-serious and serious AEs will be evaluated as two distinct events given their different medical nature. The PI will investigate an event that meets the criteria to be determined “serious” (see Section 10.5.1 for definition of an SAE), to the extent that a determination of ALL relevant contributing factors can be made.

A partial list of possible contributing factors might include:

- Past medical history
- Concurrent medications
- Protocol required procedures
- Lack of efficacy of the test vaccine
- Erroneous vaccine administration

## Adverse Event Follow-up and Assessment of Outcome

Investigators should follow up subjects with SAEs until the event has subsided, or until the condition has stabilized, regardless of when this occurs in relation to the study conclusion. Investigators should follow up subjects with non-serious AEs until study conclusion for a given subject.

Only one of the following outcomes should be documented:

1 = Recovered without sequelae

2 = Recovered with sequelae

3 = Worsened (New AE)

4 = Died

5 = Unknown

And, if ongoing at the subject’s study conclusion,

6.1 = Ongoing and unchanged

6.2 = Ongoing, but improved

6.3 = Ongoing and worsened

## Serious Adverse Events

### Definition of a Serious Adverse Event

A serious adverse event (SAE) is any untoward medical occurrence that results in death, is *life-threatening*, results in persistent or significant *disability or incapacity*, requires in-patient *hospitalization* or prolongation of existing hospitalization, or is a congenital anomaly or birth defect in the offspring of a study subject. In addition, important medical events that may require intervention to prevent one of the other outcomes listed above should be considered serious. (Examples of such interventions include intensive treatment in an emergency room, out-patient clinic or at home for allergic bronchospasm; blood dyscrasias or non-febrile seizures that do not result in hospitalization; and development of drug dependency or drug abuse.)

Additionally, the following will always be reported as SAEs: (1) any clinical management involving use of parenteral antimalarials and (2) any subjects meeting the definition of severe falciparum malaria according to the 2000 World Health Organization criteria (see Section 16.6; Appendix F).

Although not considered to be SAEs, elective hospitalizations, pregnancies and cancer diagnoses should be reported in the same expedited fashion as SAEs. No pregnancies are anticipated in this pediatric study. They are categorized as a special type of AE: the reportable, non-serious adverse event (RNAE) (See Section 10.1.4).

Definitions of terms used in the above definition of an SAE are:

*Life-threatening*: An adverse event is life-threatening if the subject was at risk of death at the time of the event; it does not refer to an event that hypothetically might have caused death if it were more severe.

*Disabling or incapacitating*: An adverse event is incapacitating or disabling if the event results in a substantial disruption of the subject's ability to carry out normal life functions. This definition is not intended to include experiences of relatively minor medical significance such as headache, nausea, vomiting, diarrhea, influenza, and accidental trauma (*e.g*., sprained ankle).

*Hospitalization*: In general, hospitalization signifies that the subject has been detained (usually involving at least an overnight stay) at the hospital or emergency ward for treatment that would not have been appropriate in the physician’s office or other out-patient setting.

*Routine clinical procedure*: A procedure which is defined in the protocol as one that may take place during the study period and should not interfere with the study vaccine administration or any of the ongoing protocol specific procedures.

If anything untoward is reported during an elective procedure, that occurrence must be reported as an AE, either ‘serious’ or ‘non-serious’, according to the usual criteria.

When in doubt as to whether ‘hospitalization’ occurred or was necessary, the event should be considered an SAE.

It is the intent to capture and document 100% of the SAEs through the mechanisms detailed in Section 7.3 and Section 16.3.3 (in Appendix D). A subject’s parents will be instructed to contact the investigator at any time should the subject manifest signs or symptoms that they perceive as serious. They will be reminded to report to the nearest field station in the event of illness, so that transportation to the WRP KC can be arranged. They will be counseled to seek medical attention only through the WRP KC.

### Reporting Serious Adverse Events

Unanticipated problems involving risk to subjects or others, serious adverse events related to participation in the study and all subject deaths will be promptly reported by phone (301-619-2165), by email ([hsrrb@det.amedd.army.mil](mailto:hsrrb@det.amedd.army.mil)) or by facsimile (301-619-7803) to the Army Surgeon General’s Human Subjects Research Review Board (HSRRB). If not submitted with the initial notification, a complete written report will follow within 3 working days. In addition to the methods above, the complete report will be sent to the U.S. Army Medical Research and Materiel Command, ATTN: MCMR-ZB-QH, 504 Scott Street, Fort Detrick, Maryland 21701-5012.

During the first eight and a half months of the study (through Study Day 240), all SAEs, *whether or not they are judged to be related to the test article or comparator vaccine*, will be reported as detailed above, as well as to GSK Biologicals. In addition, the following organizations will receive copies of the written report, and so will be notified within three working days: KEMRI ERC, MVI/PATH, SCI, LMM, the DSMB, USAID, and WRAIR ORM.

After Study Day 240, SAEs will be reported monthly for the remainder of the study. Exceptions to this are: 1) any death in a study subject or 2) any SAE thought to be vaccine-related. These SAEs will be reported within 24 hours, as stated in the first two paragraphs of this section. The following organizations will receive copies of the monthly SAE reports: KEMRI ERC, HSRRB, MVI/PATH, USAMMDA, GSK Biologicals, SCI, LMM, DSMB, USAID, and WRAIR ORM.

Every SAE that is not resolved at the time the initial written report is filed will have a follow-up report submitted when information is available. Any submitted report will be identified as “initial”, “follow-up”, or “medical monitor” report.

An initial notification will include:

- Name of the PI
- Study protocol number
- Subject ID number, initials, age, and gender
- Date of onset of the event
- Date of the most recent administration of the study vaccine

The PI will not wait to collect additional information to fully document the event before making notification of an SAE. The telephone or email report should be followed by a full written report utilizing the Serious Adverse Events CRF, detailing relevant aspects of the SAEs in question.

Instances of death, cancer, or congenital abnormality in offspring, if brought to the attention of the investigator AT ANY TIME after cessation of the study, AND suspected by the investigator to be related to study vaccine, should be reported to the study sponsor.

Copies of all documents relating to SAE reporting will also be sent promptly by the PI to the study sponsor (POC: Dr. Moshe Shmuklarsky, USAMRMC, Ft Detrick, MD, USA). It is the sponsor’s responsibility to submit all SAE reports to the FDA.

Points of contact for the above mentioned individuals, committees, and institutions are given below.

**Human Subjects Research Review Board (HSRRB):**

The Surgeon General of the Army’s Human Subjects Research Review Board

COL Laura Brosch, Acting Chair, HSRRB

Human Subjects Protection Division

U.S. Army Medical Research and Materiel Command

Fort Detrick, MD 21702-5012

*Tel +301-619-2165/6*

*Fax +301-619-7803*

*Email* [*hsrrb@det.amedd.army.mil*](mailto:hsrrb@det.amedd.army.mil)

**WRAIR Human Use Review Committee (WRAIR HURC):**

Dr. Sara Rothman

Office of Research Management

WRAIR, Silver Spring, MD 20910

*Tel +301-219-9961*

*Fax +301-319-9940*

*Email* [*sara.rothman@na.amedd.army.mil*](mailto:sara.rothman@na.amedd.army.mil)

**WRP/KEMRI Ethics Review Committee (ERC):**

Dr. Monique K. Wasunna

Director, Centre for Clinical Research

Kenya Medical Institute of Research

Nairobi, Kenya

*Tel +254-20-722541*

*Email* [*mwasunna@nairobi.mimcom.net*](mailto:mwasunna@nairobi.mimcom.net)

**US Army Medical Materiel Development Activity (USAMMDA):**

Dr. Moshe Shmuklarsky

Biomedical PM Support Scientist

Military Infectious Diseases Research Program

USAMRMC ATTN: MCMR-ZB-DRI

504 Scott Street, Bld. 722, Room 8

Fort Detrick, MD 21702-5012

*Tel* *+ 301 619-7797*

*Fax + 301 619-2416*

*Email* [*Moshe.Shmuklarsky@amedd.army.mil*](mailto:Moshe.Shmuklarsky@amedd.army.mil)

**Malaria Vaccine Initiative (MVI):**

Ms. Lora Polowczuk

Technical Coordinator

Malaria Vaccine Initiative at PATH

*Tel +301 770-5377 x171*

*Fax +301 770-5322*

*Email* [*lpolowczuk@malariavaccine.org*](mailto:lpolowczuk@malariavaccine.org)

**US Agency for International Development**

Carter Diggs, MD, PhD

Senior Technical Advisor

*Tel +202-712-5728*

*Fax +202-216-3702*

Email [cdiggs@usaid.gov](mailto:cdiggs@usaid.gov)

**Local Medical Monitor (LMM):**

Juliana Otieno, MBChB, MMed, Peds

Chief, Dept of Pediatrics

New Nyanza Provincial General Hospital

PO Box 849

Kisumu, Kenya

*Cell +254-733-715917*

*Fax +254-57-41330*

*Email* [*JOtieno@kisian.mimcom.net*](mailto:JOtieno@kisian.mimcom.net)

Alternate:

James Gesami, MBChB, MMed

Nyanza Provincial Medical Headquarters
PO Box 721
Kisumu, Kenya *Tel +254-57-40091/41550
Fax +254-57-21870*

*Cell +254-733-826090*

*Email* [*pmonyanza@africaonline.co.ke*](mailto:pmonyanza@africaonline.co.ke)

**GlaxoSmithKline Biologicals (GSK Biologicals):**

Isabelle Ramboer, PhD

GSK Biologicals

*Tel +32-2-656-6820*

*Fax +32-2-656-8044*

*Mobile +32-495-846140*

*Email:* [*isabelle.ramboer@gskbio.com*](mailto:isabelle.ramboer@gskbio.com)

GSK Biologicals Safety Department

*Email* [*Rix.ct-safety-vac@gskbio.com*](mailto:Rix.ct-safety-vac@gskbio.com)

GSK Biologicals Clinical Safety Physician

GlaxoSmithKline Biologicals

Rue de l’Institut 89

1330 Rixensart Belgium

*Tel +32.2.656.87.98*

*Fax +32.2.656.80.09*

*Mobile phone for 24/7 day availability +32-477-40-47-13*

**Statistics Collaborative, Inc.:**

Kathryn Tucker, MS and Ms. Jennifer Wilson

1710 Rhode Island Avenue, NW, Ste. 200

Washington, DC 20036

*Tel +202-429-9267*

*Fax +202-429-9268*

*Email:* [*kathryn@statcollab.com*](mailto:kathryn@statcollab.com)

[*jennifer@statcollab.com*](mailto:jennifer@statcollab.com)

**Data and Safety Monitoring Board (DSMB):**

*DSMB Chairman:*

Professor Fred Binka

School of Public Health

University of Ghana

PO Box LG 13

Legon / Ghana

*Tel +233-21-500-388*

*Email* [*fbinka@africaonline.com.gh*](mailto:fbinka@africaonline.com.gh)

*Other DSMB members:*

Dr. Marcel Tanner

Professor and Director, Swiss Tropical Institute

Socinstrasse 57 PO Box 4002

Basel / Switzerland

*Tel +41-61-284-8283*

*Email* [*marcel.tanner@unibas.ch*](mailto:marcel.tanner@unibas.ch)

Dr. William Blackwelder

8613 Hempstead Avenue

Bethesda, MD 20817

*Tel +301-564-6137*

*Email* [*wcb@boo.net*](mailto:wcb@boo.net)

Dr. Clara Menendez

Unidad de Epidemiologia Hospital Clinica

Universidad de Barcelona

C/ Villarroel 170 E-08036

Barcelona Spain

*Tel +34-932-275-706*

*Email* [*menendez@clinic.ub.es*](mailto:menendez@clinic.ub.es)

Dr. Norbert Peshu

Centre for Geographic Medicine Research–Coast

Kenya Medical Research Institute

*Tel +254-125-22063*

*Fax +254-125-22390*

*Email* [*npeshu@kilifi.mimcom.net*](mailto:npeshu@kilifi.mimcom.net)

## Treatment of Adverse Events

Treatment of any AE is at the sole discretion of the PI or the LMM and should be in accord with accepted, local standards of good medical practice. The applied measures should be recorded in the subject record. The recording of AEs is an important aspect of study documentation. The PI is responsible for ensuring documentation of all AEs according to the detailed guidelines set out herein. The subjects’ parents will be instructed to contact the PI or his designees immediately should a subject manifest any sign or symptom perceived as serious.

# SUBJECT COMPLETION and DROP-OUT

## Definition of a Drop-out

From the perspective of data analysis, a 'drop-out' is any subject who is not brought back for the concluding visit foreseen in the protocol. Any subject who has not completed all three immunizations will, if the parent/guardian is willing, still be followed for the entire study period. Any such subject who is brought in for the concluding visit foreseen in the protocol is considered to have completed the study and is therefore not considered a dropout.

## Procedures for Handling Drop-outs

Investigators will ensure that attempts will be made to contact parents of those subjects who have not been brought in for scheduled visits or follow-up. If, however, a parent has withdrawn parental consent, and if he or she requests no further home follow-up visits, the investigators will honor that request and no further procedures will be required of the subject. (In that case, the parents will be advised to seek treatment in future at the Kombewa Sub-District Hospital or other local health facility.) The reason(s) for withdrawal will be solicited from the parents. This information will be described on the Study Completion CRF.

## Reasons for Drop-outs

The Study Completion CRF will specify which of the following possible reasons were responsible for drop-out of the subject from the study:

- - Withdrawal of parental consent, due to a serious adverse event (SAE)
  - Withdrawal of parental consent, due to an adverse event (AE)
  - Withdrawal of parental consent, not due to an AE or SAE
  - Protocol violation (Specify)
  - Migration of family and/or subject from the study area
  - Loss to follow-up, cause unknown
  - Withdrawal by PI, due to a serious adverse event (SAE)
  - Withdrawal by PI, due to an adverse event
  - Other (Specify)

# DATA MANAGEMENT and ANALYSIS

Data entry will be performed on site at the WRP Kombewa Clinic. The CRCs will remove duplicate CRF pages periodically after review and/or approval of the study monitors and will submit to the data entry center. Statistics Collaborative, Inc., Washington, DC, USA will perform data analysis and reporting.

## Study Endpoints

### Primary Endpoint

The primary endpoint is time to first clinical episode of *P. falciparum* malaria meeting the “Primary Case Definition” given in Section 7.3. Time is measured from 14 days after administration of the third dose of study vaccine (SD 71).

### Secondary Endpoints

Because this is a Phase IIb trial, it has many secondary endpoints. Their purpose is to gain insight into the efficacy of the vaccine and to help define a primary endpoint for a confirmatory Phase III trial. They are not intended for use to make formal claims about the vaccine.

Measurements of efficacy during the EFP:

- Times to clinical episode of *P. falciparum* malaria meeting the various secondary case definitions shown in Exhibit 8
- Time to presence of asexual stage *P. falciparum* parasites during the six-month efficacy follow-up period
- Time to parasite density ≥ 50,000 asexual stage *P. falciparum* parasites per microliter of blood during the six-month efficacy follow-up period
- Number of clinical episodes of malaria, per subject, meeting the primary case definition
- Presence of MSP-1 3D7 parasites in clinical cases of malaria meeting the primary case definition
- Presence of MSP-1 3D7 parasites at a given cross-section survey time
- Time to anemia defined as Hgb < 8.0 g/dl, whether by active or passive case detection
- Proportion of subjects with anemia (Hgb < 8.0 g/dl)

Safety during the EFP:

- Occurrences of solicited AEs during a seven‑day follow-up period (vaccination day and six subsequent days) after each vaccination (five visits: vaccination day and days 1, 2, 3, and 6)
- Occurrences of unsolicited AEs during a 30-day follow-up period after each vaccination (vaccination day and 29 subsequent days)
- Occurrences of SAEs between the first vaccination and the end of the EFP

Immunogenicity during the EFP:

- Geometric means of MSP-1 Ab titers at Visits 2, 7, 12, 17, 20, and 23 (o/a Study Days 0, 29, 57, 85, 171, and 256)

### Tertiary Endpoints

This study has many tertiary endpoints. As with the secondary endpoints, their purpose is to gain further insight into the performance of the vaccine.

Measurement of safety (during the EFP and Addendum Efficacy Follow-up Period [AEFP]):

- Occurrences of SAEs between the first vaccination and the end of the study

Measurement of immunogenicity (during the EFP and AEFP):

- Geometric means of MSP-1 Ab titers at Study Days 0, 29, 57, 85, 171, 256, and 340

Measurement of efficacy and other biological effects by either active or passive detection (during the AEFP unless otherwise noted):

- Times to first clinical episode of *P. falciparum* malaria meeting the primary case definition (EFP and AEFP combined)
- Times to first malaria episode of *P. falciparum* malaria meeting the secondary case definitions in Exhibit 8 (EFP and AEFP combined)
- Time to presence of asexual stage *P. falciparum* parasites (EFP and AEFP combined)
- Time to parasite density ≥ 50,000 asexual stage *P. falciparum* parasites per microliter of blood (EFP and AEFP combined)

Number of clinical *P. falciparum* malaria episodes, per subject, meeting the primary case definition (EFP and AEFP combined)

- Presence of MSP-1 3D7 parasites in clinical cases of malaria meeting the primary case definition (EFP and AEFP combined)
- Presence of MSP-1 3D7 parasites at a given cross-section survey time (EFP and AEFP combined)
- Vaccine-induced GIA activity
- The parasite density at each episode of malaria infection meeting the primary and secondary definitions of clinical malaria and infection (see Exhibit 8)
- *P. falciparum* malaria allele types per malaria episode, meeting the primary and secondary definitions of clinical malaria and infection (see Exhibit 8)
- Times to first observation of anemia (Hgb < 8.0 g/dl), whether by active or passive case detection (EFP and AEFP combined)
- Hgb levels at all scheduled points of collection
- Proportion of subjects with anemia (Hgb < 8.0 g/dl) at each study collection
- Proportion of subjects with a documented decline in Hgb level of ≥ 20%
- Proportion of subjects with anemia (Hgb < 8.0 g/dl) at last study collection
- Number of cases of documented decline in Hgb level from baseline to last study sample collection
- Parasite density at all scheduled points of collection
- Non-3D7 parasite densities at all scheduled points of collection
- Genotyping for multiplicity of infections
- Antibody against fragments of MSP-1_42_
- Antibody able to compete for binding to recombinant MSP-1_42_
- Antibody able to inhibit secondary processing of MSP-1_42_

## Study Cohorts and Data Sets to be Evaluated

### Total Cohort

For purposes of study data analysis, the “total cohort” will include all subjects enrolled in the study.

### Efficacy Cohort

For the purpose of study data analysis, two efficacy cohorts will be defined. The intention-to-treat (ITT) cohort will consist of all subjects who receive at least one dose of study vaccine or comparator vaccine. Given that children are to be randomized immediately before receiving their first dose of vaccine, the ITT and total cohort should differ by no more than a few children. For purposes of this ITT analysis, MBFs that are performed, read, and recorded between SD 0 and SD 71 inclusive (the first vaccination and 14 days after the third vaccination) will be archived and available despite falling outside the EFP and AEFP. The according-to-protocol (ATP) cohort will consist of subjects who receive all three doses of study vaccine or comparator vaccine, meet the inclusion/exclusion criteria, and do not have any important protocol violations.

### Safety Cohort

For purposes of study data analysis, the “safety cohort” will consist of all subjects who have received at least one dose of study vaccine or comparator vaccine and for whom any data on safety are available for analysis.

The presentations of safety data will explore separately the adverse experiences among subjects who received all three vaccinations and among those who received fewer; among those who received each of the three vaccine doses; and among those with clinically important violations of study protocol.

### Immunogenicity Cohort

For purposes of study data analysis, the “immunogenicity cohort” will include all subjects for whom assay results are available for antibodies against the specified study vaccine antigen component or the comparator. There are no standard cut‑off titers for seropositivity determinations in general use for the MSP-1 assay.

## Sample Size

Preliminary results from the WRAIR #1020 epidemiological study indicate that approximately 50% of children experienced at least one episode of clinical malaria in the first six months of follow-up. In this analysis, clinical malaria is defined as a parasite density ≥ 50,000 parasites per microliter in the presence of fever (axillary temperature ≥ 37.5° C). If one assumes that time to clinical malaria follows an exponential distribution, a 50% cumulative incidence rate corresponds to a hazard rate of 0.1155/month. A trial with 200 subjects per study arm, therefore, will have roughly 80% power to detect a 38% reduction in this hazard rate. A 38% reduction in the hazard rate corresponds to a 30% reduction in the six-month cumulative incidence rate. In terms of time to event, these rates imply that 25% of the subjects in the comparator group are expected to contract malaria within about 2.5 months from the third immunization; the comparable time for the FMP1/AS02A arm will be roughly 4 months. The median time to first clinical episode is expected to be 6 months and 10 months, respectively.

This sample size calculation is based on a two-sided 0.05-level logrank test. It assumes that children receive 3 doses of vaccine and then are followed for 6 months after the third dose. Cases of malaria are counted after the third dose of vaccine. Twenty percent of children are assumed to dropout during the immunization period. No further dropouts are assumed during the efficacy follow-up period.

## Final analyses

This section describes the analyses planned for the primary and secondary endpoints as well as for the most important of the tertiary endpoints. A Reporting and Analysis Plan (RAP) will be prepared before all subjects reach SD 240 and the study is partially unblinded. The RAP will describe the data analyses in fuller detail.

### Analysis of Demographics

Demographics characteristics such as age, gender, baseline bed net usage, and hemoglobin genotype will be tabulated by vaccine study arm. For continuous variables, means, standard deviations, and ranges will be calculated. For binary variables, frequencies and percentages will be computed.

### Analysis of Test Article Efficacy

The effect of FMP1/AS02A on clinical and biological endpoints will be analyzed as outlined below. For time-to-event analyses based on the six-month efficacy follow-up period, time is measured from 14 days after administration of the third dose of study vaccine (SD 71). Subjects will be censored at the time they become lost to follow-up or at the end of the efficacy follow-up period (SD 240), whichever occurs first. Loss to follow-up will also reflect death and moving away from the study area. Adjustments to time at risk will be made for anti-malarial usage and temporary absence from Kombewa. For time-to-event analyses based on the ten-month efficacy follow-up period, time will be measured from 14 days after administration of the third dose of vaccine. Subjects will be administratively censored at SD 364. For time-to-event-analyses based on the ITT cohort, time is measured from time of randomization (SD 0).

#### Clinical vaccine efficacy

***Primary endpoint***

*Time to first episode of clinical malaria during the six-month efficacy follow-up period using the primary case definition* – The logrank statistic and corresponding p‑value will be calculated to test the null hypothesis of no difference in the distribution of time to clinical malaria between the FMP1/AS02A and rabies vaccine groups. A Cox proportional hazards model with an indicator variable for vaccine group will be fit to the data and an estimate of the hazard ratio along with its corresponding 95% confidence interval will be computed. Vaccine efficacy will be defined as one minus the hazard ratio. Additional Cox models may explore the effects of age, sex, bed net use as reported at baseline, nearest field station, and hemoglobin genotype on vaccine efficacy. Kaplan-Meier curves for time to clinical malaria will be generated by vaccine group. An alternative estimate of vaccine efficacy based on cumulative incidence rates will be calculated as well.

The primary data analysis will be conducted with the ATP cohort; however, a similar analysis based on the ITT cohort will be performed to assess the robustness of the analysis based on the ATP cohort.

***Secondary endpoints***

*Time to first episode of clinical malaria during the six-month efficacy follow-up period using the secondary case definitions in Exhibit 8 –* Similar analyses as those outlined for the primary endpoint will be carried out for these endpoints.

*Number of clinical episodes of malaria per subject during the six-month efficacy follow-up period using the primary case definition* – Statistical methods for recurrent event data, such as Poisson regression, will be used to assess the effect of FMP1/AS02A vaccination on the average number of clinical episodes per subject. A table of the frequency of clinical episodes by vaccine group will be generated.

*Number of clinical episodes of malaria per subject during the six-month efficacy follow-up period using the secondary case definitions in Exhibit 8* – Similar analyses as those conducted for the number of episodes using the primary case definition will be carried out.

***Tertiary endpoints***

*Time to first episode of clinical malaria during the ten-month efficacy follow-up period using the primary case definition* – Similar analyses as those outlined for the primary endpoint will be carried out for this endpoint. In addition, Cox proportional hazards models with time-dependent covariates reflecting the various follow-up periods will be fitted to the data to assess whether vaccine efficacy changes with time.

*Time to first clinical episode of malaria during the ten-month efficacy follow-up period using the secondary case definitions* – Similar analyses as those outlined for the primary endpoint will be carried out for these endpoints. In addition, Cox proportional hazards models with time-dependent covariates reflecting the various follow-up periods will be fitted to the data to assess whether vaccine efficacy changes with time.

*Number of clinical episodes of malaria per subject during the ten-month efficacy follow-up period using the primary case definition* – Similar analyses as those proposed for number of episodes meeting the primary case definition during the six-month efficacy follow-up period will be conducted.

*Number of clinical episodes of malaria per subject during the ten-month efficacy follow-up period using the secondary case definitions* – Similar analyses as those proposed for the number of episodes meeting the primary case definition during the ten-month efficacy follow-up period will be conducted.

#### Biological effects

***Secondary endpoints***

*Time to presence of asexual stage P. falciparum parasites during the six-month efficacy follow-up period* – Similar analyses as those described for the primary endpoint will be performed.

*Time to parasite density ≥ 50,000 asexual stage P falciparum parasites per microliter of blood during the six-month efficacy follow-up period* – Similar analyses as those described for the primary endpoint will be performed.

*Presence of MSP-1 3D7 parasites in clinical cases of malaria meeting the primary case definition during the six-month efficacy follow-up period* – Frequencies and percentages of cases with circulating MSP-1 3D7 parasites will be calculated by vaccine study group.

*Presence of MSP-1 3D7 parasites at a given cross-section survey time during the six-month efficacy follow-up period* – For a given survey time, the frequency and percentage of subjects with circulating MSP-1 3D7 will be calculated by vaccine study arm.

*Time to anemia defined as hemoglobin level < 8.0 g/dl during the six-month efficacy follow-up period* - Similar analyses as those performed for the primary endpoint will be conducted. In addition, a Cox model with vaccine study arm, baseline hemoglobin, and age as covariates will be applied to the data. The six-month cumulative incidence rate of anemia will be calculated for each vaccine study arm.

***Tertiary endpoints***

*Time to presence of asexual stage P. falciparum parasites during the ten-month efficacy follow-up period* – Similar analyses as those described for the primary endpoint will be performed.

*Time to parasite density ≥ 50,000 asexual stage P falciparum parasites per microliter of blood during the ten-month efficacy follow-up period* – Similar analyses as those described for the primary endpoint will be performed.

*Presence of MSP-1 3D7 parasites in clinical cases of malaria meeting the primary case definition during the ten-month efficacy follow-up period* – Frequencies and percentages of cases with circulating MSP-1 3D7 parasites will be calculated by vaccine study group.

*Presence of MSP-1 3D7 parasites at a given cross-section survey time during the ten-month efficacy follow-up period* – For a given survey time, the frequency and percentage of subjects with circulating MSP-1 3D7 will be calculated by vaccine study arm.

*Time to anemia defined as hemoglobin level < 8.0 g/dl during the ten-month efficacy follow-up period* – Similar analyses as those performed for the primary endpoint will be conducted. In addition, a Cox model with vaccine study arm, baseline hemoglobin, and age as covariates will be applied to the data. The six-month cumulative incidence rate of anemia will be calculated for each vaccine study arm.

### Analysis of Immunogenicity

Antibody responses as measured by anti-MSP1_42_ (3D7) IgG ELISA at Study Days 0, 29, 57, 85, 171, 256, and 340 will be evaluated. For each vaccine group and time point, the geometric mean titer and its standard deviation will be calculated and presented in tables and graphs. In addition, longitudinal mixed effects models will be utilized with log_10_-transformed antibody titers as the response variable and vaccine group and time since first vaccination as predictor variables. The effect of baseline variables such as age and gender on antibody kinetics will be explored with such models as well.

Additional analyses will calculate the area-under-the-curve (AUC) for antibody titers for each subject and compare average AUC between vaccine study arms.

A Cox proportional hazards model with log_10_-transformed antibody titer as a time-dependent covariate will be applied to the data on time to clinical malaria using the primary case definition to assess the association between antibody titers and subsequent risk of clinical malaria.

Similar analyses as described above will be applied to data on MSP1_42_ fragment- specific antibody titers.

For subjects vaccinated with FMP1/AS02A, the relationship between antibody levels measured by IgG ELISA and by competition ELISA will be explored through scatter plots and calculation of the correlation coefficients at each time point. Likewise, the relationship between antibody levels measured by growth inhibition assay and by ELISA will be assessed.

### Analysis of Safety

The overall percentage of subjects with at least one local AE (solicited and unsolicited) and the percentage with at least one general AE (solicited and unsolicited) during the seven-day follow-up period after vaccination will be tabulated. The incidence, intensity, and relationship of individual solicited symptoms over a seven-day period of follow‑up (day of vaccination, plus days 1, 2, 3, and 6) will be calculated for each study arm.

The number of subjects with at least one report of unsolicited AE reported up to 30 days after vaccination will be tabulated by study arm. The intensity and relationship to vaccination of the unsolicited symptoms reported will also be assessed.

SAEs are expected to be rare, but where observed will be described. Analysis of safety during the 12-month follow-up period (ten months after dose 3) will consist of comparison of incidence of SAEs as well as of changes in mean hemoglobin levels.

### Laboratory Parameters

To assess effects of FMP1/AS02A vaccination on hematology and biochemistry parameters, linear mixed effects models will be applied to data on complete blood counts (CBCs), creatinine, and ALT.

Descriptive statistics for the distribution of genotypes and multiplicity of infection will be generated by vaccine study arm.

## Administrative Matters

To comply with Good Clinical Practice (GCP), important administrative obligations relating to investigator responsibilities, monitoring, archiving data, audits, confidentiality, and publications must be fulfilled. These are outlined in Appendices D and E. Any missing, unused or spurious data, or previous reports on any deviations from the original statistical plan, will be cited and accounted for in the appropriate analysis (final or addendum; open or closed; blinded, partially unblinded, or unblinded) as they become apparent to study investigators or statisticians.

# ETHICAL CONSIDERATIONS

## Ethics and Regulatory Considerations

The study will be performed under FDA IND 9202. The study will be conducted according to Good Clinical Practice, US 21 CFR Part 50—Protection of Human Subjects, and Part 56—Institutional Review Boards, US Army Regulation AR 40-38, and AR 70-25 and local rules and regulations of the host country.

Primary monitoring responsibilities will be undertaken by PPD with GSK Biologicals as a co-monitor and USAMMDA as the study auditor. The term “study monitor” applies to designated representatives of any of these institutions. Scientific review committees of the WRAIR and KEMRI will have reviewed the study. In addition to the review by the Human Subjects Research Review Board (HSRRB) of the Office of the Surgeon General, US Army, the study will be reviewed and approved by the Kenya Medical Research Institute (KEMRI) Ethics Review Committee (ERC) according to the local laws and customs of Kenya. Documentation of this approval will be submitted to the HSRRB. The study will also be reviewed and approved by the Program for Appropriate Technology in Health (PATH) Human Subjects Protection Committee (HSPC). All other required approvals (initial, continuing review, amendments) must be secured from the primary IRB(s) prior to submission to the HSRRB.

### Institutional Review Board/Ethics Review Committee (IRB/ERC)

The IRB/ERC must be constituted according to the local laws and customs of Kenya and should include:

- At least five members.
- At least one member whose primary area of interest is non-scientific.
- At least one member who is independent of the sponsoring institution and study site.

Only those IRB/ERC members who are independent of the investigator and the study sponsor should vote and provide opinions on study-related matters.

A list of IRB/ERC members, or the IRB/ERC General Assurance Number, will be kept in the investigator’s study file.

This study is to be conducted in accordance with 21 CFR 56. All amendments will be submitted to the HSRRB through Office of Research Management, and to the KEMRI ERC, and to PATH HSPC. No amendment will go into effect without written approval from HSRRB, KEMRI ERC, and PATH HSPC except when the amendment is purely of an administrative nature or when the change is necessary to eliminate immediate hazards to study subjects. Administrative changes to the protocol are defined as corrections or clarifications that have no effect on the way the study is conducted. In such cases, the amendment will be submitted to the IRB/ERC and written verification that the administrative change was submitted will be obtained.

At a minimum the investigator must inform the IRBs/ERCs in a timely manner of the following:

- All subsequent revisions of study documents originally submitted for review
- All protocol amendments
- Any serious and unexpected adverse events occurring during the SAE reporting period
- Any significant deviations from the approved protocol procedures
- Where required, new information that may adversely affect the safety of the subjects or the conduct of the study
- Annual update or request for re-approval, where required, when the study has been completed

This study is to be conducted in accordance with 21 CFR 56, which requires that changes in approved research may not be initiated without IRB review and approval except where necessary to remove apparent immediate hazards to the human research subject.

## The Local Medical Monitor (Local Safety Monitor)

The Local Medical Monitor (LMM) will be an experienced, host nation physician based near the study site. The LMM must have the experience required to monitor human subjects during the conduct of a clinical trial and must be prepared to provide care to research subjects if any such need arises during the conduct of the study. In addition to the LMM, an alternate LMM, possessing the same qualifications, will be designated before study start. This alternate LMM will assume all the LMM’s responsibilities if the latter cannot meet his or her obligations.

The overall role of the LMM will be to vouchsafe responsible actions with regard to the ethics and clinical safety of a study, especially in regard to the proper assessment of AEs. At the same time the LMM should support the clinical investigators and act as a facilitator between them and the DSMB.

The PI will report all SAEs to the LMM, who will review them and provide an unbiased written report of events within ten calendar days of the initial report. At a minimum, this report will comment on the outcomes of the SAE, relationship to the test article, and indicate concurrence or non-concurrence with the details of the report provided by the PI.

Reports from the PI to the LMM detailing all SAEs and Grade 3 AEs for the seven day (vaccination day and six subsequent days), post-dose observation period, will also be provided to the DSMB, MVI, GSK Biologicals, and SCI following the administration of the first and second doses. This report will be submitted no less than seven days before the upcoming dose. After reviewing each report, the LMM will, if she concurs, recommend proceeding with the next scheduled dose. This concurrence may be given informally (e.g., telephonically) but will be recorded by the PI in the study regulatory file.

The LMM’s involvement will be particularly important when decisions have to be made quickly. Code break envelopes will be in the safekeeping of the LMM, who may unblind individual study subjects if necessary for medical or ethical reasons. In exceptional circumstances (e.g., a death possibly related to vaccination) the LMM would have the authority to suspend the whole or any specific aspect of the trial. The LMM may then call for trial resumption after discussion with, and approval by, the DSMB.

The LMM’s role will include:

- Providing advice to study investigators on whether a set of clinical circumstances in a study warrants formal notification of the DSMB, the study sponsor or others
- Providing clinical advice on any illness in study subjects especially in circumstances in which treatment might influence the course of the trial
- Reviewing all SAEs as outlined above
- Reviewing reports of all SAEs and Grade 3 solicited AEs (after Doses 1 and 2) and render concurrence/non-concurrence on subsequent doses as outlined above
- Make at least two visits to the WRP Kombewa Clinic to directly observe selected ongoing study operations

The LMM will work closely with the PI throughout the trial and relay any relevant safety information to the PI. It is the joint responsibility of the LMM and PI to convey this information to the sponsor, the DSMB, and MVI.

## The Data and Safety Monitoring Board (DSMB)

### Composition of the Board

For purposes of this study, the Data and Safety Monitoring Board (DSMB) is an independent committee consisting of up to five experts in malaria, infectious diseases, biostatistics, and other appropriate disciplines who are appointed to oversee the ethics and safety aspects of study conduct.

### Role of the Board

The DSMB may convene at any time during the study to review relevant safety data and to review and approve the Reporting and Analysis Plan (RAP). Meetings must be documented and minutes made available for the study files on site and to the sponsor. The DSMB may, if deemed necessary, convene a meeting with, or request further information from, the PI, the LMM, and designated project representatives of the WRAIR, GSK Biologicals, and MVI /PATH at any stage of the study; unscheduled meetings may be required.

The main role of the DSMB is to review and analyze clinical safety data collected during the trial and to assess SAEs.

The PI must provide the DSMB with:

- Prompt reports of any SAEs occurring during the study.
- Copies of LMM reports of Grade 3 solicited AEs after doses 1 and 2
- Any protocol amendments, informed consent changes or revisions of other documents originally submitted for review.
- Any new information that may adversely affect the safety of the subjects or the conduct of the study.

The DSMB can put the study on hold pending review of potential safety issues (see Section 6.9.2). All SAEs, including death, will be reported by the PI to the LMM, DSMB, the Manager of Clinical Safety Vaccines at GSK Biologicals, the HSRRB, KEMRI/ERC and MVI/PATH. The DSMB will review all SAEs for data trends in relation to safety issues and will have the right to seek additional clinical data about all cases. The DSMB will seek the input of the PI as needed. The DSMB may perform any of its own statistical calculations deemed necessary to support recommendations to the sponsor. All documentation provided to Board members for information and review must be treated in a confidential manner.

MVI will propose new members for the committee in the event that members must be replaced.

## Risks and Potential Benefits to Subjects

### Vaccination

Risks associated with vaccination include local inflammatory reactions to the injected product, such as injection site pain and swelling and some limitation of leg movement. Systemic effects may include flu-like syndrome, fever, nausea/GI symptoms, headache, malaise, myalgia, and arthralgia. To date all the symptoms associated with the FMP1/AS02A vaccine have been transient, mainly mild to moderate in intensity and have resolved without sequelae. While exceedingly rare, allergic reactions, including life-threatening anaphylaxis, are associated with many vaccine preparations and must therefore be considered as a potential risk in this study.

### Medical Treatment of Subjects

Free medical treatment of acute illnesses will be provided to all enrolled subjects during the active immunization phase and the follow-up period. The pharmacy at the WRP Kombewa Clinic is able to provide subjects with common over-the-counter analgesics such as paracetamol, as well as antibiotics for the treatment of minor infections. If further evaluation or treatment is necessary, the subject will be referred to the regional health facility (New Nyanza Provincial General Hospital (NNPGH), Kisumu. They will be transported by WRP vehicles (SOP No. 501.02). After duty hours, subjects will be able to receive medical attention at the study clinic or at this hospital. If in the judgment of the PI, or the clinical officer on duty, a subject requires hospitalization, referral to the NNPGH in Kisumu will be arranged.

Medical care for ailments not related to vaccination will not extend beyond the study period. Medical care for ailments related to vaccination will extend at least until the condition has resolved.

### Rabies Vaccination

During the study, subjects randomized to receive the rabies vaccine will benefit from this because of the high prevalence of rabies in Kenya. At the end of the study all subjects’ parents will be told which vaccine the subject received. Subjects randomized to the FMP1/AS02A vaccine will be offered rabies vaccine at that time. This later dosing will be done at the recommended schedule of 0, 7, and 21 (or 28) days.

## Precautions to Minimize Risk

### Vaccination

As outlined above, the subjects will be monitored closely during their participation in this study. The study vaccine has been prepared according to Good Manufacturing Procedures (GMP). The vaccines will be administered in the WRP KC under the supervision of an investigator with adequate drugs and equipment available for the treatment of anaphylaxis. All vaccine doses will be given by slow injection to minimize injection site reactions such as pain.

### Malaria Treatment During the Study

Medications available for the treatment of clinical malaria (see Section 7.3.3) will include oral *Coartem* and *Malarone.* Oral and parenteral quinine will also be available.

## Procedures for Maintaining Confidentiality

Subjects will be assigned a unique identifier number, the Subject Identification (SID) number. Each SID will include “FMP1” (the abbreviation for the antigen), the number “4” to indicate the fourth such study conducted within Africa, and four numerical digits between 0001 – *xxxx* to indicate the chronological order in which each subject was screened. Thus, the first subject screened for this study will be assigned the SID “FMP1-4-0001”, the fourth subject screened will be assigned the SID “FMP1-4-0004, and so on. All results will be linked to this number. Study records will only be available to staff members and will be kept in a secured area at the study site. Following the conclusion of the study, records will be maintained on site for a minimum of two years. These records may be reviewed by representatives of KEMRI, USAMRU-K, the USAMRMC, HSPC/PATH, the FDA, and the study sponsor as part of their responsibility to oversee this research.

# REFERENCES

1. Nchinda TC. Malaria: A reemerging disease. Emerging Infectious Dis. 1998; 4(3):398-403.
2. Breman, J. G. (2001). “The ears of the hippopotamus: manifestations, determinants, and estimates of the malaria burden.” *Am J Trop Med Hyg* 64(1-2 Suppl); 1-11.
3. WHO (1996). Technical Report Series 1857. Geneva, WHO: 1-13.
4. Snow, R.W., Craig, M., Deichmann, U. and Marsh, K. (1999a) Estimating mortality, morbidity and disability due to malaria among Africa's non-pregnant population, *Bull World Health Organ*, 77(8); 624-640.
5. Genton B, Betuela I, Felger I, et al. A recombinant blood-stage malaria vaccine reduces *Plasmodium falciparum* density and exerts selective pressure on parasite populations in a Phase I-IIb trial in Papua New Guinea. *JID, 2002; 185:820-7.*
6. Blackman MJ, Heidrich HG, Donachie S, et al. A single fragment of a malaria merozoite surface protein remains on the parasite during red cell invasion and is the target of invasion-inhibiting antibodies. J Exp Med 1990 July; 172:379-382.
7. Chang SP, Gibsoon HL, Lee-Ng CT, et al. A carboxyl-terminal fragment of *Plasmodium falciparum* gp195 expressed by a recombinant baculovirus induces antibodies that completely inhibit parasite growth. J Immunol July 1992; 149(2):548-555.
8. Egan AF, Morris J, Barnish G, et al. Clinical immunity to *Plasmodium falciparum* malaria is associated with serum antibodies to the 19-kDa C-terminal fragment of the merozoite surface antigen, PfMSP-1. J Infect Dis March 1996; 173:765-769.
9. Chang SP, Case SE, Gosnell WL, et al. A recombinant baculovirus 42-kilodalton c-terminal fragment of *Plasmodium falciparum* merozoite surface protein 1 protects *Aotus* monkeys against malaria. Infect Immun 1996 Jan; 64(1):253-261.
10. Ling IT, Ogun SA, and Holder AA. Immunization against malaria with a recombinant protein. Parasite Immunol 1994; 16:63-67.
11. Daly TM and Long CA. A recombinant 15-kilodalton carboxy-terminal fragment of *Plasmodium yoelii* 17XL merozoite surface protein 1 induces a protective immune response in mice. Infect Immun 1993 June; 61(6):2462-2467.
12. Daly T and Long CA. Humoral response to a carboxy-terminal region of the merozoite surface protein-1 plays a predominant role in controlling blood-stage infection in rodent malaria. J Immunol 1995; 155;236-243.
13. Burghaus PA, Wellde BT, Hall T, et al. Immunization of *Aotus nancymai* with recombinant C terminus of *Plasmodium falciparum* merozoite surface protein 1 in liposomes and alum adjuvant does not induce protection against a challenge infection. Infect Immun 1996 Sept; 64(9):3614-3619.
14. Crisanti A, Müller HM, Hilbich C, et al. Epitopes recognized by human T cells map within the conserved part of the GP190 of *P. falciparum*. Science 3 June; 1988; 240: 1324-1326.
15. Rzepczyk CM, Ramasamy R, Mutch DA, et al. Analysis of human T cell response to two *Plasmodium falciparum* merozoite surface antigens. Eur J Immunol 1989; 19:1797-1802.
16. GlaxoSmithKline Biologicals Data file.
17. Stoute JA. Unpublished results.
18. World Survey of Rabies, No. 34. 1998.
19. Dreesen DW, et al. Two-year comparative trial on the immunogenicity and adverse effects of purified chick embryo cell rabies vaccine for pre-exposure immunization. Vaccine. 1989; 7: 397-400.
20. Dreesen DW. Investigation of antibody response to purified chick embryo cell tissue culture vaccine (PCECV) or human diploid cell culture vaccine (HDCV) in healthy participants. Study synopsis 7USA401RA, September 1996 - December 1996 (unpublished).
21. Nicholson KG, et al. Pre-exposure studies with purified chick embryo cell culture rabies vaccine and human diploid cell vaccine: serological and clinical responses in man. Vaccine. 1987; 5: 208-210.
22. Vodopija I, et al. An evaluation of second generation tissue culture rabies vaccines for use in man: a four-vaccine comparative immunogenicity study using a pre-exposure vaccination schedule and an abbreviated 2-1-1 post-exposure schedule. Vaccine. 1986; 4: 245-248.
23. Wasi C, et al. Purified chick embryo cell rabies vaccine (letter). Lancet. 1986; 1: 40.
24. Cox JH, et al. Prophylactic immunization of humans against rabies by intradermal inoculation of human cell culture vaccine. J Clin Microbiol 1976;3:96-101.
25. Kuwert EK, et al. Some experiences with human diploid cell strain (HDCS) rabies vaccine in pre and post-exposure vaccinated humans. Dev Biol Stand 1978;40:79-88.
26. Ajjan N, et al. Resultats de la vaccination antirabique par le vaccin inactive concentre souche rabies PM/W138-1503-3M cultives sur cellules diploides humaines. Dev Biol Stand 1978; 40:89-100.
27. Costy-Berger F. Vaccination antirabique preventive par du vaccin prepare sur cellules diploides humaines. Dev Biol Stand 1978; 40:101-4.
28. CDC. Recommendations of the Immunizations Practices Advisory Committee (ACIP). Rabies Prevention United States. MMWR 1984, 33:393-402, 407-8.
29. Boe N., et al. Guillain-Barre after vaccination with human diploid cell rabies vaccine. Scand J Infect Dis 1980; 12:231-232.
30. CDC. Adverse reactions to human diploid cell rabies vaccine. MMWR 1980; 29:609-610.
31. Bernard KW, et al. Neuroparalytic illness and human diploid cell rabies vaccine. JAMA 1982: 248:3136-3138.
32. CDC. Systemic allergic reactions following immunization with human diploid cell rabies vaccine. MMWR 1984; 33:185-187.
33. Sharif SK, NA Kimathi and JD Quick, eds., *Clinical Guidelines for Diagnosis and Treatment of Common Hospital Conditions in Kenya*, Nairobi: Ministry of Health, Government of Kenya, 1^st^ Ed., Nov 1994; pp 103-7.
34. Bloland PB, Boriga DA, Ruebush TK, et al, “Longitudinal cohort study of the epidemiology of malaria infections in an area of intense malaria transmission II. Descriptive epidemiology of malaria infection and disease among children.” *Am J Trop Med* *Hyg* 1999 Apr; 60(4):641-8.
35. Hatz C, Abdulla S, Mull R, Schellenberg D, Gathmann I, Kibatala P, Beck HP, Tanner M, Royce C. Efficacy and safety of CGP 56697 (artemether and benflumetol) compared with chloroquine to treat acute falciparum malaria in Tanzanian children aged 1-5 years. Trop Med Int Health. 1998 Jun;3(6):498-504.
36. Falade C, Makanga M, Falade, Premji Z, Ortmann CE, Stockmeyer M, de Palacios PI. Efficacy and safety of artemether-lumefantrine (Coartem) tablets (six-dose regimen) in African infants and children with acute, uncomplicated falciparum malaria. Trans R Soc Trop Med Hyg. 2005 Jun; 99(6):459-67.
37. World Health Organization (2000). Severe falciparum malaria. *Transactions of the Royal Society of Tropical Medicine and Hygiene,* **94** (supplement 1): 1-90.
38. Okech BA, Corran PH, Todd J et al. Fine specificity of serum antibodies to Plasmodium falciparum merozoite surface protein, PfMSP-1(19), predicts protection from malaria infection and high-density parasitemia. . *Infect Immun*. 2004 Mar; 72 (3):1557-67.
39. Blackman MJ, Scott-Finnigan TJ, Shai S, Holder AA. Antibodies inhibit the protease-mediated processing of a malaria merozoite surface protein. J Exp Med. 1994 Jul 1;180(1):389-93.
40. Prudhomme JG, Sherman IW. A high capacity *in vitro* assay for measuring the cytoadherence of *Plasmodium falciparum*-infected erythrocytes. J Immunol Methods 1999; 229:169
41. Haynes JD and Moch K. Automated synchronization of *Plasmodium falciparum* parasites by culture in a temperature-cycling incubator. Methods Mol Med 2002; 72:489.

# STUDY BUDGET

## Study Budget Summary

| **Items** | **Amount in USD** | **Amount in Ksh** |
| --- | --- | --- |
| **Salaries** |  |  |
| ***Contract salaries (#89)*** | 558,788.12 | 44,703,049.60 |
| ***Casual (Field worker) salaries (#83)*** | 255,729.10 | 20,458,328.00 |
|  |  |  |
| **WRAIR Admin Overhead**  ***(Vaccines, shipping, CRC salary, etc)*** | 232,000.00 | 18,560,000.00 |
| **Subject Care** | 37,800 | 3,024,000.00 |
| **Lab Supplies** *(Clinical and Research)* | 155,737 | 12,458,960.00 |
|  |  |  |
| **Equipment** |  |  |
| ***Laboratory*** | 64,106.00 | 5,128,480.00 |
| ***IT*** | 28,384.00 | 2,270,720.00 |
| ***Three 4WD Vehicles (Land Cruisers)*** | 75,000.00 | 6,000,000.00 |
|  |  |  |
| **Travel, Training and Related fees** | 20,800.00 | 1,664,000.00 |
| **Kombewa utilities** *(Fuel, electric, commo, etc)* | 70,483.00 | 5,638,640.00 |
| **Other expenses** | 10,500.00 | 840.000.00 |
|  |  |  |
| **Total Projected Phase IIb Expenses (Original)** | 1,509,327.22 | 120,746,177.00 |
| **Total plus KEMRI Overhead (10%)** | 1,660,259.94 | 132,820,795.36 |
|  |  |  |
| ***4 Month Study Extension*** |  |  |
| *Salary impact* | 264,509.59 | 21,160,767.20 |
| *Incidentals/overhead impact* | 30,000.00 | 2,400,000.00 |
| **4 Month study extension: Total** | 294,509.59 | 23,560,767.20 |
|  |  |  |
| ***1 Month Study Extension*** |  |  |
| *Salary impact* | 66,127.39 | 5,290,191.20 |
| *Incidentals/overhead impact* | 7,500.00 | 600,000.00 |
| **1 Month study extension: Total** | 73,627.39 | 5,890,191.20 |
|  |  |  |
| **Grand Total w/ Extensions** | 2,028,396.92 | 162,271,753.76 |

NB: No attribution as to funder is intended for any subsection presented.

## Budget Justification

The above budget summary was prepared using expense records from previous studies in western Kenya, which have utilized similar numbers of subjects. The “study extensions” are occasioned by unanticipated delays in study start with the necessity of holding over contract employees from the previous MSP-1 trial.

# APPENDICES

## Appendix A: Study Personnel

### Roles of Study Personnel

| **Investigator** | **Title** | **Responsibility** |
| --- | --- | --- |
| Bernhards Ogutu, MBChB, Mmed (Peds), PhD * | Principal Investigator | Responsible for overall execution, protocol preparation, clinic procedures, follow-ups, data analysis, and manuscript preparation |
| Mark R. Withers, MD, MPH* | Associate Investigator | Protocol preparation, data analysis, oversight of all WRP Kisumu operations |
| Mark E. Polhemus, MD | Associate Investigator | Clinical evaluations, data analysis, and assist with oversight of WRP Kisumu operations |
| Lucas Otieno, MBChB | Associate Investigator | Clinical evaluations, overall supervision of junior physicians and clinical officers |
| John N. Waitumbi, PhD | Associate Investigator | Oversees all immunological assays |
| Shon A. Remich, MD | Associate Investigator | Responsible for overall WRP KC operations, clinical evaluations |
| D. Gray Heppner, MD | Associate Investigator | Protocol preparation, data analysis |
| Chris Ockenhouse, MD, PhD | Associate Investigator | Oversees genetics investigations |
| Denise McKinney, RN, CCRC, CCRA | Associate Investigator | Protocol and vaccine preparation, randomization, regulatory oversight, oversees CRCs |
| Odika J. Apollo, RCO | Research Coordinator | Oversees subject visits, protocol compliance, translation, staff training |
| Melanie Onyango, BSc, CCRC | Research Coordinator | compliance, translation, staff training |
| Stacey M. Gondi, RCO | Asst. Research Coordinator | compliance, translation, staff training |
| Jessica Milman, MPH | Contributor, Funder | Protocol preparation, data analysis |
| Willis Okoth, BSc | Contributor | Measurement of anti-MSP-1 antibody |
| Joram Siangla | Contributor | Measurement of anti-MSP-1 antibody |
| Jeff Lyon, PhD | Contributor | Vaccine production |
| Evelina Angov, PhD | Contributor | Vaccine production |
| David Haynes, PhD | Contributor | Performance of growth inhibition assay |
| Carolyn Holland, MPH | Contributor | Logistical coordination, WRAIR |
| Ezekiel Oenga, BPharm | Contributor | Clinic pharmacist, drug management and vaccine preparation, randomization |
| Amanda Leach, MSc, MRCPCH | Contributor | Protocol preparation, data analysis |
| Carter Diggs, MD, PhD | Contributor | Protocol preparation, data analysis |
| Lorraine A. Soisson, PhD | Contributor | Protocol preparation, data analysis |
| Kathryn Tucker, MS | Statistician | Statistical analysis of database, and preparation of CRFs, randomization schedule, analysis plan, and statistical reports |
| Janet Wittes, PhD | Statistician |
| Wasima Rida, PhD | Statistician |
| Raphael Pundo, MSc | Contributor | Database creation, data entry |

*CVs for selected Study Personnel are maintained in the Study Regulatory File.

## Appendix B: List of Study Specific Procedures (SSPs)

1. SSP No. MAL036-01: Recruitment and Informed Consent Process

2. SSP No. MAL036-02: Screening Day Procedures

3. SSP No. MAL036-03: Randomization

4. SSP No. MAL036-04: Vaccination Day Procedures

5. SSP No. MAL036-05: SAE Reporting

6. SSP No. MAL036-06: Internal Quality Control Plan for CRF Review

7. SSP No. MAL036-07: Breaking the Study Blind

8. SSP No. MAL036-08: Internal Quality Control for Database Review

9. SSP No. MAL036-09: Vaccine Preparation and Administration

10. SSP No. MAL036-10: Vaccine Storage and Packaging for Transport

11. SSP No. MAL036-11: Comprehensive Overview for MBF Data Entry

12. SSP No. MAL036-12: Field Worker Home Visits

13. SSP No. MAL036-13: Scheduled Clinic Visits

14. SSP No. MAL036-14: Provisions for Suspending Vaccination to Review Adverse Events

15. SSP No. MAL036 DE 1.000: Data Management

## Appendix C: List of Mal-036-Relevant Standard Operating Procedures (SOPs)

Laboratory SOPs:

1. SOP No. KCL 002: Emergency Phlebotomy Safety Procedures
2. SOP No. KCL 003: Finger-Prick for Blood Smear
3. SOP No. KCL 005: Laboratory Hazards and Safety Procedures
4. SOP No. KCL 006: Hazard Communication and Chemical Hygiene
5. SOP No. KCL 007: Bloodborne and Infectious Control
6. SOP No. KCL 103: Coulter AcT 5Diff CP – Blood Sample Analysis
7. SOP No. KCL 205: ACE Wasserman - Test Requisition
8. SOP No. KCL 205: ACE Wasserman – Running the Tests
9. SOP No. KSM 221.0: Procedure for PCR Amplification of G6PD Gene
10. SOP No. KSM 229.0: Procedure for Alpha-thalassemia PCR Amplification
11. SOP No. KSM 230.0: Procedure for Analysis of Hemoglobin Variants in Whole Blood
12. SOP KCL 301: Malaria Blood Films – Blood Sample Reception and Smear Preparation
13. SOP KCL 305: Malaria Blood Films – Reading and Quantification of Malaria Parasites
14. SOP KCL 304: Slide Coordination and MBF Results Interpretation
15. SOP KCL 307: Malaria Blood Films – Quality Control and Quality Assurance
16. SOP KCL 306: Malaria Blood Films – Documentation and Storage of MBF
17. SOP KCL 302: Preparation of Buffer Solution
18. SOP KCL 303: Preparation of Working Giemsa and Staining
19. SOP KCL 008: Processing of Laboratory Blood Specimens
20. SOP KCL 017: Microscope Maintenance
21. SOP KCL 012: pH meter Calibration
22. SOP No. KSM 201.1: SOP for Validation and QC of 3D7 MSP-42 Human ELISA
23. SOP No. KSM 202.0: SOP for Test Samples of 3D7 MSP-42 Human ELISA
24. SOP No. KSM 220.0: SOP for MSP-1_42_ Allotyping by Real Time PCR

General/Administrative SOPs:

1. SOP No. KOM 101.01: Field Worker Evaluation
2. SOP No. KOM 401.01: Investigational Products Accountability
3. SOP No. KOM 201.01: Administration of Informed Consent
4. SOP No. KOM 501.02: Referral of Study Subjects for Medical or Dental Care
5. SOP No. KOM 501.10: Adverse Events Reporting
6. SOP No. KOM 501.03: After-Hours Care of Study Subjects
7. SOP No. KOM 402.06: Immediate Notification of Principal Investigator
8. SOP No. KOM 201.03: Documentation and Reporting of Clinical Protocol Deviations
9. SOP No. KOM 201.04: CRF Completion and Correction

Clinical SOPs:

1. SOP No. KOM 501.08: Diagnosis and Treatment of Malaria
2. SOP No. KOM 501.03: After Hours Care of Subjects
3. SOP No. KOM 501.05: Management, Documentation and Reporting of Pediatric Vital

Signs Outside Established Normal Ranges

1. SOP No. KOM 501.07: Recognition and Treatment of Anaphylaxis in Children

Product SOPs:

1) SOP No. KOM 301.03: “Vaccine and/or Drug Refrigerator Monitoring and Maintenance”

2) SOP No. KOM 301.04: “Vaccine and/or Drug Refrigerator Failure”

## Appendix D: Overview of Study Operational Scheme

### Operational Study Milestones

All dates are hypothetical and provisional and are intended for planning purposes only.

Begin recruiting: 6 Dec 2004

Begin screening: 10 Jan 2005

Dose 1: 7-11 Feb 2005 (about 57 subjects/day for 7 days)

Dose 2: 28 Feb – 4 Mar 2005 (about 57 subjects/day for 7 days)

Dose 3: 28 Mar – 1 Apr 2005 (about 57 subjects/day for 7 days)

Efficacy Follow-up Period: 11 Apr to 27 Sep 2005 (o/a SD 71 to SD 240)

Addendum Efficacy Follow-up Period: 27 Sep to 10 Feb 2006 (o/a SD 240 to SD 364)

Intention-to-treat Follow-up Period: 7 Feb 2005 to 10 Feb 2006 (SD 0 to SD 364)

### Study Recruitment Plan

Four hundred (400) healthy subjects aged 12 to 47 months will be recruited by non‑coercive means according to existing U.S. Army regulations (AR 70-25 and AR 40-38).

**The aims and objectives of the study will be thoroughly discussed with local community leaders prior to the start date. An historical census (August - October 2001) of all of Kombewa District will be utilized. A limited update of this census will be conducted in two to four months prior to commencement of screening.**

| Exhibit 13. A selected portion of the pediatric population of Kombewa District, Kisumu Division, Western Kenya. Only children living (1) within one mile of 22 provisional field stations (See Exhibit 14 and attached maps) and (2) falling into three age groups (12-23, 24-35, and 36-47 months) are presented. These data are taken from a “Walter Reed Project” census performed by Mr. Shadrack Odera and others from Aug to Oct 2001, and reflect the demographic reality at that time. The total adult and pediatric population of Kombewa District was found to be approximately 63,000. | | | | | |
| --- | --- | --- | --- | --- | --- |
| **Number of Family Compounds with** | | | | | |
| **Age (mo)** | **1 Child** | **2 Children** | **3 Children** | **4 Children** | **Total** |
| 12-23 | 1191  (1191 children) | 120  (240 children) | 14  (42 children) | 4  (16 children) | (1479 children) |
| 24-35 | 1429  (1429 children) | 151  (302 children) | 25  (75 children) | 3  (12 children) | (1744 children) |
| 36-47 | 1146  (1146 children) | 114  (228 children) | 13  (39 children) | 0  (0 children) | (1335 children) |
| All | 3766  (3766 children) | 385  (770 children) | 52  (156 children) | 7  (28 children) | (4558 children) |

Children who met age and other criteria 3 ½ years ago were identified as indicated in Exhibit 13. Additionally, children who were in the ages of interest are indicated in Exhibit 14, which summarizes the pediatric populations within one-mile radius of 22 proposed field sites. As some provisional field sites have radii that overlap, these numbers are somewhat inflated; the grand total of 6,371 children shown (Exhibit 14) is about 40% higher than the actual total of 4,558 (Exhibit 13). Locations of Kombewa field stations are indicated on the map in Figure C. Locations of children within the stated radii are shown, according to age group, in the maps in Figures D, E and F.

| Exhibit 14. A selected portion of the pediatric population of Kombewa District, Kisumu Division, Western Kenya. Only those children living within one mile of 12 proposed field stations (See Exhibit 13 and attached maps) and falling into three age groups are presented. The grand total number is about 28% inflated due to catchment overlap (i.e., some proposed field sites are less than a mile apart). | | | | | |
| --- | --- | --- | --- | --- | --- |
|  | **Field Stations** | Ages (months) | | | |
|  |  | 12-23 | 24-35 | 36-47 | Totals |
| 1. | WRP Kombewa Clinic | 155 | 159 | 132 | 446 |
| 2. | Abol Primary School | 116 | 173 | 117 | 406 |
| 3. | Lieye Primary School | 125 | 156 | 114 | 395 |
| 4. | Jonyo Primary School | 110 | 153 | 103 | 366 |
| 5. | Chwa Polytechnic School | 118 | 116 | 101 | 335 |
| 6. | Bar Korwa Primary School | 106 | 112 | 110 | 328 |
| 7. | Alwala Primary School | 103 | 120 | 92 | 315 |
| 8. | Rabongi Primary School | 90 | 118 | 98 | 306 |
| 9. | Rapogi Nursery School | 97 | 132 | 75 | 304 |
| 10. | Ngere Primary School | 102 | 115 | 73 | 290 |
| 11. | Nyaundi Primary School | 93 | 118 | 77 | 288 |
| 12. | Kitare Primary School | 89 | 108 | 88 | 285 |
| 13. | Kondik Market | 82 | 102 | 94 | 278 |
| 14. | Korumba Primary School | 93 | 93 | 72 | 258 |
| 15. | Nyawanga Primary School | 90 | 95 | 68 | 253 |
| 16. | Kolenyo Market | 74 | 89 | 76 | 239 |
| 17. | Ang’oga Market | 67 | 103 | 69 | 239 |
| 18. | Kopingo Market | 82 | 77 | 76 | 235 |
| 19. | Ranen Primary School | 82 | 86 | 64 | 232 |
| 20. | Oswre Primary School | 74 | 83 | 57 | 214 |
| 21. | Oruga Primary School | 62 | 82 | 57 | 201 |
| 22. | Ramuya Primary School | 46 | 73 | 39 | 158 |
|  | **TOTALS** | 2056 | 2463 | 1852 | 6371 |

Prior to screening, the parental consent explanation forms will be distributed to the parents of prospective subjects and study objectives and procedures will be explained to them in their primary language via a video- or audiotaped presentation. Recruitment will be incremental until the desired number of subjects is enrolled, or up to 25 days after the first dose of vaccine is given. Subjects will be randomized to a study arm at the time of first vaccination.

Four hundred children from 12 to 47 months of age will be recruited from among the 9 of the 22 provisional field sites indicated in Exhibit 14, as well as 3 additional field sites yet to be identified.

### Study Operational Field Plan

**A. Activities at Twelve (12) Field Stations**

With 12 field stations (FSs) in use for this phase IIb study, and 400 total subjects, approximately 34 subjects will be followed at each station. This estimate is based upon the 25% recruitment rate experienced in Mal-031 where approximately 34 12 to 47 month olds could be successfully recruited from within one mile of each FS on average. This implies a need for 12 FSs for a study population of 400 subjects. (A more optimistic estimate would be derived from the 50% recruitment rate experienced with the WRAIR Protocol #1020 malaria epidemiology study ongoing at Kombewa. In that study, an average of 45 subjects could be obtained per FS. This greater success rate would imply a need for nine FSs. It may be that the non-interventional nature of this epidemiology trial encouraged the higher levels of subject/ parent participation, compared with the vaccine study.)

An effort will be made to select subjects in roughly equal numbers from each site. While the monthly scheduled study visits are to be undertaken at the WRP KC, it is anticipated that parents will need to bring a sick or symptomatic child to an FS at night or at other inconvenient times. Kombewa FSs are intended to be contact points for subjects and their parents with WRP staff and are easily accessible by travel on foot. A parent with a child might reasonably be expected to walk a mile in 30 minutes or so, and thus, FSs were evaluated with this transit time in mind. Kombewa FSs are intended to be occupied by a WRP field worker 24 hours per day throughout the study period and, as such, they will all represent a physically secure (lockable) room at a school, market or church and will have basic overnight supplies, such as radio handset, mobile telephone (where service is available), basic medical kit (bandages, latex gloves), kerosene lantern, flashlight, batteries, raincoat, blanket, rubber boots, folding cot, mosquito net, insecticide spray, and padlock with keys. At the WRP KC a unit mobile phone battery charger will be available for field worker use.

Seven field workers (FWs) will be assigned to each FS, necessitating the employment of 84 FWs for this study. This number gives a ratio of 4.8 subjects to each FW and 33 subjects to each FS. On-duty FWs, while not usually medically trained personnel, are each answerable not only to a senior FW, but to an on duty Clinical Officer (Kenyan equivalent of physician assistant) or to a physician. An on-call schedule will be devised, such that, with one FW on duty at the station and one FW off duty (recovering from night call the night before), a minimum of five FWs will be available for home visits on any given day (assuming no sick days are taken). New FWs will undergo a 2 to 3 week training period where they will be exposed to essential medical aspects of the present study, the operational plan for the FWs involved in the study, and the logistics of field station duty (radio communication, transportation, etc.).

Approximately nine 4WD ground vehicles will be permanently stationed at the WRP KC; eight to make daily rounds to the FSs involved in the study and one as a dedicated ambulance. Ambulance drivers will be equipped with a dedicated vehicle radio and a cell phone, and two drivers/vehicles are on duty on-site after hours and on weekends. Additionally, a senior, supervising FW, clinical officer (CO), nurse, and laboratory technician are on call after hours and on weekends. One physician will be on call at all times. (We anticipate the need to hire two additional physicians, three COs, three community nurses, three drivers, and to purchase three additional 4WD vehicles.)

B. Activities at Three Field Follow-up Centers

Three of the twelve Field Stations (FSs), selected for their strategic locations, have also been designated as Field Follow-up Centers (FFCs). On post-vaccination days 1 to 3, 6 (Study days 1, 2, 3, 6, 30, 31, 32, 35, 58, 59, 60, and 63) a CO, nurse, and senior field worker will be assigned to duty at each FFC. Subject follow-up activities for these days will be undertaken at these FFCs as well as at the WRP KC. Activities will include the measurement of temperature, pulse, and assessment of subject signs and symptoms only. No medical care will be provided; rather, any subject who presents unwell will be transported to WRP KC for definitive care and treatment.

## Appendix E: Administrative Matters

### General Administrative Matters

**I. Responsibilities of the Investigator**

- To ensure that he/she has sufficient time to conduct and complete the study and has adequate staff and appropriate facilities which are available for the duration of the study and to ensure that other studies do not divert essential subjects or facilities away from the study at hand.
- To submit an up-to-date curriculum vitae and other credentials (e.g. medical license number in the United States or Kenya) to the sponsor and—where required—to relevant authorities.
- To acquire the normal ranges for laboratory tests performed locally and, if required by local regulations, obtain the Laboratory License or Certification.
- To prepare and maintain adequate case histories designed to record observations and other data pertinent to the study.
- To conduct the study in compliance with the protocol and appendices (See Section 16).
- To cooperate with a representative of the sponsor in the monitoring process of the study and in resolution of queries about the data.

**II. Protocol Amendments and modifications**

This study is to be conducted in accordance with 21 CFR 56. Any amendment to the protocol will be adhered to by the participating center and will apply to all subjects. Written IRB/IEC approval of protocol amendments is required prior to implementation. All amendments will be submitted to the HSRRB through Office of Research Management, and to the KEMRI ERC, and to PATH HSPC. No amendments will go into effect without written approval from HSRRB, KEMRI ERC, and PATH HSPC except when the amendments are purely of an administrative nature or when the changes are necessary to eliminate immediate hazards to the subjects.

**III. Sponsor’s Termination of Study**

The sponsor reserves the right to discontinue the clinical study at any time for medical or administrative reasons. When feasible, a 30-day written notification will be tendered.

1. **Case Report Form Instructions**

Prior to screening the first potential subject, the investigator will provide a list showing the signature and hand-written initials of all individuals authorized to make or change entries on case report forms (CRFs). If the authorized individuals should change during the study, the investigator will update the signature list.

CRFs will be designed by Statistics Collaborative, Inc., for recording all data. It is the responsibility of the PI or AI, and CRC, to ensure that CRFs are legible and completely filled in with a blue or black ink ballpoint pen.

Errors must be corrected by drawing a single line through the incorrect entry and writing in the new value/data positioned as close to the original as possible. The correction must then be initialed, dated and justified, where necessary, by the authorized individual making the change. The original entry must not be obliterated, overwritten, or erased when a correction is made.

The investigators or designated staff will make every effort to complete the relevant sections of the CRF as soon as possible following a visit. Similarly, when a subject completes the study, every effort will be made to complete the CRF as soon as the last data become available.

As soon as the subject has completed/withdrawn from the study and the CRFs are completed, the PI or AI (s) under his/her supervision will sign the study conclusion CRF to confirm that they have reviewed the data and that the data are complete and accurate.

A CRF must be prepared for all subjects who have undergone protocol‑specific procedures, whether or not the subject completed the study.

While completed CRFs will be reviewed by a professional monitor at the study site, errors detected by subsequent in-house CRF review may necessitate clarification or correction of errors and documentation and approval by an investigator. Whenever needed, the PI or AI should promptly assist in clarification or correction of errors detected after study finalization.

1. **Monitoring by PPD**

See Section 16.4.2, part I, “Monitoring/Quality Assurance”.

1. **Archiving of Data**

The investigator/ institution should maintain all study documentation (including regulatory and sponsor-specific documents) until at least two years after the last approval of a marketing application in an ICH region and until there are no pending or contemplated marketing applications in an ICH region, or until at least two years have elapsed since the formal discontinuation of the clinical development of the investigational product. These documents should be retained for a longer period, however, if required by the applicable regulatory requirements or by an agreement with the sponsor. The sponsor should inform the investigator/institution in writing of the need for record retention and should notify the investigator/institution in writing when the study-related records are no longer needed.

The investigator/ institution should take measures to prevent accidental or premature destruction of these documents.

1. **Auditing by a Drug Regulatory Agency**

For the purpose of compliance with Good Clinical Practice and Regulatory Agency Guidelines it may be necessary for a Drug Regulatory Agency to conduct a site audit. This may occur at any time from start to after conclusion of the study.

When an investigator signs the protocol, he agrees to permit Drug Regulatory Agencies and the sponsor access to subject records. Furthermore, if an investigator refuses an inspection, his data will not be accepted in support of a New Drug Registration and/or Application.

The Inspector will be especially interested in the following items:

- Log of visits from the sponsor's representatives
- IRB/IEC approvals
- Vaccine accountability
- Approved study protocol and amendments
- Informed consent of the subjects (written or witnessed oral consent)
- Medical records supportive of CRF
- Reports to the IRB/IEC and the sponsor
- Record retention

### USAMRMC Specific Administrative Procedures

**I. Monitoring/Quality Assurance**

Monitoring of this protocol will be performed by representatives of the Pharmaceutical Product Development (PPD) with the assistance of a co-monitor, GSK Biologicals. These monitoring activities will be audited by USAMMDA Quality Assurance. Study monitors will conduct a study initiation visit, at least one interim study visit, and a study close-out visit (at approximately month 8 or 13). The monitor will review CRFs to verify accurate data collection, will evaluate adherence to Good Clinical Practices, and ensure completeness, accuracy, and integrity of study data. The PI must ensure provision of reasonable time, space, and adequate qualified personnel for monitoring visits.

After study completion and closure, the regulatory documents file (including documentation of test article storage, inventory, and accountability), and subject records will be maintained in the archives of the Walter Reed Project Kisumu. Consent forms along with a copy of the final approved protocol will be retained indefinitely.

The volunteer registry data sheets (VDRSs) will be collected and submitted to the Office of Research Protections (ORP) at the conclusion of the study (after unblinding). It is the policy of the U.S. Army Medical Research and Materiel Command that these data sheets are to be completed on all subjects participating in research for entry into this Command’s Volunteer Registry Data Base. The information to be entered into this confidential database includes the name, address, Social Security number, study name and dates. Where Social Security numbers are not applicable, the National Identification number may be substituted, if available. The intent of the data base is two-fold: first, to readily answer questions concerning an individual’s participation in research sponsored by USAMRMC; and second, to ensure that the USAMRMC can exercise its obligation to ensure research volunteers are adequately warned (duty to warn) of risks and to provide new information as it becomes available. The information will be stored at USAMRMC for a minimum of 75 years.

**II. Evaluations During and Following the Project**

The medical evaluations of subjects will be recorded by one of the physician investigators on standard forms. Appropriately trained individuals will obtain blood samples for antibody tests. A copy of volunteer registry data sheets (VRDS) will be kept on file at a USAMMDA-approved site for at least ten years after completion of the study. Consent forms along with a copy of the final approved protocol will be retained indefinitely. The Subject VDRS will be collected and submitted to the Office of Research Protections (ORP) at the conclusion of the study.

**III. Withdrawal from Protocol for Individual Subjects**

Subjects will be allowed to withdraw from the study at any time. Parents will be requested to inform a member of the study staff should they choose to withdraw their child from the study, and the reason(s) for withdrawal will be solicited. No further procedures will be required of the study subjects/parents.

**IV. Protocol amendments**

(See Section 16.4.1, part II)

**V. Disposition of Unused Medications**

Unused investigational vaccine doses will be accounted for and will be returned to the manufacturer for safekeeping or disposed of according to the manufacturer’s policy.

**VI. Use of Information and Publications Arising From this Study**

It is anticipated that the results of this study will be presented to the scientific community via oral presentations at meetings and written publications in scientific journals. The official final report will be submitted through appropriate channels, and upon approval by the WRAIR, Dept. of Immunology to KEMRI SSC/ERC, the Regulatory Affairs Branch of the ORP and to the HSRRB. This report will contain detailed information about the subjects, their tolerance of the vaccines, their side effects and laboratory abnormalities, and vaccine efficacy, or lack thereof.

## 16.6 Appendix F: World Health Organization Criteria for Severe Falciparum Malaria

### 16.6.1 Severe Manifestations of P. falciparum malaria (WHO, 2000, pp S/1-2)

“In a patient with *P. falciparum* asexual parasitemia and no other confirmed cause for their symptoms the presence of one or more of the clinical or laboratory features in Table 1 [here, Section 16.6.2] classifies the patient as suffering from severe malaria.”

### EXTRACT: Definition of Severe Falciparum Malaria (WHO, 2000, pp S1/1-11)

|  | **Clinical manifestations** (WHO, 2000, pg S1/2, Table 1; Corrected version insert): | **Remarks from text** (WHO, 2000, pg S1/1-11): |
| --- | --- | --- |
| 1. | Prostration | “The inability to sit unassisted in a child normally able to do so.” |
| 2. | Impaired consciousness | For example: “Using the Blantyre coma scale for children with a coma score ≤ 3” |
| 3. | Respiratory distress (acidotic breathing) | “The presence of the following individual components should be specified for research purposes” … “sustained nasal flaring” … “indrawing (recession) of the bony structure of the lower chest wall” … “deep breathing (acidotic breathing, Kussmaul breathing)” |
| 4. | Multiple convulsions | Children with 2 or more convulsions within a 24 hour period” |
| 5. | Circulatory collapse | “The diagnosis must be based on a combinat-ion of hypotension and poor perfusion (cool peripheries, especially mid to proximal limb coolness, and weak or absent peripheral pulses)” |
| 6. | Pulmonary oedema (radiological) | “Diagnosis … really requires a chest X-ray… The radiological features of progressive pulmonary oedema in children are: increased interstitial markings; hazy peri-hilar interstitial shadowing; frank air-space disease with focal or conglomerate shadowing and air broncho-grams; and ‘bat’s wing’ shadowing and Kerly B lines with or without pleural effusion” |
| 7. | Abnormal bleeding | “mild abnormalities of laboratories are common and, in the absence of clinical evidence of bleeding, are not in themselves an indication of severe disease” |
| 8. | Jaundice | “detected clinically” |
| 9. | Haemoglobinurea | “when the urine is dark… and the … dipstick … is positive…the absence of microscopic haematuria suggests either haemoglobinuria or myoglobinuria” |

|  | **Laboratory findings** (WHO, 2000, pg S1/2, Table 1; Corrected version insert): | **Remarks from text** (WHO, 2000, pg S1/1-11): |
| --- | --- | --- |
| 1. | Severe anemia | “defined as haemoglobin <5 g/dL or haematocrit < 15%. It should be specified whether results are from a finger prick or venous sample…” |
| 2. | Hypoglycaemia | “defined as whole blood glucose concent-ration of less than 2.2 mmol/L (less than 40 mg/dL)…. For research purposes hypo-glycaemia should be confirmed on a venous sample measured with a glucose analyzer” |
| 3. | Acidosis* | “plasma bicarbonate concentration (<15 mmol/L) or base excess (>-10). The acid-emia… is defined as pH <7.35 measured in capillary or arterial blood” |
| 4. | Hyperlactatemia* | “A level > 5 mmol/L is an indication of severe malaria” |
| 5. | Hyperparasitemia | “In areas of stable endemicity, threshold levels should be derived from local experience but, in the absence of data, a parasitemia > 20% should be considered to indicate severe malaria” |
| 6. | Renal impairment | “defined as a urine output of less than 12 mL/kg/24 h or a plasma creatinine concentration above the age-related normal range, persisting after rehydration” |

* NB: Blood gases and blood lactate levels are generally unavailable at the Kenya study site.

***Adapted from*** World Health Organization (2000). Severe falciparum malaria. *Transactions of the Royal Society of Tropical Medicine and Hygiene,* **94** (supplement 1): 1-90.

### World Health Organization (2000). Severe falciparum malaria

[Section 16.6.3 is the article: World Health Organization (2000). Severe falciparum malaria. *Transactions of the Royal Society of Tropical Medicine and Hygiene,* **94** (supplement 1): 1-90 which is appended in it’s entirety to the electronic version of the protocol, V7a.]
